# Proteogenomic Analysis of *Mycobacterium smegmatis* Using High Resolution Mass Spectrometry

**DOI:** 10.3389/fmicb.2016.00427

**Published:** 2016-04-05

**Authors:** Matthys G. Potgieter, Kehilwe C. Nakedi, Jon M. Ambler, Andrew J. M. Nel, Shaun Garnett, Nelson C. Soares, Nicola Mulder, Jonathan M. Blackburn

**Affiliations:** ^1^Computational Biology Division, Department of Integrative Biomedical Sciences, IDM, University of Cape TownCape Town, South Africa; ^2^Division of Chemical and Systems Biology, Department of Integrative Biomedical Sciences, IDM, University of Cape TownCape Town, South Africa

**Keywords:** *Mycobacterium smegmatis* mc^2^155, mass spectrometry, proteogenomics, genome annotation, proteomics

## Abstract

Biochemical evidence is vital for accurate genome annotation. The integration of experimental data collected at the proteome level using high resolution mass spectrometry allows for improvements in genome annotation by providing evidence for novel gene models, while validating or modifying others. Here, we report the results of a proteogenomic analysis of a reference strain of *Mycobacterium smegmatis* (mc^2^155), a fast growing model organism for the pathogenic *Mycobacterium tuberculosis*—the causative agent for Tuberculosis. By integrating high throughput LC/MS/MS proteomic data with genomic six frame translation and *ab initio* gene prediction databases, a total of 2887 ORFs were identified, including 2810 ORFs annotated to a Reference protein, and 63 ORFs not previously annotated to a Reference protein. Further, the translational start site (TSS) was validated for 558 Reference proteome gene models, while upstream translational evidence was identified for 81. In addition, N-terminus derived peptide identifications allowed for downstream TSS modification of a further 24 gene models. We validated the existence of six previously described interrupted coding sequences at the peptide level, and provide evidence for four novel frameshift positions. Analysis of peptide posterior error probability (PEP) scores indicates high-confidence novel peptide identifications and shows that the genome of *M. smegmatis* mc^2^155 is not yet fully annotated. Data are available via ProteomeXchange with identifier PXD003500.

## Introduction

Evidence for the existence of protein coding genes include *ab initio* gene predictions, transcriptomic analysis, and comparative genomics information (Krug et al., 2011). Although, gene annotation of model organisms often relies on transcript sequencing, it has become apparent that evidence of transcription may not equal evidence of translation (Castellana and Bafna, 2010). Proteomics data, on the other hand, gives direct evidence of which genes are translated. Using proteomics data, true genes can be separated from pseudogenes, and the translational frame can be determined. The location of TSSs (translational start sites) can be identified, while signal cleavage and other post-translational modifications (PTMs) can be identified (Kucharova and Wiker, 2014). By mapping to uncharacterized genomic coordinates, novel genes can be identified (Borchert et al., 2010).

Genomic six frame translation allows for the creation of a database containing, in the ideal case, all possible putative proteins, but at the cost of including many spurious entries. This leads to decreased sensitivity of identifications at the same FDR as standard proteomic databases with a higher proportion of non-spurious entries (Castellana and Bafna, 2010). Due to the large database sizes involved in proteogenomics, and large numbers of spectra that need to be assigned, many false positive identifications are obtained even at a low error rate. To limit the number of spurious entries, thus increasing the sensitivity of identifications, proteogenomic databases can be compacted, by excluding entries below a minimum length cutoff or only focusing on genomic regions identified by *ab initio* gene prediction tools (Castellana and Bafna, 2010).

Automated gene prediction at the early stage of genome annotation is prone to errors, with rates of incorrect TSS prediction of up to 44% reported (Gallien et al., 2009), while short protein-coding genes are difficult to predict (Renuse et al., 2011). Accurately identifying the TSS of a gene is complicated by the existence of different possible start codons, with many non-standard start codons identified in prokaryotes (Castellana and Bafna, 2010). Mycobacteria are known to use GTG and TTG as initiator *Met* (*fMet*) start codons, in addition to translation of these codons with *Val* and *Leu*, respectively (Kelkar et al., 2011).

Wang et al. (2005) used databases of gene predictions to identify mass spectra, identifying 901 proteins in *Mycobacterium smegmatis* using the partially sequenced genome available at the time—validating many predicted genes. Gallien et al. (2009) identified 946 proteins in *M. smegmatis*, characterizing 443 N-terminal peptides, and revealed an error rate of 19% in predicted TSSs. Kelkar et al. (2011) reported 41 novel protein-coding genes in *Mycobacterium tuberculosis* strain H37Rv. By identifying N-terminal peptides, the authors were able to correct the TSS of 33 proteins, and validate the TSS of 727 annotated proteins. Strikingly, the authors identified eight proteins with evidence for translation initiation at two different sites.

The occurrence of false positive peptide identifications from tandem mass spectrometry data implies the need for statistical concepts such as the false discovery rate (FDR) and posterior error probability (PEP) score to gauge the reliability of identifications. The FDR is controlled by scoring and ranking peptide spectral matches (PSMs), and identifying the score above which a maximum allowable proportion of PSMs from a decoy database are identified—indicating the expected maximum proportion of incorrect PSMs—while posterior error probability (PEP) indicates the probability of error of a single PSM (Käll et al., 2008). The *MaxQuant* algorithms calculate PEP scores based on search engine PSM scores and peptide length, and determine a cutoff PEP score at a specific FDR (Cox and Mann, 2008).

Krug et al. (2013) compared the number and median PEP scores of novel peptide identifications with reverse sequence hit peptides in a proteogenomic analysis of *Escherichia coli*, and determined that the absolute number as well as the median PEP-values for both groups were very similar. The authors concluded that as a genome approaches complete annotation, the likelihood of any novel peptide identification being a false positive identification increases (Krug et al., 2013).

Annotation of coding regions for organisms is a continuously evolving process. The genome of *M. tuberculosis* was sequenced in 1998 (Cole et al., 1998), yet a few years later, the same authors re-annotated the genome and identified 71 more ORFs than before (Camus et al., 2002). To date, the *M. tuberculosis* genome is annotated with 4018 ORFs^1^. This highlights the importance of continuous review and re-annotation of genomes as technologies improve. The *M. smegmatis* mc^2^155 genome may not yet be fully annotated, and the genome sequence has been shown to contain multiple errors (Deshayes et al., 2007), one of the reasons being the high GC content and genome annotation shortfalls, such as short protein validation and incorrect TSS assignment of genes. That poses a problem by limiting our understanding of the many cellular processes coded by these genes. It is imperative that the genome of *M. smegmatis* mc^2^155 be fully annotated since this bacterium, due to its non-pathogenic and fast growing status, is used frequently as a model organism to study the biology of *M. tuberculosis*, the causative agent of Tuberculosis. Tuberculosis continues to be a burden on the health system, with an estimated 9.6 million cases of infection and 1.5 million deaths in 2014, despite a globally decreasing incidence of ~1.5% every year since 2000 (WHO, 2015). Limited understanding of the biology of *M. tuberculosis* is an obstacle to improving current treatment and eradication of the disease. Thus, it is hoped that refinements in proteogenomics pipelines and the study of model organisms of *M. tuberculosis* such as *M. smegmatis* mc^2^155, may further our understanding of this pathogenic organism.

In this study, we developed and applied a compacted six frame genomic database, to map high resolution and high accuracy tandem mass spectrometry coupled to liquid chromatography data to re-evaluate the genome annotation of *M. smegmatis* mc^2^155. We also used a database of *ab initio* gene predictions using *GeneMarkS*^2^ in the proteogenomics pipeline to identify novel open reading frames, gene model validations, and gene model modifications. By analyzing the PEP score distribution of novel, annotated, and reverse sequence hit peptides, we explored the current annotation status of *M. smegmatis* mc^2^155.

## Materials and methods

### Bacterial cultures

Wild type strain of *M. smegmatis* mc^2^155 were grown in 7H9 Middlebrook (BD, Maryland, USA) broth supplemented with 0.05% Tween 80, OADC (Becton Dickinson), and 0.2% glycerol (v/v). Cells were grown at 37°C with continuous agitation (120 rpm).

### Protein extraction

Cells were harvested from three biological replicates each during the exponential and early stationary phase (OD_600_ ~ 1.2 and 1.8, respectively) by centrifugation at 4000 g for 15 min at 4°C. Cell pellets were washed with PBS (10 mM phosphate buffer, 2.7 mM potassium chloride, and 137 mM sodium chloride, pH 7.4) and flash frozen in liquid nitrogen and stored at −80°C. Pellets were suspended in lysis buffer [500 mM Tris-HCl, 0.1% (w/v) SDS, 0.15% sodium deoxycholate], 1 × protease inhibitor cocktail, 1 × phosphatase inhibitor cocktail (Roche, Mannheim Germany), and 50 μg/ml lysozyme (Repaske, 1956) and disrupted by sonication at maximum power for six cycles of 30 s, with 1 min cooling on ice between cycles (Rezwan et al., 2007). Lysates were further clarified using centrifugation at 4000 g for 5 min and filtering through a 20 μm pore size low-protein binding filter (Merck, NJ, USA). Proteins were precipitated using the chloroform–methanol precipitation method as previously described (Wessel and Flügge, 1984). Protein precipitate was suspended in denaturing buffer (10 mM Tris-HCl, 6 M urea, 2 M thiourea, pH 8). Protein concentration was determined using the modified Bradford assay as described by Ramagli (1999).

### In-solution trypsin digestion

Fifty micrograms of precipitated protein was reduced with 1 mM DTT for 1 h followed by another hour of incubation in the presence of 5.5 mM IAA. Alkylated protein samples were pre-digested for 3 h at room temperature with lysyl endopeptidase LysC (Waco, Neuss, Germany). Pre-digested samples were diluted four-fold with 20 mM ammonium bicarbonate pH 8 prior to trypsination. Sequencing grade modified trypsin (Promega, Madison, USA) was used at a protease:protein ratio of 1:50 (w/w) for 14 h at room temperature with gentle agitation. Trypsination was terminated with 0.1% formic acid final concentration (Sigma Aldrich, St Louis, USA). Ten micrograms of peptides were desalted using a homemade stage tip containing Empore Octadecyl C18 solid-phase extraction disk (Supelco; Rappsilber et al., 2003). Activation, equilibration, and peptide wash and elution were all carried out using centrifugation at 5000 g for 5 min. Activation and equilibration of the C18 disk was carried out using three rinses with 80% acetonitrile (ACN), followed by three rinses with 2% ACN, respectively. Peptide rich solution was loaded onto the disk and centrifuged. Desalting was carried out using three washes of 2% ACN, followed by three washes of 2% ACN containing 0.1% formic acid (Sigma). Elution of desalted peptides into glass capillary tubes was carried out using three rounds of 100 μL of 60% ACN, 0.1% formic acid. Peptides were dried in a vacuum and resuspended in 2% ACN, 0.1% formic acid at 50 ng/μL.

### LC/MS/MS analysis

Data acquisition was performed on the Orbitrap Q-Exactive mass spectrometer (Thermo Scientific) in a data-dependent manner, coupled to the Dionex Ultimate 3000 UHPLC (Thermo Scientific). One microgram of peptides were loaded on to an inhouse packed pre-column (100 μm ID × 20 mm) connected to an in-house packed analytical column (75 μm × 400 mm) both packed with C18 Luna 5 μm 100 Å beads (04A-5452) for liquid chromatography separation. The flow rate was set to 300 nl/min with the gradient of 2% to 25% ACN for 125 min, then up to 35% in 5 min. To wash the column ACN was increased to 80% for 20 min followed by a column equilibration at 2% ACN for 10 min. A top 10 method with 30 s dynamic exclusion was used to acquire mass spectra with automatic switching between MS and MS/MS scans. The LC/MS/MS methods used have been described previously (Nakedi et al., 2015).

### Proteogenomic databases

The genome of *M. smegmatis* mc^2^155 was accessed from the *European Nucleotide Archive* under the accession number CP000480, Genome Assembly GCA_000015005.1 (Fleischmann et al., 2006). To facilitate genomic database compaction each open reading frame in the six frames was translated *in silico* starting at the most upstream start codon—using possible start codons ATG, GTG, and TTG—and sequences below a minimum translated length of 20 amino acids were excluded from the database. For sequences at the end of each of the genomic frames that did not end in a stop codon, the sequence up till the last in-frame codon was translated and included in the database. The genomic coordinates of the six frame translated sequences were included in the *FASTA* headers of the database. Translated sequences that occurred more than once in the database were combined into a single entry with multiple genomic coordinates. The Reference proteome for *M. smegmatis* mc^2^155 was obtained from UniProt^3^ (Proteome ID UP000000757), and any translated six frame sequence overlapping with or identical to a Reference protein sequence was mapped to that protein in the *FASTA* header. Where a Reference sequence was located downstream of a translated six frame TSS, the number of amino acids difference in the N-termini of the two sequences was recorded, and six frame sequences identical to a protein in the Reference proteome were labeled. Overlapping open reading frames were included in the database, leading to a final database size of 79,481 entries.

A second genomic database for targeted identification of TSSs was generated using *ab initio* prediction of protein-coding genes with the *GeneMarkS* software package (version 4.28)—at the same time providing supporting evidence for novel protein identifications and gene model modifications. This software uses a Hidden Markov Model algorithm, combined with models of protein coding and non-coding regions and gene regulation sites, to predict the occurrence of genes in a DNA sequence (Besemer et al., 2001). The predicted genes were translated and the final database contained 6655 sequences.

### Database search using maxquant

The *MaxQuant* software package (version 1.5.0.30) was used to search the raw MS spectra using the Andromeda search engine separately against the six frame database, UniProt Reference proteome and *GeneMarkS* database—using reverse decoy databases and a selection of known contaminants provided by *MaxQuant*. Trypsin and LysC were selected as enzymes, and a maximum of three missed cleavages were allowed. Specific enzyme mode was selected, which allowed for the detection of non-tryptic N-terminal peptides of database entries, peptides with N-Met cleavage, and fully tryptic peptides. Carbamidomethyl was set as a fixed modification and Acetyl (Protein N-term) and Oxidation (M) were set as variable modifications. A minimum peptide length of seven amino acids, and a minimum of one unique peptide identification per protein group, was required. The default *MaxQuant* false discovery rate (FDR) cutoff of 0.01 (1%) was used at the PSM, peptide and protein group levels. The mass spectrometry proteomics data have been deposited to the ProteomeXchange Consortium (Vizcaíno et al., 2014) via the *PRIDE* partner repository (Vizcaíno et al., 2013) with the dataset identifier PXD003500 and the data is freely available^4^.

### Proteogenomic analysis

We relied heavily on previously published proteogenomic protocols, in particular the methodology described by Kelkar et al. (2011)—differing in that we used a separate search database (*GeneMarkS*) for targeted TSS identification, and did not exclude all novel peptides mapping to multiple genomic locations, allowing for the identification of paralogous translated ORF sequences.

All *MaxQuant* results for the different databases were combined using an in-house python script. Use was made of the *Biopython* software package^5^ (Cock et al., 2009). Pettersen et al. (2015) only considered proteins identified in at least two replicates in their proteogenomic analysis of enterotoxigenic *Escherichia coli*—similarly, we only considered peptides identified in at least two replicates for further analysis. All peptides were searched against the genome dynamically translated in the six reading frames, and peptides unique to a single position in the genome were identified. Mycobacteria are known to have duplications in protein-coding genes due to the effect of transposable elements (Dale, 1995). To allow for the detection of paralogous sequences, peptides specific to a single repeating translated six frame ORF sequence in the genome were identified as paralogous sequence peptides, and also included in the analysis, but identified as belonging to paralogous translated ORF sequences.

**Figure 1:**
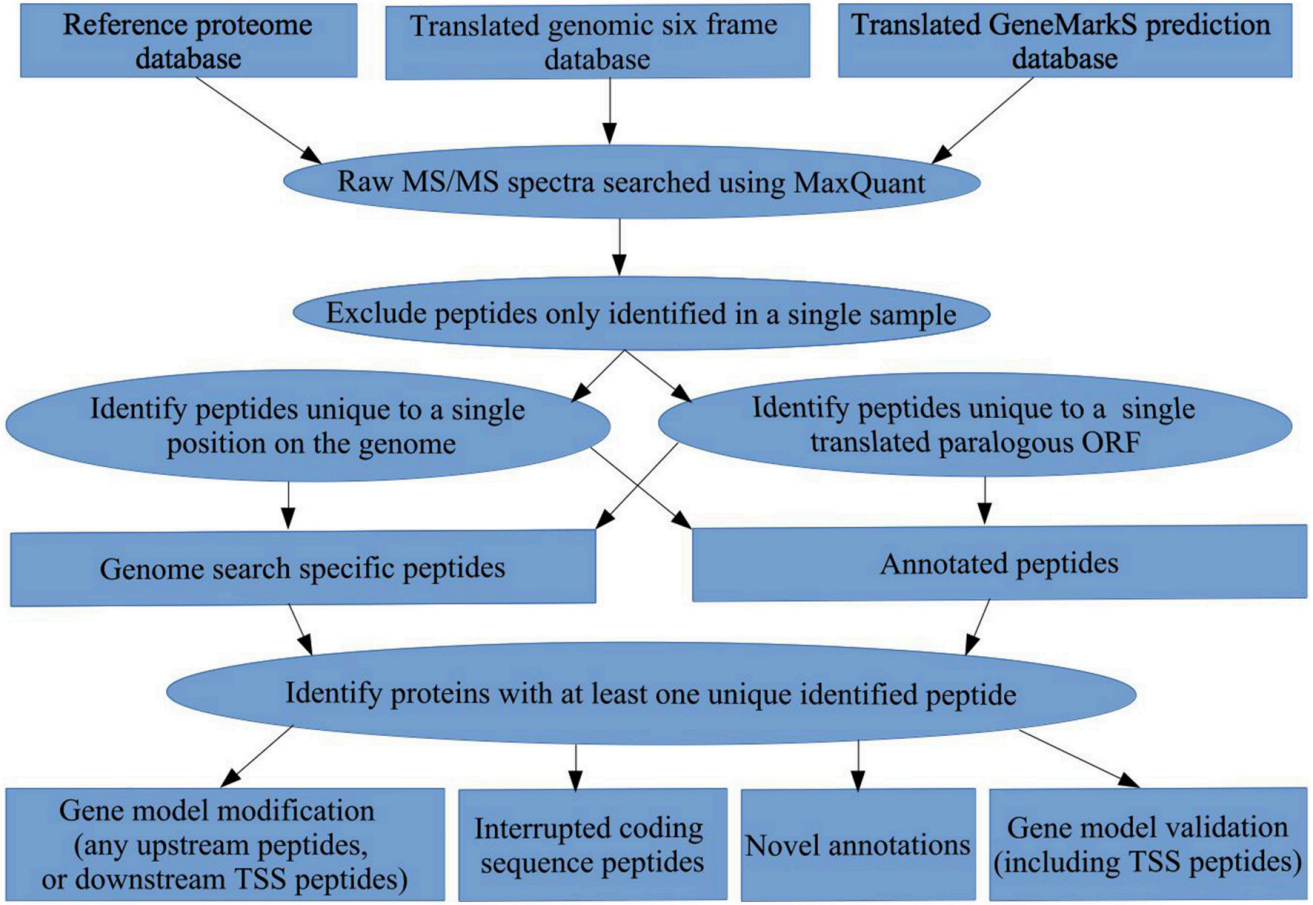
**Bioinformatics workflow**. A summary of the proteogenomic pipeline applied to obtain gene model validations, gene model modifications, novel ORF annotations, and interrupted coding sequence identifications in *M. smegmatis* mc^2^155, using a translated genomic six frame database, a translated *GeneMarkS* gene prediction database, and the Reference proteome.

Peptides not found in the Reference proteome were identified as genome search specific peptides (GSSPs). GSSPs are peptides mapping to genomic regions not considered to be coding regions, or not included in overlapping or adjacent gene models (Kelkar et al., 2011). We used the UniProt Reference proteome as a benchmark for annotation status to discriminate annotated peptides from GSSPs. Only GSSPs were used for upstream gene model modifications and novel gene model identifications, while N-terminal peptides mapping downstream in the gene model of Reference proteins were used for downstream TSS assignment. N-terminal peptides mapping to the start of Reference protein gene models were used for TSS validation. All protein groups with no peptides unique to the protein sequence after performing the genome search, were excluded from further analysis. All results in the different databases mapping to the same ORF were identified for comparison, and the unique identified peptide set across all three databases for each ORF was obtained. All database entries were mapped to their respective ORFs in the genome using an in-house python script (see Figure 1).

TSS peptides were identified by the non-tryptic nature of their N-terminals, *N-Met* cleavage, or N-terminal acetylation. Reference protein start sites were validated by identifying TSS peptides mapping to the genomic coordinates of the start of a Reference protein sequence. All open reading frames mapping to Reference proteins, with peptides identified upstream in the ORF from the annotated TSS of the Reference protein, were identified for upstream gene model modification, while N-terminal peptides mapping downstream of a Reference protein start were used for downstream gene model modification. The coordinates of novel ORFs were compared to the coordinates of known cDNA features (sequenced transcripts obtained from Ensembl^6^ using a sense strand genome coordinate search (in-house python script), and ORFs overlapping with cDNA evidence in the genome were identified. A nucleotide BLAST of novel ORFs was performed against the transcript sequences, requiring same strand alignment with an *E*-value cutoff of 0.0001. A protein BLAST of novel translated ORFs, as well as ORFs for which gene model modification were identified, was performed against the NCBI non-redundant (nr) BLAST database, and the highest scoring alignment by *E*-value was obtained for each sequence, using an *E*-value cutoff of 0.0001. The leading gi IDs of protein BLAST results were converted to UniProtKB IDs using the UniProt Retrieve/ID Mapping tool. Novel ORFs were ranked by number of identified GSSPs, protein BLAST evidence, cDNA nucleotide BLAST evidence, and cDNA coordinate overlap.

Interrupted CoDing Sequences (ICDSs) are erroneously shortened gene model predictions either due to the presence of unrecognized true genomic events (such as programmed frameshifts or in-frame stop codons), or artificially due to sequencing errors (Perrodou et al., 2006). We followed the methodology described by Perrodou et al. (2006) and Deshayes et al. (2007) to identify ICDSs based on shared homologous sequence evidence (protein BLAST) of adjacent or overlapping non-paralogous ORFs, but differ in that we only focused on ORFs identified at the peptide level.

All peptides identified in the three databases in at least two replicates, as well as the *GeneMarkS FASTA* database, were processed into general feature format (GFF) files for visualization using the Ensembl genome browser for this strain^7^—allowing for manual examination of novel annotations, gene model modifications, and ICDSs (see Supplementary Data Sheets 1–4).

The PEP score distributions of novel, annotated and reverse peptide identifications from each database were analyzed with in-house python and R scripts. Use was made of the python module *matplotlib* (Hunter, 2007) to produce boxplots of the PEP score distributions, while the R *ggplot2* package (Wickham, 2009), as well as the *density* and *qqnorm* functions from the R *stats* package (R Core Team, 2015) were used to plot the PEP score distributions and investigate for normality. As non-normal PEP score distributions were found, the non-parametric Kruskal–Wallis analysis of variance test was chosen to investigate for differences between the groups—using the *kruskal.test* function from the R *stats* package, followed by *post-hoc* analysis with a two-sided Dunn test (Dunn, 1964) with Bonferroni correction using the *dunnTest* function from the R *FSA* package (Ogle, 2016). Venn diagrams were produced using the online venn diagram plotting tool Venny 2.1.0 (Oliveros, 2016).

## Results

**Figure 2:**
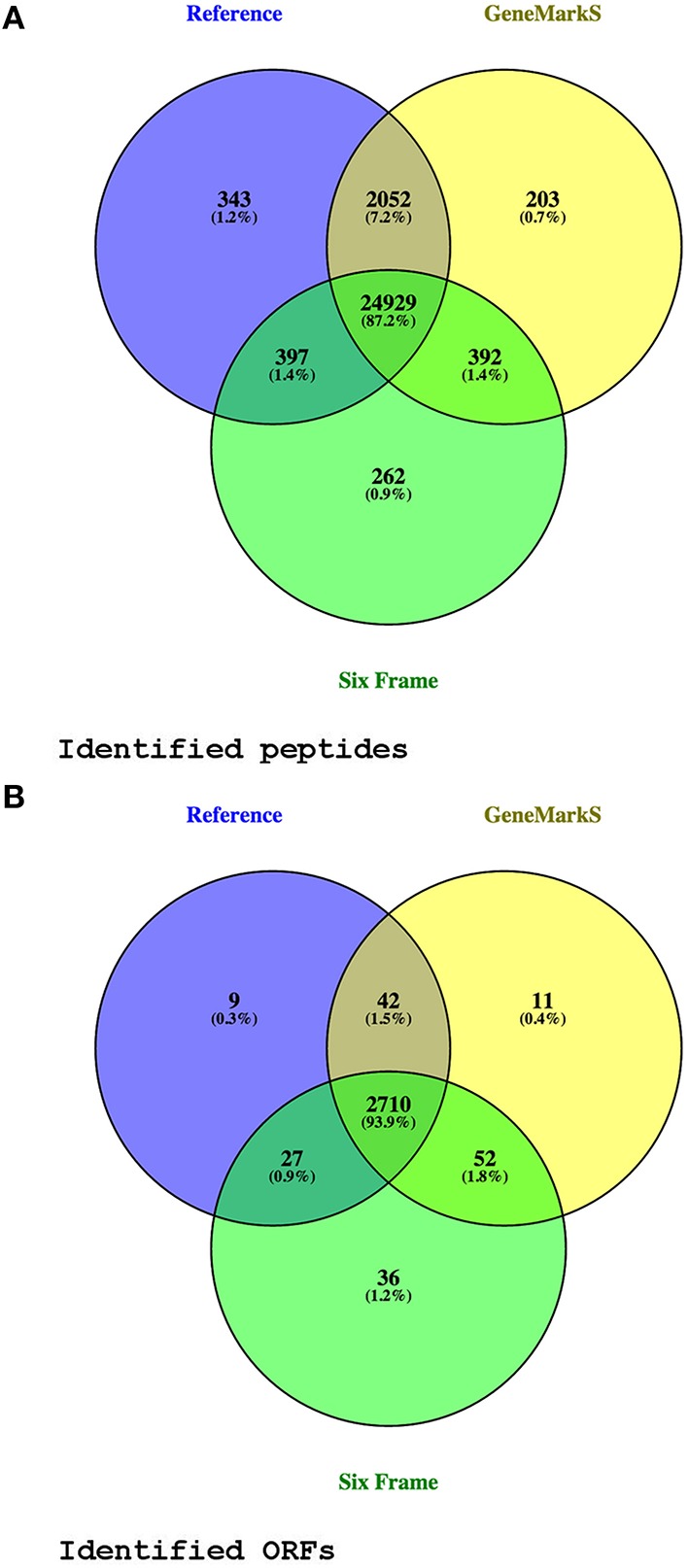
**Peptide and ORF identification venn diagrams**. Panel **(A)** compares peptide identifications from three different database searches. Panel **(B)** shows the genomic open reading frames (ORFs) with at least one unique peptide identified, from the different databases searches. Only peptides identified in more than one replicate were considered. Two identified Reference proteins spanning multiple ORFs (A4ZHT6 and A4ZHR8), not mapping on a one to one basis to a genomic ORF, are included in the diagram as separate ORFs.

From the 276,472 MS/MS spectra submitted to *MaxQuant* at 1% FDR, 26,125 peptide sequences were identified from 172,570 spectra using the translated six frame database, 27,895 peptides from 176,518 spectra using the Reference proteome database, and 27,735 peptides from 176,301 spectra using the *GeneMarkS* database. Only protein groups were considered where the leading protein had at least one unique peptide identification. By mapping identified proteins from the different databases to their corresponding ORFs in the genome, 2887 ORFs were identified at the peptide level (identical translated ORF sequences with multiple occurrences in the genome having been combined into a single entry with multiple genomic coordinates at the database generation phase; see Supplementary Table 1 and Figure 2).

### TSS peptide identifications

TSS peptide identifications can be divided into peptides with and without *N-Met* cleavage. We identified 137 TSS peptides with non-tryptic *Met* N-termini at a genomic start codon position (of which three were acetylated at the N-terminal), and 549 *N-Me*t cleaved peptides (of which 127 were acetylated at the N-terminal). The distribution of penultimate amino acids of identified *N-Met* cleaved peptides—*Thr* (188), *Ser* (134), *Ala* (128), *Pro* (51), *Gly* (18), *Val* (15), *Asn* (13), *Leu* (1), and *Arg* (1)—corresponds to the non-random nature of *Met*-AP cleavage (Link et al., 1997; Frottin et al., 2006). Further, the identified start codon distribution of all non-paralogous ORFs with an identified N-terminal—ATG 63.96%, TTG 1.14%, and GTG 34.90%—corresponds to the high percentage of GTG start codons reported previously in *M. tuberculosis* (Cole et al., 1998) and *M. smegmatis* (Gallien et al., 2009), but with a lower proportion of identified TTG start codons. We identified three ORFs with evidence for multiple initiation, corresponding to the observations of Kelkar et al. (2011; see Supplementary Table 2).

Two ORFs were detected where the most upstream evidence was an *N-Met* cleaved peptide and the penultimate position corresponded to another possible start codon. For one of these proteins, the second amino acid in the peptide was also a *Met* located at an ATG codon, thus not allowing for discrimination between N-Met cleavage and downstream initiation. For the second protein, the N-terminal was identified by an *N-Met* cleaved peptide with V as the penultimate amino acid, mapping to a GTG codon at the annotated start site of a Reference proteome sequence. Due to the known initiation of translation with *fMet*, this was concluded to be an instance of *N-Met* cleavage, leading to modification of the gene model to include the adjacent upstream start codon.

### Gene model validations

**Figure 3:**
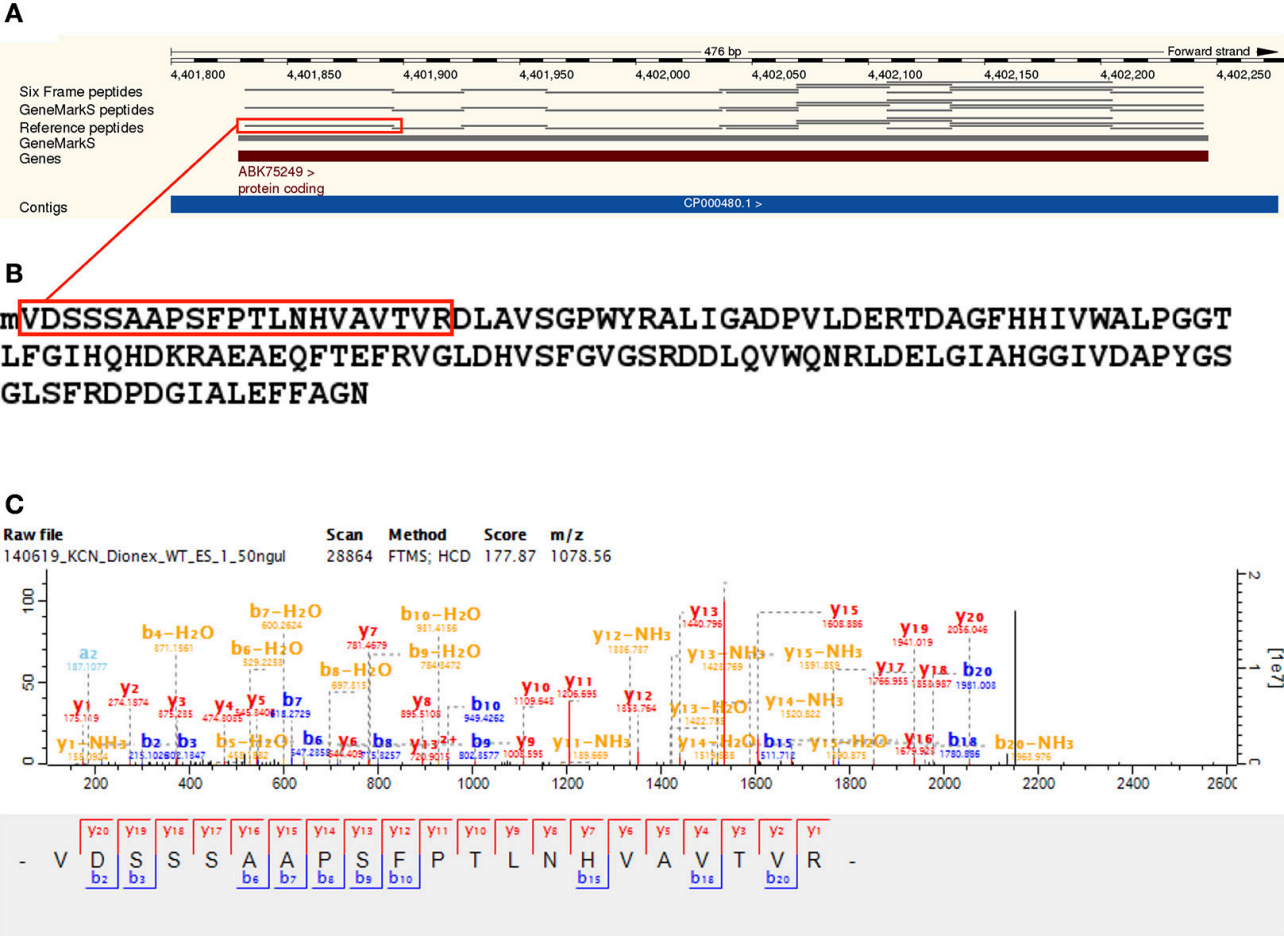
**Reference protein gene model validation** —**A0R0A1**. A validation of the gene model of the Reference protein A0R0A1 (Glyoxalase/bleomycin resistance protein/dioxygenase). This is an unreviewed predicted entry associated with dioxygenase activity (GO molecular function), of length of 138. Panel **(A)** illustrates validation of the gene model at the peptide level visualized on the Ensembl genome browser, with a sequence coverage of 99.28% from 13 peptides and a total of 152 MS/MS spectra identified in the Reference proteome database search. Panel **(B)** shows the identified translation start site (TSS) peptide, illustrating the cleavage of initiator methionine (N-Met) by methionine aminopeptidase (MAP) with Val as the penultimate amino acid. Panel **(C)** shows a representative MS/MS spectrum for the identified TSS peptide.

We identified 2810 genomic ORFs annotated to Reference proteins with at least one unique peptide—with a median of six unique peptides identified per ORF. Using N-terminal peptide evidence, we validated the TSS for 558 Reference protein gene models. The start codon distribution of validated Reference protein TSSs also reflected the higher proportion of GTG start codons in Mycobacteria—with 65.05% ATG, 0.72% TTG, and 34.23% GTG. Prominent Reference proteins identified include A0R1H7_MYCS2—Fatty acid synthase—with 168 unique peptides and 71.32% sequence coverage, RPOC_MYCS2—DNA-directed RNA polymerase subunit beta—with 96 unique peptides and 79.5% sequence coverage, Q3L891_MYCS2—Linear gramicidin synthetase subunit D, predicted protein—with 89 unique peptides and 54.98% sequence coverage, Q3L885_MYCS2—Type I modular polyketide synthase, predicted protein—with 88 unique peptides and 38.44% sequence coverage, and A0R617_MYCS2—Polyketide synthase, predicted protein—with 82 unique peptides and 62.14% sequence coverage. The predicted and unreviewed Reference proteome entry A0R0A1_MYCS2 (Glyoxalase/bleomycin resistance protein/dioxygenase) was identified with 99.28% sequence coverage from 13 identified peptides and 152 MS/MS spectra (see Figure 3 and Supplementary Table 3).

**Figure 4:**
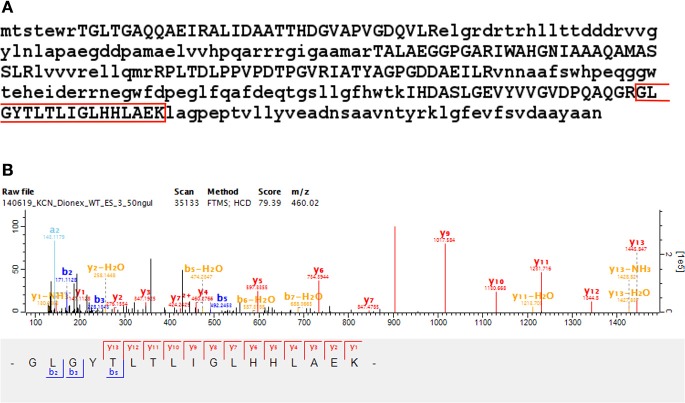
**Reference protein frameshift validation** —**MSHD**. Shows the identification of A4ZHT6 (Mycothiol acetyltransferase), a Reference protein inferred from homology. **(A)** This sequence corresponds to an interrupted coding sequence (ICDS) identified by Deshayes et al. (2007), which they confirmed by resequencing to span a sequencing error in the *M. smegmatis* mc^2^155 genome (GenBank accession DQ866865). We present peptide evidence for this protein with eight peptides identified from 50 MS/MS scans after searching the Reference proteome database, as well as identifying a peptide spanning the frameshift position (245) in the corrected sequence—highlighted in red. **(B)** A representative spectrum of the highlighted peptide, which was identified with four MS/MS scans, supporting the sequence correction at the peptide level.

Gene model validation was also obtained for the Reference protein MSHD_MYCS2 (Mycothiol acetyltransferase, inferred from homology). This is an interrupted coding sequence (ICDS) identified by Deshayes et al. (2007)—which they confirmed by resequencing to span a sequencing error in the *M. smegmatis* mc^2^155 genome (*GenBank* accession DQ866865). We present first-time peptide evidence for this protein with eight peptides identified from 50 MS/MS scans using the Reference proteome database, including a peptide spanning the frameshift position (245) in the corrected sequence (see Figure 4 and Supplementary Table 4).

### Upstream gene model modifications

**Figure 5:**
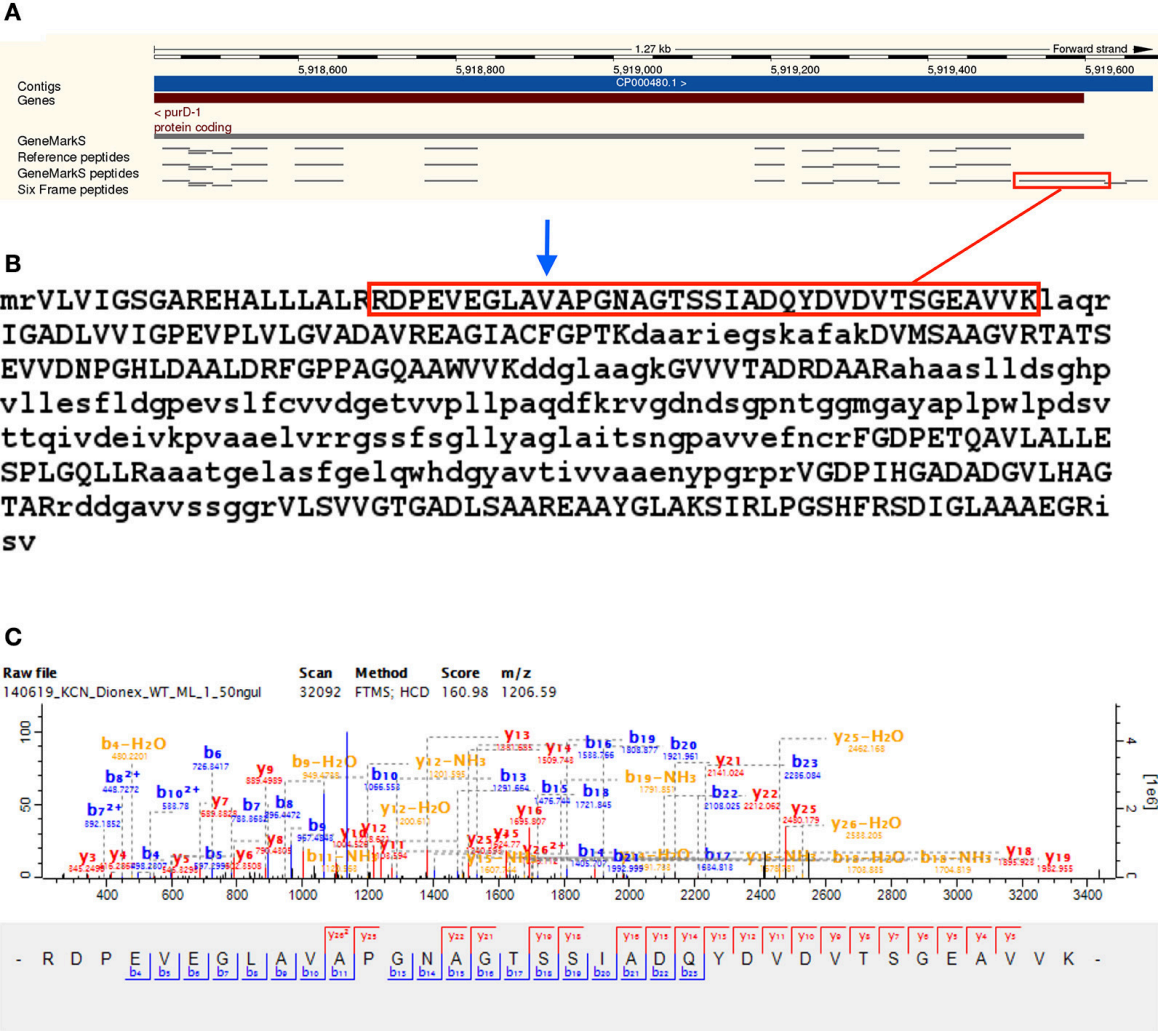
**Upstream gene model modification** —**A0R4J1**. Shows evidence for gene model modification of the Reference protein A0R4J1 (Phosphoribosylamine–glycine ligase) by N-terminal extension, with the N-terminal of three identified peptides extending upstream of the predicted TSS, extending the N-terminal of the protein by 29 amino acids. The modified sequence is identical to the predicted protein I7GFT2 for the same strain (not included in the Reference proteome). Panel **(A)** shows the peptide RDPEVEGLAVAPGNAGTSSIADQYDVDVTSGEAVVK—highlighted in red—extending upstream of the annotated TSS along with two other peptides. Panel **(B)** shows the sequence of the modified gene model of A0R4J1, with the annotated TSS position indicated by the blue arrow. Panel **(C)** shows a representative MS/MS spectrum of RDPEVEGLAVAPGNAGTSSIADQYDVDVTSGEAVVK, identified from eight MS/MS spectra in the translated six frame database search.

Upstream peptide evidence was identified for 81 Reference proteome gene models. Upstream peptide identifications can be grouped into N-terminal (TSS) or fully tryptic upstream peptides—for 39 of the upstream gene model modifications the TSS was detected exactly, while for the 42 sequences with only non-tryptic upstream peptides, the next upstream start-codon in the sequence was located. The putative new gene models thus obtained were searched against the NCBI nr database using protein BLAST, to identify orthologous gene models, or alternative gene models in the same strain. For 58 of the modified gene models, nr BLAST alignment yielded a sequence of the same length, supporting the gene model modification, while 14 nr BLAST alignments in the group without exact TSS identification indicated a TSS further upstream. Only one nr BLAST result in the group with an identified TSS indicated a further upstream TSS than the one identified. The start codon GTG was overrepresented in upstream TSS identifications, making up 38.46%—supporting the observation that ATG may be over predicted as translational start codon (Gallien et al., 2009). The median length of upstream N-terminal extension was five amino acids in the group with upstream TSS identifications. The Reference protein A0R4J1_MYCS2 (Phosphoribosylamine–glycine ligase, inferred from homology) was identified with the N-terminal of three identified peptides extending upstream of the predicted TSS—extending the N-terminal of the protein by 29 amino acids. The modified sequence is identical to the predicted protein I7GFT2_MYCS2 of the same strain (not included in the Reference proteome; see Figure 5 and Supplementary Table 5).

### Downstream gene model modifications

**Figure 6:**
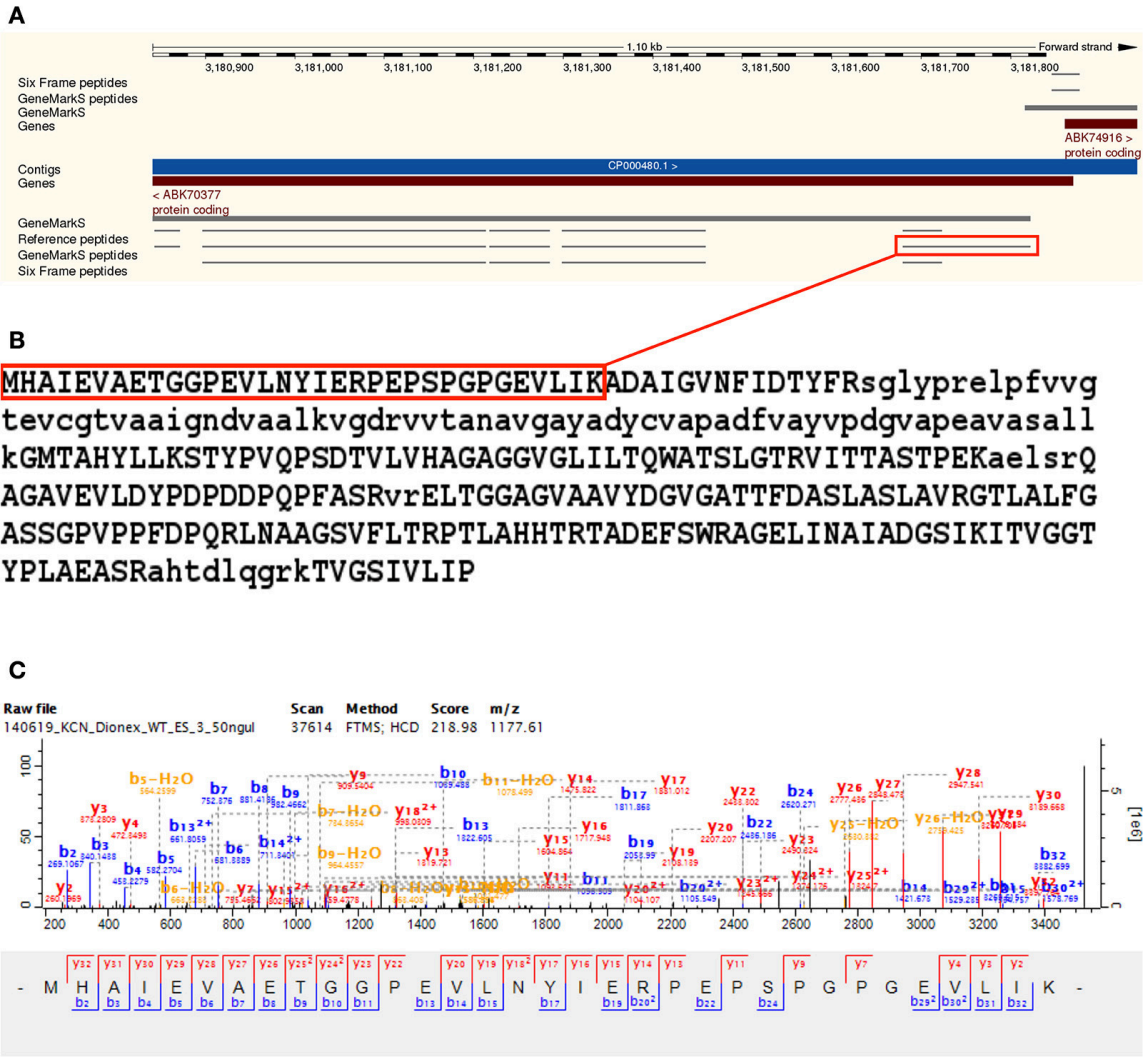
**Downstream gene model modification** —**A0QWY3**. Illustrates the downstream modification of an annotated predicted gene model A0QWY3 by 16 amino acids, with the identification of a TSS peptide MHAIEVAETGGPEVLNYIERPEPSPGPGEVLIK with a non-tryptic N-terminal downstream of the annotated TSS. In this case, the downstream TSS peptide corresponded to the N-terminal of the GeneMarkS predicted sequence for this ORF, thus allowing this semi-tryptic TSS peptide to be included in the GeneMarkS database search space. The modified sequence is identical to I7G8G0, predicted for the same strain but not included in the Reference proteome. Panel **(A)** visualizes the identified peptides using the Ensembl genome browser, with the downstream TSS peptide highlighted in red. The downstream gene model modification of A0QWY3 corresponds with the simultaneous upstream gene model modification of A0QWY4 on the opposite strand, supporting both reannotations. Panel **(B)** illustrates the modified gene model, with the TSS peptide highlighted in red. Panel **(C)** illustrates a representative MS/MS spectrum for the downstream TSS peptide, which was identified from 34 MS/MS scans using the *GeneMarkS* database search.

Downstream TSS evidence was identified for 24 Reference proteome gene models—with all cases corresponding to an alternative TSS prediction in the *GeneMarkS* database. The predicted gene model A0QWY3_MYCS2 (Quinone oxidoreductase) was shortened by 16 amino acids, with the identification of a TSS peptide MHAIEVAETGGPEVLNYIER PEPSPGPGEVLIK with a non-tryptic N-terminal downstream of the annotated TSS. The downstream TSS peptide corresponded to the N-terminal of the *GeneMarkS* predicted sequence for this ORF, thus allowing this semi-tryptic TSS peptide to be included in the *GeneMarkS* database search space. The modified sequence is identical to I7G8G0_MYCS2, predicted for the same strain but not included in the Reference proteome. The downstream TSS peptide was identified from 34 MS/MS scans (see Figure 6 and Supplementary Table 6).

### Novel ORF identifications

Due to the high prevalence of gene prediction errors, inclusion in the UniProt Reference proteome was used as a benchmark for annotation status in this study. Thus, all identified ORFs not annotated to a Reference proteome entry were considered novel identifications. Peptide evidence mapping to ORFs not annotated to a Reference proteome entry was identified for 72 ORFs, of which 44 were identified with two or more peptides. ORFs with one or more identified peptides that occurred adjacent to another novel ORF identification, were examined as possible evidence for Interrupted CoDing Sequences (ICDSs). Nineteen novel ORFs that were identified with a single peptide, and were supported by either protein BLAST alignment or a previously identified transcript overlapping on the genome on the same strand, are presented as lower ranking evidence for genome annotation. Nine ORFs with only one identified peptide and no supporting evidence, were excluded from the further analysis due to the high likelihood of these identifications being erroneous—leading to a total of 63 novel ORF identifications (see Supplementary Table 7).

#### Validation of previously identification interrupted coding sequences (ICDSs)

Twelve non-Reference proteome ORFs identified at the peptide level corresponded to six interrupted coding sequences previously reported by Deshayes et al. (2007), with GenBank accessions DQ866867, DQ866856, DQ866859, DQ866863, DQ866858, and DQ866873—see Supplementary Figures 1A–E,G, respectively, for Ensembl genome browser visualizations, and Supplementary Table 8 rows 2–11 and 14–15 for detailed information. The above authors had shown by resequencing that these frameshifts corresponded to genome sequencing errors, and they also reported peptide-level evidence for two of these sequences using nano-LC/MS/MS analysis (DQ866873 and DQ866856). Thus, we were able to identify four of these ICDSs with first-time peptide evidence, and validate two ICDSs previously identified at the peptide level.

#### Novel ICDSs

Four likely novel ICDS sequences were identified with peptide evidence spanning either side of a possible genomic frameshift region from eight non-Reference proteome ORFs. In an interesting case, three novel peptides were identified from an ORF with the closest nr protein BLAST alignment to a predicted protein in *Mycobacterium goodii*—A0A0K0X632_9MYCO (Peptidase M75). A non-Reference proteome predicted ORF I7FS93_MYCS2 was identified partially overlapping and upstream of this ORF with peptide evidence. This upstream ORF also aligned with high confidence to the same sequence in *M. goodii*. To our knowledge an ICDS has not previously been identified at this position, but peptide evidence from two overlapping reading frames and alignment to an orthologous sequence of a related species, supports the identification of a novel ICDS at this site—although the existence of separate adjacent protein coding genes cannot entirely be excluded (see Supplementary Figures 1F and Supplementary Table 8 rows 12–13).

Another novel ORF with only one identified peptide—TAILDAAAQLIAER—was identified upstream and partially overlapping the predicted ORF I7G4B8_MYCS2 that was identified with three peptides (see Supplementary Figure 1J and Supplementary Table 8 rows 20–21). Both ORFs aligned with high confidence to the predicted protein L8F9F0_MYCSM of *M. smegmatis* MKD8—the genome sequence of which has recently been announced (Gray et al., 2013). Thus, orthologous sequence evidence combined with evidence at the peptide level strongly supports the existence of an ICDS in this position, and further investigation is needed to ascertain whether this is an occurrence of authentic mutation or sequencing error, or in fact two separate protein-coding genes. The identification of two more ICDS sequences with peptide evidence on either side of a possible frameshift position was also facilitated by alignment to orthologous sequences submitted by Gray et al. (2013) as a result of their genome sequencing efforts of *M. smegmatis* MKD8—emphasizing the iterative nature of genome annotation as new data becomes available (see Supplementary Figures 1H,I, and Supplementary Table 8 rows 16–19).

#### Novel ORFs identified with two or more peptides

**Figure 7:**
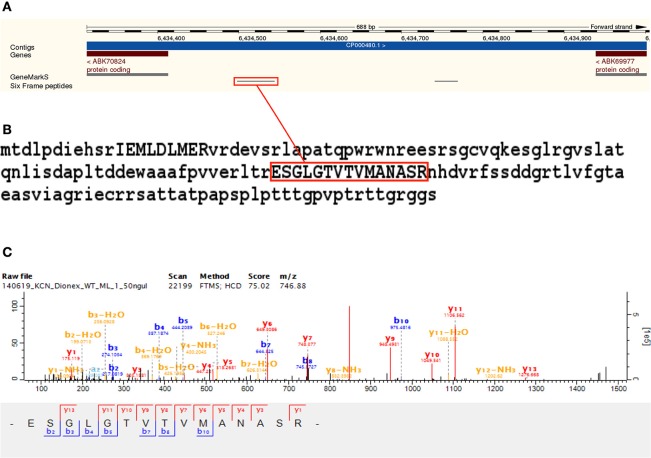
**Novel ORF identification**. Panel **(A)** shows the identification of two peptides from eight MS/MS spectra mapping to an intergenic region in *M. smegmatis* mc^2^155 from position 6,434,313 to 6,434,801 on the reverse strand. Panel **(B)** shows the identified sequence, extended up to the next upstream start codon, with identified peptides in uppercase. The sequence yielded a protein nr BLAST alignment to G7CE94 (Lipoprotein LppV), a 182 amino acids long predicted protein annotated to *M. thermoresistibile*, with an *E*-value of 2.13747e-44. Panel **(C)** shows a representative MS/MS spectrum identified with the translated six frame database.

We identified 44 non-Reference proteome ORFs with two or more peptides (with a median of five identified unique peptides per ORF in this group). Protein BLAST alignments were obtained for 43 of these, and the gi accession numbers thus obtained were mapped to their corresponding entries in UniProt using the UniProt “Retrieve/ID mapping” tool^8^. Of the 39 sequences that were mapped with this tool, 31 were predicted and eight were inferred from homology. Three of these sequences were annotated to *M. smegmatis* MKD8, one to *M. thermoresistibile* strain ATCC 19527, and four to *M. smegmatis* non-specifically. One identified ORF alignment—discussed above as part of an identified ICDS—aligned to a sequence annotated to *M. goodii*. The remaining 30 identified ORFs aligned to Non-Reference proteome sequences from *M. smegmatis* mc^2^155—with 26 predicted and four inferred from homology. An interesting novel ORF was identified with two peptides from eight MS/MS spectra mapping to an intergenic region with a genomic position from 6,434,313 to 6,434,801 on the reverse strand. The sequence yielded a protein nr BLAST alignment to G7CE94_MYCTH (Lipoprotein LppV), a 182 amino acids long predicted protein annotated to *M. thermoresistibile*, with an *E*-value of 2.13747e-44 (see Figure 7 and Supplementary Table 7).

### Database comparison

The PEP scores of the identified peptides were obtained from the *MaxQuant* peptides.txt file—which contains the identified peptides with the PEP score calculated using the peptide length and the *Andromeda* score for the best associated MS/MS spectrum. Density plots of the distribution of PEP scores for each database revealed non-gaussian PEP score distributions. Analysis of variance of novel, annotated and reverse peptide PEP scores for each database was performed using the Kruskal–Wallis non-parametric analysis of variance test followed by *post-hoc* analysis with a two-sided Dunn's test and Bonferroni correction (see Supplementary Data Sheet 5). A statistically significant difference between group means (annotated, novel and reverse peptides) for each of the three databases was found (*p*-values < 2.2e-16). *Post-hoc* analysis indicated a significant difference between reverse and annotated peptide group PEP scores for both the *GeneMarkS* (adjusted *p*-value 6.68e-21), six frame database (adjusted *p*-value 3.67e-19), and Reference proteome (adjusted *p*-value 1.05e-31) peptide results, while the assignment of statistically significant differences between annotated and novel peptide group PEP scores varied between the six frame database (adjusted *p*-value 1.89e-06), and *GeneMarkS* database (adjusted *p*-value 8.75e-01), although the values were much higher than the annotated-reverse comparisons. Further, the comparison between novel and reverse peptide group PEP scores indicated a significant difference for both the six frame database (adjusted *p*-value 9.84e-13) and *GeneMarkS* database (adjusted *p*-value 5.50e-17) comparisons—with much lower adjusted *p*-values than those of the annotated-novel group comparisons of both databases. PEP score analysis was also performed on the combined PSMs for each database obtained from the msms.txt file, supporting the below analysis (see Supplementary Table 9, Supplementary Figure 2, and Supplementary Data Sheet 6.

### Reference proteome database

**Figure 8:**
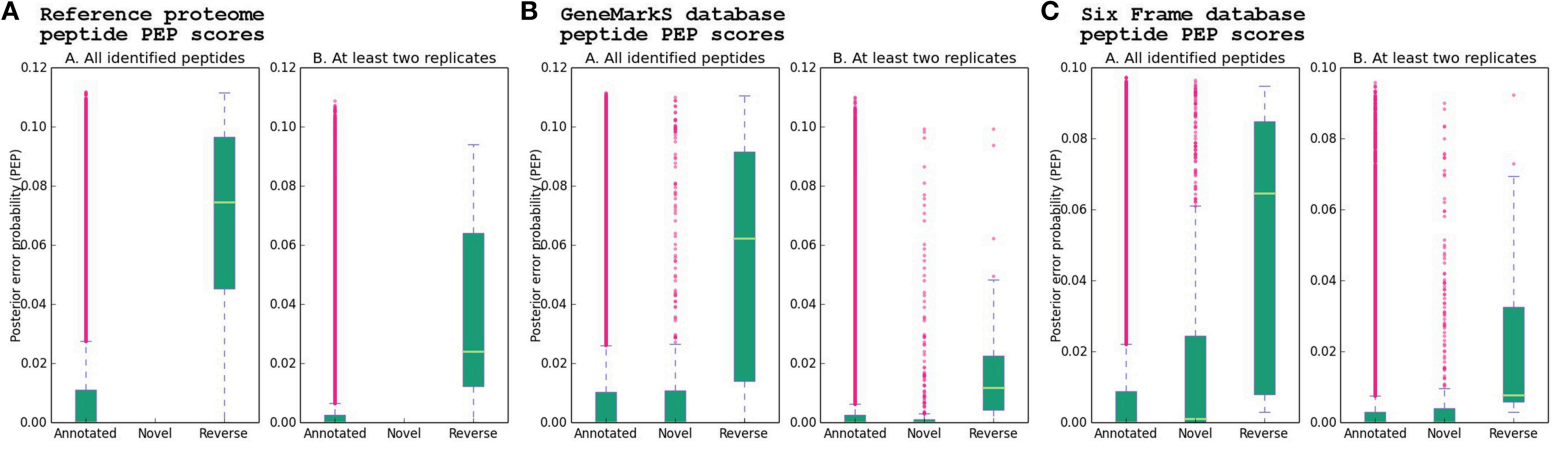
**Peptide PEP score distribution**. The posterior error probability (PEP) score distribution of novel, annotated and reverse hit peptide identifications obtained using three different database searches are shown, using the PEP score of the best PSM obtained for every peptide, from the *MaxQuant* peptides.txt output file. **(A)** Reference proteome, **(B)** *GeneMarkS* database, **(C)** Six frame database. The whiskers represent 1.5 times the interquartile range (IQR) below and above the first and third quartile, respectively. Differences in the means of the groups from each database search were evaluated using the non-parametric Kruskal–Wallis test, which indicated significant differences between the means of novel and reverse peptide PEP scores in the *GeneMarkS* (adjusted *p*-value 5.50e-17) and six frame database (adjusted *p*-value 9.84e-13). The *p*-values assigned to the comparisons between annotated and novel groups for the *GeneMarkS* (adjusted *p*-value 8.75e-01) and six frame (adjusted *p*-value 1.89e-06) databases were much larger than the *p*-values assigned to the comparisons between novel and reverse groups. Significant differences were found in the annotated-reverse comparisons for all three databases—*GeneMarkS* (adjusted *p*-value 6.68e-21), six frame database (adjusted *p*-value 3.67e-19) and Reference proteome (adjusted *p*-value1.050e-31). The effect of excluding peptides seen only in a single replicate on the PEP score distributions is also apparent. The adjusted *p*-value assigned to the comparison between all six frame novel and annotated peptides (adjusted *p*-value 1.89e-06) was much smaller than the adjusted *p*-value assigned to the same comparison after excluding peptides seen only in a single replicate (adjusted *p*-value 1.77e-02). In contrast, the comparison between six frame novel and reverse peptides seen in at least two replicates was assigned an adjusted *p*-value of 2.87e-06, which is closer to the *p*-value assigned to the comparison between annotated and reverse peptides seen in at least two replicates (adjusted *p*-value 6.09e-08; see Supplementary Table 10 and Supplementary Data Sheet 5 for the results of Kruskal–Wallis tests and *post-hoc* pairwise comparisons).

Using the Reference proteome database, 27,720 annotated peptides were identified, with a median PEP score of 1.32E-04. Further, 72 reverse hit peptides were reported by *MaxQuant*, with a median PEP score of 7.46E-02. Kruskal–Wallis and Dunn's test *post-hoc* analysis showed a statistically significant difference between the two groups (adjusted *p*-value 1.05e-31 before excluding peptides seen in only one replicate). After selecting peptides identified in at least two replicates, 2788 database sequences were identified with at least one unique peptide. For the group of Reference proteins with TSS validation and single genomic coordinates, a start site distribution of ATG 353 (64.53%), TTG 4 (0.73%), and GTG 190 (34.73%) was determined (see Figures 2, 8A, Table 1, and Supplementary Table 10).

**Table 1:** **Database identifications**.

	**Reference proteome**	**Six frame database**	***GeneMarkS* database**	**Combined**
Reference protein ORF identifications	2788	2753	2773	2810
Reference TSS validations	554	282	439	558
All upstream gene model modifications	0	70	42	81
Upstream TSS identifications	0	29	28	39
Downstream TSS identifications	0	0	24	24
Novel ORF identifications	0	63	42	63

*A table comparing Reference protein ORF identifications, TSS validations, upstream gene model modifications, upstream TSS identifications, downstream TSS identifications and novel ORF identifications using three different databases*.

### *GenemarkS* database

The *GeneMarkS* database allowed for the identification of 27,168 annotated and 407 novel peptides, with a median peptide PEP score of 1.16E-04 and 2.22E-04 respectively, and 59 reverse hit peptides with a median peptide PEP score of 6.24E-02. The PEP score distribution of annotated and novel peptides were not significantly different using Kruskal–Wallis and *post-hoc* tests (adjusted *p*-value 8.75e-01), and from the boxplot visualizations appear similar to the PEP distribution of annotated peptides obtained from the Reference proteome database. A significant difference was found between annotated and reverse peptide PEP score distributions (adjusted *p*-value 6.68e-21). A comparison between novel and reverse peptide PEP score distributions revealed a significant difference (adjusted *p*-value 5.50e-17), and the number of reverse hit peptides identified was markedly lower than the number of novel peptides identified. Thus, it is very likely that most novel identifications are true positive identifications (see Figure 8B). After excluding peptides only identified in a single sample, 2815 *GeneMarkS* sequences were identified with at least one unique peptide. Of these, 42 were not annotated to a Reference protein. Further, in the group of 558 Reference protein TSS validations, 442 sequences had a corresponding entry in the *GeneMarkS* database with the correct TSS prediction (of which 439 were identified)—while only one did not have a corresponding entry, and 115 had a corresponding entry with an alternative TSS assignment. Thus, in the group of TSS validations with a corresponding *GeneMarkS* entry, *GeneMarkS* had correctly predicted the TSS in 79.35% cases. Further in the set of all identified genomic ORFs annotated to a Reference protein (2810), GeneMarkS had predicted the ORF as protein-coding in 99.68% of cases. Of the 81 upstream gene model modifications, 80 had a corresponding *GeneMarkS* sequence identified, of which 42 of the modifications were supported by upstream peptide identifications from the *GeneMarkS* database (with 28 TSS identifications). All of the 24 downstream gene model modifications were identified with TSS evidence from the *GeneMarkS* database, with a mean N-terminal shortening of ~10 amino acids in this group (see Figure 2, Table 1, and Supplementary Table 10).

#### Six frame database

The translated six frame database allowed for the identification of 25,427 annotated and 553 novel peptides, with a median peptide PEP score of 1.07E-04 and 1.04E-03 respectively, and 48 reverse hit peptides with a median PEP score of 6.47E-02. Using Kruskal–Wallis and Dunn's test *post-hoc* analysis, a significant difference between the group PEP scores of annotated and reverse (adjusted *p*-value 3.67e-19), annotated and novel (adjusted *p*-value 1.89e-06), and novel and reverse (adjusted *p*-value 9.84e-13) peptide identifications was found. After selecting peptides seen in at least two replicates, a much larger although still statistically significant adjusted *p*-value of 1.77e-02 for the comparison between novel and annotated groups was obtained—possibly indicating a relatively higher proportion of false positive identifications in the set of novel peptide identifications than in the annotated set. *Post-hoc* comparison between novel and reverse peptide PEP score distributions revealed adjusted *p*-values of 9.84e-13 and 2.87e-06 before and after excluding peptides seen in only one replicate respectively—much lower than the *p*-values obtained from the comparison between novel and annotated peptide groups—indicating a much greater difference between the distribution of novel and reverse peptide PEP scores than between novel and annotated peptides, see Figure 8C. After selecting peptides seen in at least two replicates, 2825 ORFs were identified using the six frame database. Seventy-two ORFs identified using the six frame database mapped to non-Reference proteome ORFs. Of the 558 Reference protein sequences with TSS validations, a corresponding six frame sequence was identified in 544 cases. In this group, 299—or 54.96%—of the six frame sequences (translated from the most upstream start codon in the ORF) correctly assigned the TSS. On average, the six frame database overestimated the correct TSS by ~32 amino acids. Further, in the group of 81 upstream gene model modifications, 80 cases had a corresponding six frame sequence identified. In this group, 87.5% of the upstream gene model modifications were supported by the identification of upstream peptide evidence using the six frame database. Of the 38 sequences with identified TSS sites in this group, 29 were correctly assigned in the six frame database (see Figure 2, Table 1, and Supplementary Table 10).

## Discussion

Validation of genomic information by proteomic data has gained momentum over the past few years. The improvement in genome annotation can give rise to a better understanding of the biology of pathogenic organisms and ultimately, new strategies for disease prevention and treatment. Mass-spectrometry data has proven invaluable in terms of providing evidence of the translation of genes expressed under differing conditions.

By mapping identified proteins from the different databases to their corresponding ORFs in the genome, 2887 ORFs were identified at the peptide level. We showed that the identified start codon distribution of all identified ORFs with TSS identifications corresponded to previously reported findings in Mycobacteria, with a relatively higher percentage of GTG, although a relatively lower proportion of TTG start codons was identified. The identified penultimate amino acids of N-Met cleaved peptides corresponded to previously published findings, although the preponderance of *Thr* was notable.

We identified 2810 Reference proteins at the peptide level, and using N-terminal peptide evidence we validated the TSS for 558 of these. Further, 81 gene model modifications were indicated by the identification of peptides mapping upstream, and 24 by N-terminal peptides mapping downstream, of the annotated TSS of a Reference protein.

We provide experimental evidence for 63 novel ORFs not previously verified at the protein level. A total of 44 ORFs were identified with two or more peptides—of which 30 aligned to predicted or inferred non-Reference proteome sequences of *M. smegmatis* mc^2^155. A single ORF was identified with two peptides but did not yield any protein BLAST alignments. Further, 19 novel ORFs were identified with a single peptide but were supported by either protein BLAST alignment or a previously identified transcript overlapping the ORF on the genome on the same strand, and are presented as lower-ranking evidence for annotation. We also identified six previously reported interrupted coding sequences caused by sequencing errors, with peptides identified on either side of a frameshift position—four of which do not appear to have been previously identified at the peptide level. Further, we identified evidence for four novel ICDSs, three of which were supported by alignment to orthologous sequence evidence from a newly sequenced strain of *M. smegmatis*, emphasizing the importance of continuous review of genome annotation as new information becomes available.

In this study, we analyzed LC/MS/MS data with three different sequence databases to improve the genome annotation of *M. smegmatis* mc^2^155; two genomic—a six frame translation and a *GeneMarkS* gene prediction database—and the UniProt Reference proteome^9^. In both genomic databases, the PEP score distribution of novel peptides was much closer to that of annotated peptides than reverse sequence hits, and almost identical in the *GeneMarkS* database—indicating that most novel peptide identifications are likely to be true positive identifications. A higher number of total peptide identifications was attained using *GeneMarkS* than the six frame database—closely approaching the number attained using the Reference proteome. We report a high percentage of accurate TSS predictions using *GeneMarkS*—79.35% in the group of Reference protein TSS validations where a corresponding *GeneMarkS* entry was predicted. This indicates that proteogenomic database generation with targeted ORF and TSS predictions using *de novo* gene prediction tools such as *GeneMarkS* can fruitfully decrease the database search space, increasing the sensitivity of peptide identifications. However, database compaction is a compromise between reducing spurious possibilities, and minimizing the exclusion of non-spurious entries from the search space. ORF and TSS assignments will depend on the particular gene prediction tool used, and will not be detected if incorrectly assigned at the database generation phase. By including a six frame translation database in the pipeline, ORFs missed by the gene prediction algorithm, as well as upstream peptide evidence and TSS site identifications not included in the gene prediction database, may be identified.

Considering the PEP score distributions of novel and annotated peptides, and the much smaller number of reverse sequence hits than novel peptide identifications, it is evident that the proteome of *M. smegmatis* mc^2^155 is not yet fully characterized and more work needs to be done to identify novel protein coding genes before a complete genome annotation status is attained. We hope that the evidence we present here will lead to the addition of new sequences to the Reference proteome of this strain, supporting the downstream functional characterization of these proteins, thus leading to a better understanding of the biology of an important model organism of the infectious *M. tuberculosis*. The gene model modifications we present for many Reference proteome sequences—many of which have not yet been identified at the protein level—facilitates improved functional and structural characterization of these proteins, allowing for accurate conclusions to be drawn by downstream comparative proteomics analyses.

## Author contributions

MP, MSc student, writing, Bioinformatics component; KN, writing, Proteomics component; JA, technical support on Bioinformatics component; AN, technical support on Proteomics component; SG, technical support on Proteomics component; NS, writing, technical support on Proteomics component; JB, corresponding author, technical supervision, and assistance, Proteomics component; NM, corresponding author, technical supervision and assistance, proof reading, Bioinformatics component.

### Conflict of interest statement

The authors declare that the research was conducted in the absence of any commercial or financial relationships that could be construed as a potential conflict of interest.

## Footnotes

^1^http://tuberculist.epfl.ch/

^2^*GeneMark*^TM^—*Free Gene Prediction Software.”* http://exon.gatech.edu/GeneMark/(Accessed September 21, 2015).

^3^*UniProt*. http://www.uniprot.org/ (Accessed September 21, 2015).

^4^http://www.ebi.ac.uk/pride/archive/

^5^*Biopython-Biopython*. Available online at: http://biopython.org/wiki/Main_Page (Accessed September 21, 2015).

^6^ftp://ftp.ensemblgenomes.org/pub/bacteria/current/fasta/bacteria_7_collection/mycobacterium_smegmatis_str_mc2_155/cdna/

^7^http://bacteria.ensembl.org/Mycobacterium_smegmatis_str_mc2_155/Info/Index

^8^*Retrieve/ID Mapping (UniProt)*. http://www.uniprot.org/uploadlists/

^9^*Mycobacterium smegmatis Reference Proteome*. *UniProt Proteomes*. http://www.uniprot.org/proteomes/UP000000757 (Accessed August 9, 2015).

## Supplementary material

Supplementary Figure 1 **Novel ORF ICDSs**. Shows peptide evidence for six previously identified ICDSs—of which only two have previously been reported at the protein level—and four novel ICDS sequences. **(A)** DQ866867: A predicted protein spanning a sequencing error reported by Deshayes et al. (2007), showing peptides on either side of a frame shift position. **(B)** DQ866856: A protein spanning a sequencing error reported by Deshayes et al. (2007), which they also identified at the peptide level, showing peptides on either side of the frameshift position. **(C)** DQ866859: Peptide evidence for a predicted protein spanning a sequencing error reported by Deshayes et al. (2007). **(D)** DQ866863: Peptide evidence for a predicted protein spanning a sequencing error reported by Deshayes et al. (2007). **(E)** DQ866858: Peptide evidence for a predicted protein spanning a sequencing error reported by Deshayes et al. (2007), showing an upstream peptide identified in an overlapping ORF (highlighted in red). **(F)** Novel ICDS: An upstream ORF first predicted by Deshayes et al. in 2007 (I7FS93_MYCS2), with downstream peptide evidence in an overlapping novel ORF (three peptides). Both ORFs align to A0A0K0X632_9MYCO (peptidase M75 of *M. goodii*), with very low *E*-values (0.0 and 5.03e-09, respectively). The downstream peptides are highlighted in red. **(G)** DQ866873: an ICDS spanning a sequencing error, and also detected at the protein level, reported by Deshayes et al. (2007). Five upstream peptides in an overlapping novel ORF are highlighted in red. **(H)** Novel ICDS: Two adjacent ORFs in alternate reading frames, both aligned to the protein L8F5X0_MYCSM—predicted by Gray et al. (2013) from the genome of *M. smegmatis* MKD8. **(I)** Novel ICDS: Two adjacent ORFs in alternate reading frames, both aligned to the protein L8FGK0_MYCSM—predicted by Gray et al. (2013) from the genome of *M. smegmatis* MKD8. **(J)** Novel ICDS: Two adjacent ORFs in alternate reading frames, both aligned to the protein L8F9F0_MYCSM—predicted by Gray et al. (2013) from the genome of *M. smegmatis* MKD8. A single upstream peptide in an alternate frame is highlighted in red.

Supplementary Figure 2 **PSM PEP score distribution**. Shows the PEP score distributions of all annotated, novel and reverse sequence PSMs from the different databases. The whiskers represent 1.5 times the interquartile range (IQR) below and above the first and third quartile, respectively. In the *GeneMarkS* and six frame database groups the PEP score distribution of novel PSMs is noticeably closer to the group of annotated PSMs than reverse hits, indicating the high confidence of novel PSM identifications. PEP score distributions are further improved by excluding the PSMs of peptides that were only identified in a single replicate. Using the Kruskal–Wallis test followed by *post-hoc* analysis, significant differences were found for the comparisons between all novel and reverse PSM PEP scores for both the *GeneMarkS* (adjusted *p*-value 2.20e-42) and six frame databases (adjusted *p*-value 8.01e-36), respectively. Similarly, significant differences were found between all annotated and all reverse group PSM PEP scores for the Reference proteome (adjusted *p*-value 1.71e-70), *GeneMarkS* (adjusted *p*-value 2.00e-48) and six frame database (adjusted *p*-value 1.83e-44). The *p*-values assigned to the comparisons between all annotated and all novel PSM PEP scores were much higher for both the *GeneMarkS* (adjusted *p*-value 4.26e-02) and six frame database (adjusted *p*-value 6.83e-09; see Supplementary Data Sheet 6) for the results of Kruskal–Wallis tests and *post-hoc* pairwise comparisons.

Supplementary Table 1 **Combined ORF identifications**.

Supplementary Table 2 **Multiple initiation**.

Supplementary Table 3 **Validated TSSs**.

Supplementary Table 4 **Frameshift validation**.

Supplementary Table 5 **Upstream start annotations**.

Supplementary Table 6 **Downstream start annotations**.

Supplementary Table 7 **Novel ORF annotations**.

Supplementary Table 8 **Novel ORF ICDSs**.

Supplementary Table 9 **PSM PEP scores**.

Supplementary Table 10 **Peptide PEP scores**.

Data Sheet 1 **GeneMarkS sequence database (GFF format)**.

Data Sheet 2 **Six frame database peptide identifications (GFF format)**.

Data Sheet 3 **GeneMarkS database peptide identifications (GFF format)**.

Data Sheet 4 **Reference proteome peptide identifications (GFF format)**.

Data Sheet 5 **Analysis of best peptide PSM PEP scores**.

Data Sheet 6 **Analysis of all peptide PSM PEP scores**.
