# Supplementary figures and images for: Proteogenomic Analysis of Mycobacterium smegmatis Using High Resolution Mass Spectrometry
Source: Front Microbiol. 2016 Apr 5;7:427. doi: 10.3389/fmicb.2016.00427 (PMC4821088; doi:10.3389/fmicb.2016.00427)

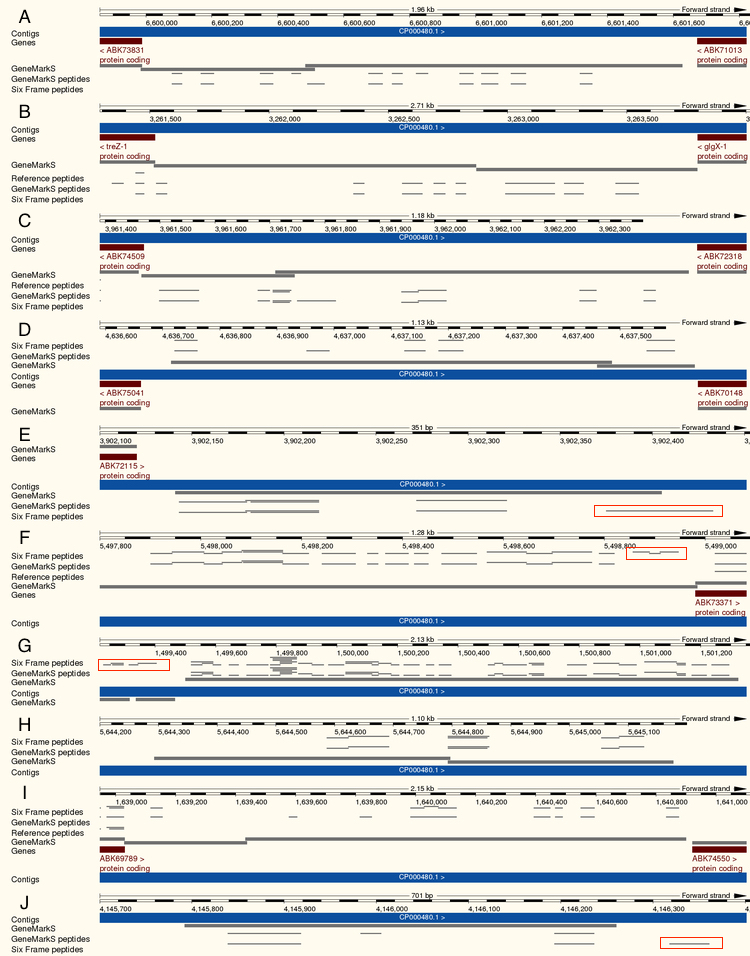

Supplement: Supplementary Figure 1 — Novel ORF ICDSs. Shows peptide evidence for six previously identified ICDSs—of which only two have previously been reported at the protein level—and four novel ICDS sequences. (A) DQ866867: A predicted protein spanning a sequencing error reported by Deshayes et al. (2007), showing peptides on either side of a frame shift position. (B) DQ866856: A protein spanning a sequencing error reported by Deshayes et al. (2007), which they also identified at the peptide level, showing peptides on either side of the frameshift position. (C) DQ866859: Peptide evidence for a predicted protein spanning a sequencing error reported by Deshayes et al. (2007). (D) DQ866863: Peptide evidence for a predicted protein spanning a sequencing error reported by Deshayes et al. (2007). (E) DQ866858: Peptide evidence for a predicted protein spanning a sequencing error reported by Deshayes et al. (2007), showing an upstream peptide identified in an overlapping ORF (highlighted in red). (F) Novel ICDS: An upstream ORF first predicted by Deshayes et al. in 2007 (I7FS93_MYCS2), with downstream peptide evidence in an overlapping novel ORF (three peptides). Both ORFs align to A0A0K0X632_9MYCO (peptidase M75 of M. goodii), with very low E-values (0.0 and 5.03e-09, respectively). The downstream peptides are highlighted in red. (G) DQ866873: an ICDS spanning a sequencing error, and also detected at the protein level, reported by Deshayes et al. (2007). Five upstream peptides in an overlapping novel ORF are highlighted in red. (H) Novel ICDS: Two adjacent ORFs in alternate reading frames, both aligned to the protein L8F5X0_MYCSM—predicted by Gray et al. (2013) from the genome of M. smegmatis MKD8. (I) Novel ICDS: Two adjacent ORFs in alternate reading frames, both aligned to the protein L8FGK0_MYCSM—predicted by Gray et al. (2013) from the genome of M. smegmatis MKD8. (J) Novel ICDS: Two adjacent ORFs in alternate reading frames, both aligned to the protein L8F9F0_MYCSM—predicted by Gray et al. (2013) fro [file Image1.JPEG]

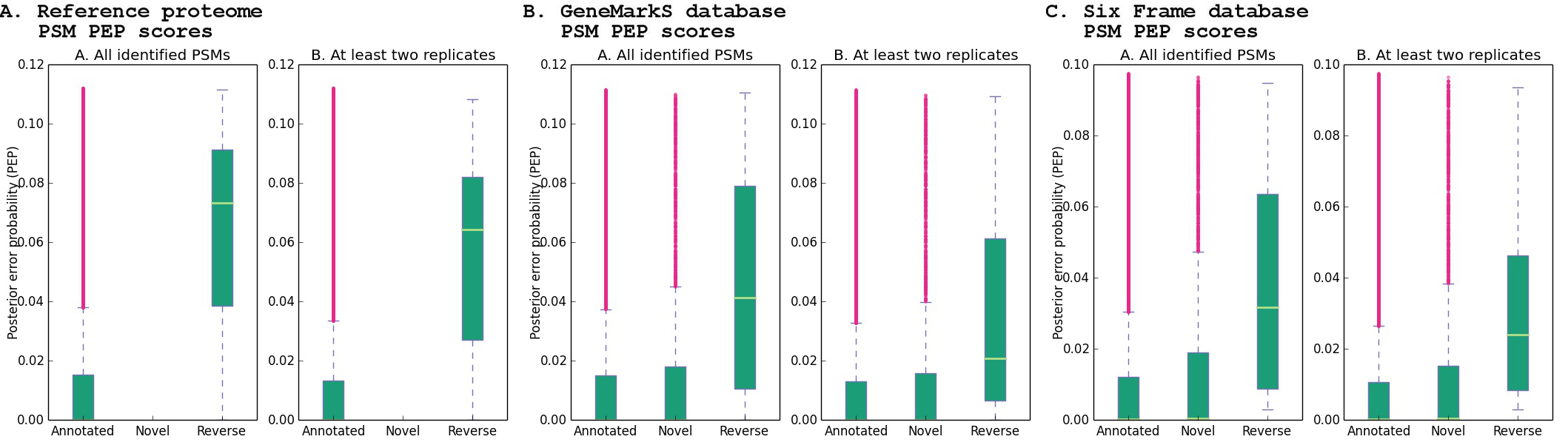

Supplement: Supplementary Figure 2 — PSM PEP score distribution. Shows the PEP score distributions of all annotated, novel and reverse sequence PSMs from the different databases. The whiskers represent 1.5 times the interquartile range (IQR) below and above the first and third quartile, respectively. In the GeneMarkS and six frame database groups the PEP score distribution of novel PSMs is noticeably closer to the group of annotated PSMs than reverse hits, indicating the high confidence of novel PSM identifications. PEP score distributions are further improved by excluding the PSMs of peptides that were only identified in a single replicate. Using the Kruskal–Wallis test followed by post-hoc analysis, significant differences were found for the comparisons between all novel and reverse PSM PEP scores for both the GeneMarkS (adjusted p-value 2.20e-42) and six frame databases (adjusted p-value 8.01e-36), respectively. Similarly, significant differences were found between all annotated and all reverse group PSM PEP scores for the Reference proteome (adjusted p-value 1.71e-70), GeneMarkS (adjusted p-value 2.00e-48) and six frame database (adjusted p-value 1.83e-44). The p-values assigned to the comparisons between all annotated and all novel PSM PEP scores were much higher for both the GeneMarkS (adjusted p-value 4.26e-02) and six frame database (adjusted p-value 6.83e-09; see Supplementary Data Sheet 6) for the results of Kruskal–Wallis tests and post-hoc pairwise comparisons. [file Image2.JPEG]

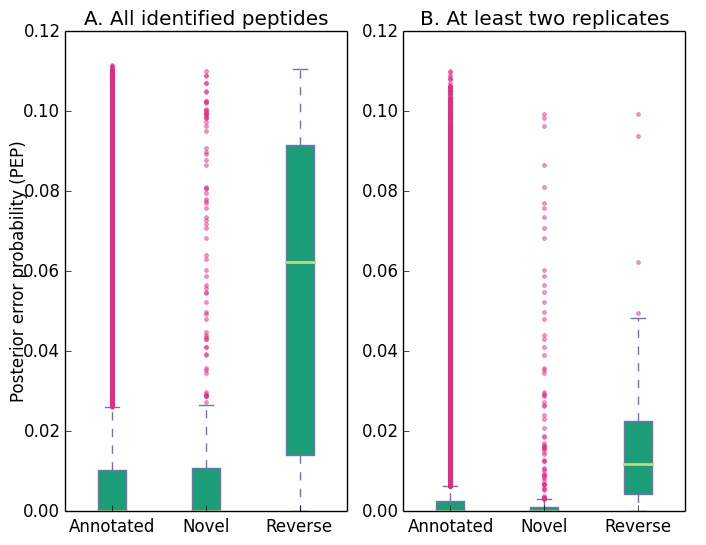

Supplement: Data Sheet 5 — Analysis of best peptide PSM PEP scores. [file DataSheet5.ZIP › Supplementary Data Sheet 5_ Analysis of best peptide PSM PEP scores/PEP_boxplots/GeneMarkS database PEP.jpg]

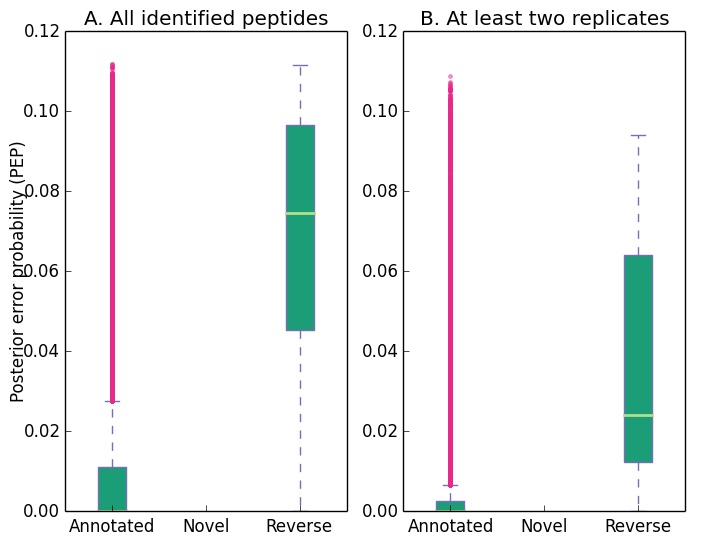

Supplement: Data Sheet 5 — Analysis of best peptide PSM PEP scores. [file DataSheet5.ZIP › Supplementary Data Sheet 5_ Analysis of best peptide PSM PEP scores/PEP_boxplots/Reference proteome PEP.jpg]

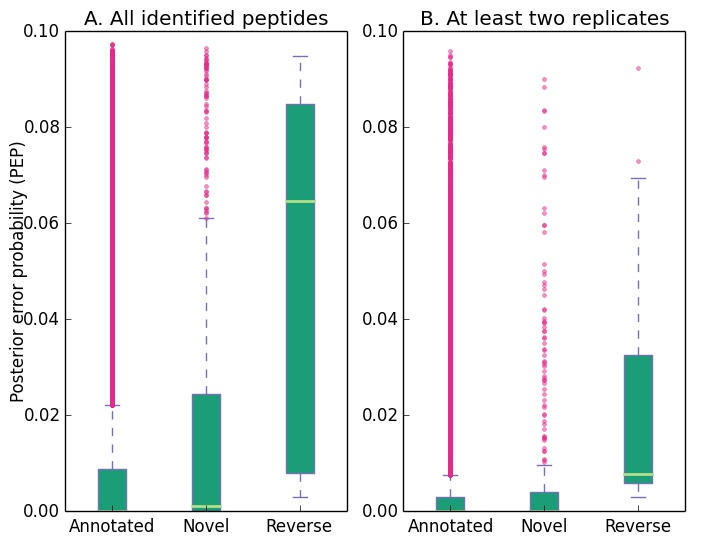

Supplement: Data Sheet 5 — Analysis of best peptide PSM PEP scores. [file DataSheet5.ZIP › Supplementary Data Sheet 5_ Analysis of best peptide PSM PEP scores/PEP_boxplots/Six Frame database PEP.jpg]

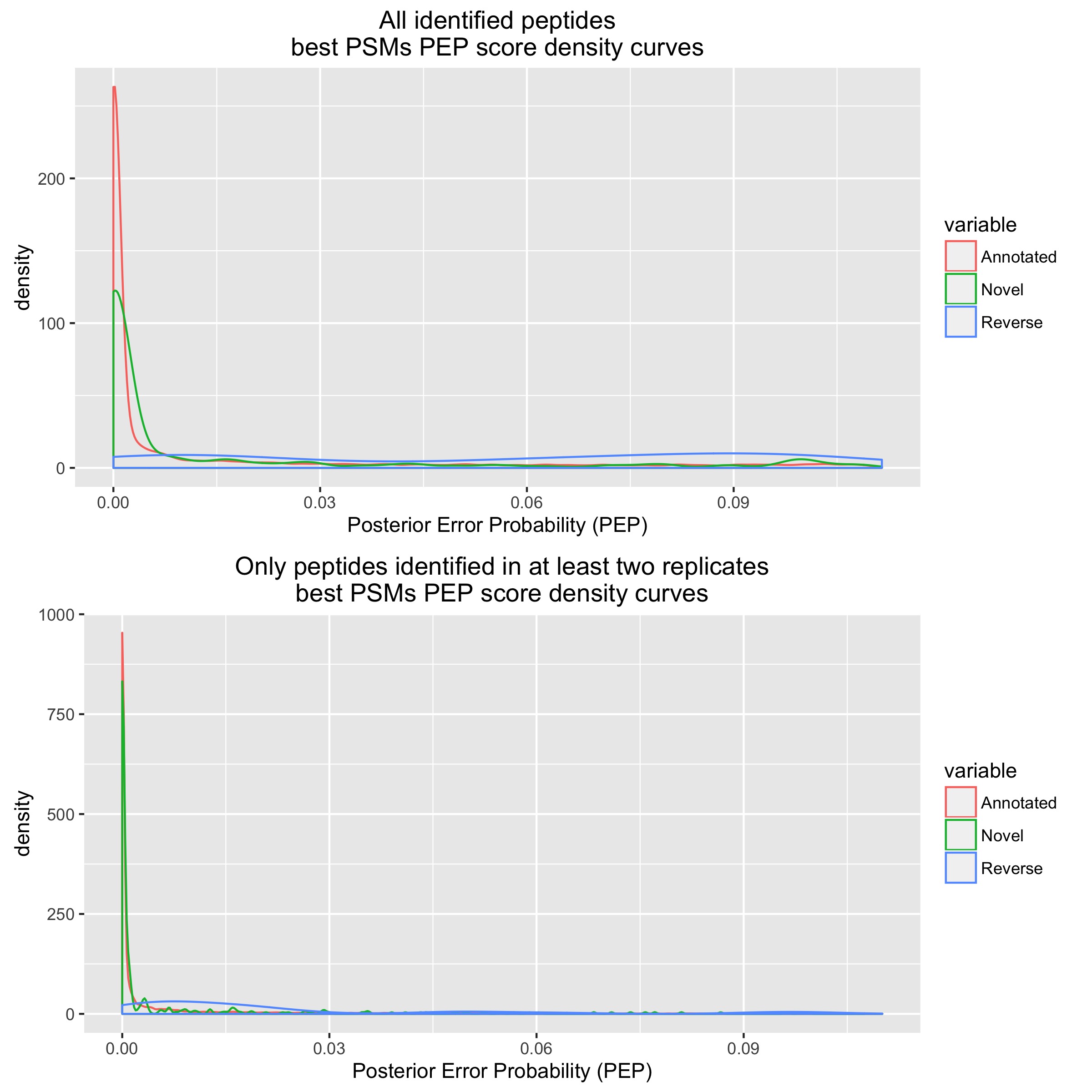

Supplement: Data Sheet 5 — Analysis of best peptide PSM PEP scores. [file DataSheet5.ZIP › Supplementary Data Sheet 5_ Analysis of best peptide PSM PEP scores/PEP_density/GeneMarkS_database_MSMS_PEP_score_density.jpeg]

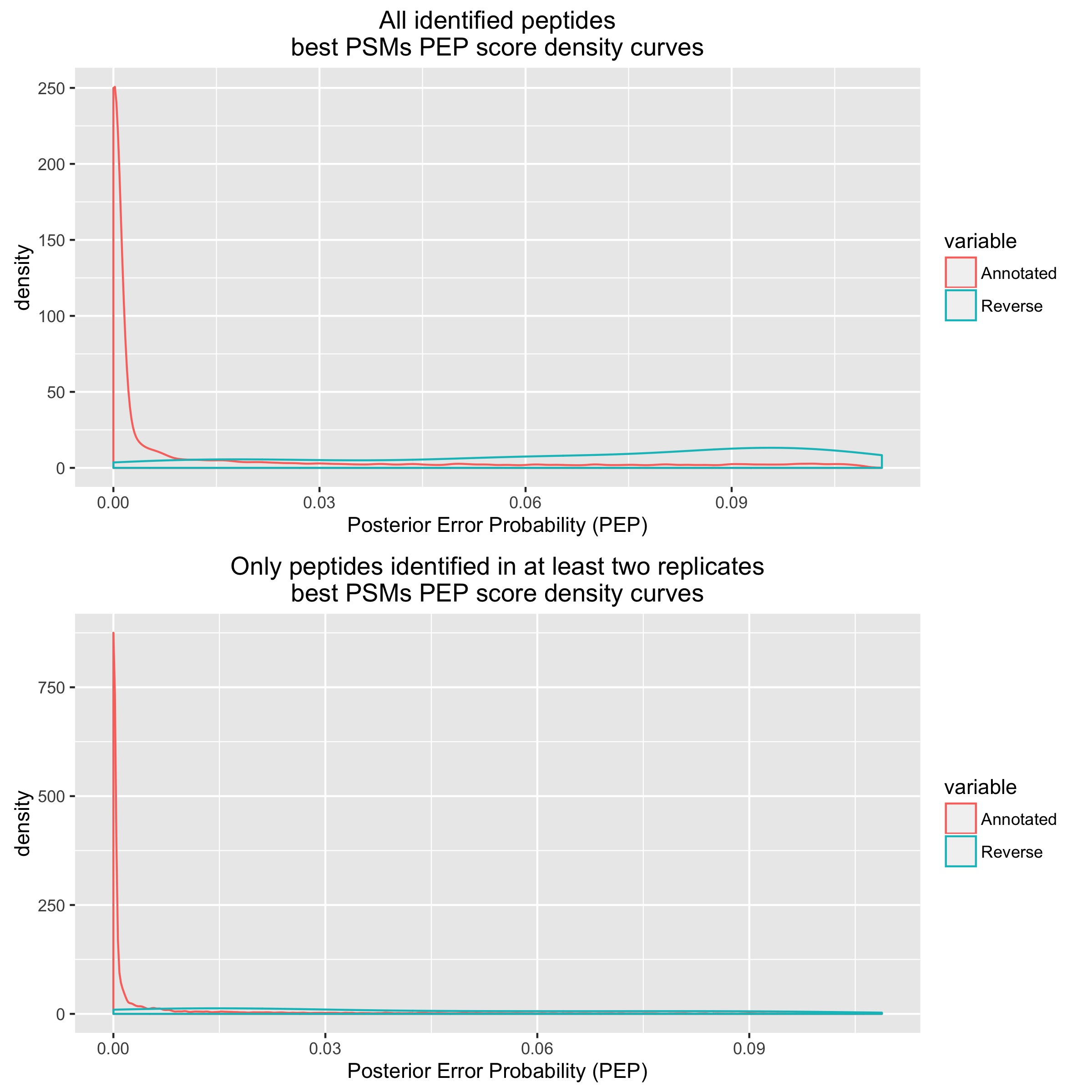

Supplement: Data Sheet 5 — Analysis of best peptide PSM PEP scores. [file DataSheet5.ZIP › Supplementary Data Sheet 5_ Analysis of best peptide PSM PEP scores/PEP_density/Reference_proteome_MSMS_PEP_score_density.jpeg]

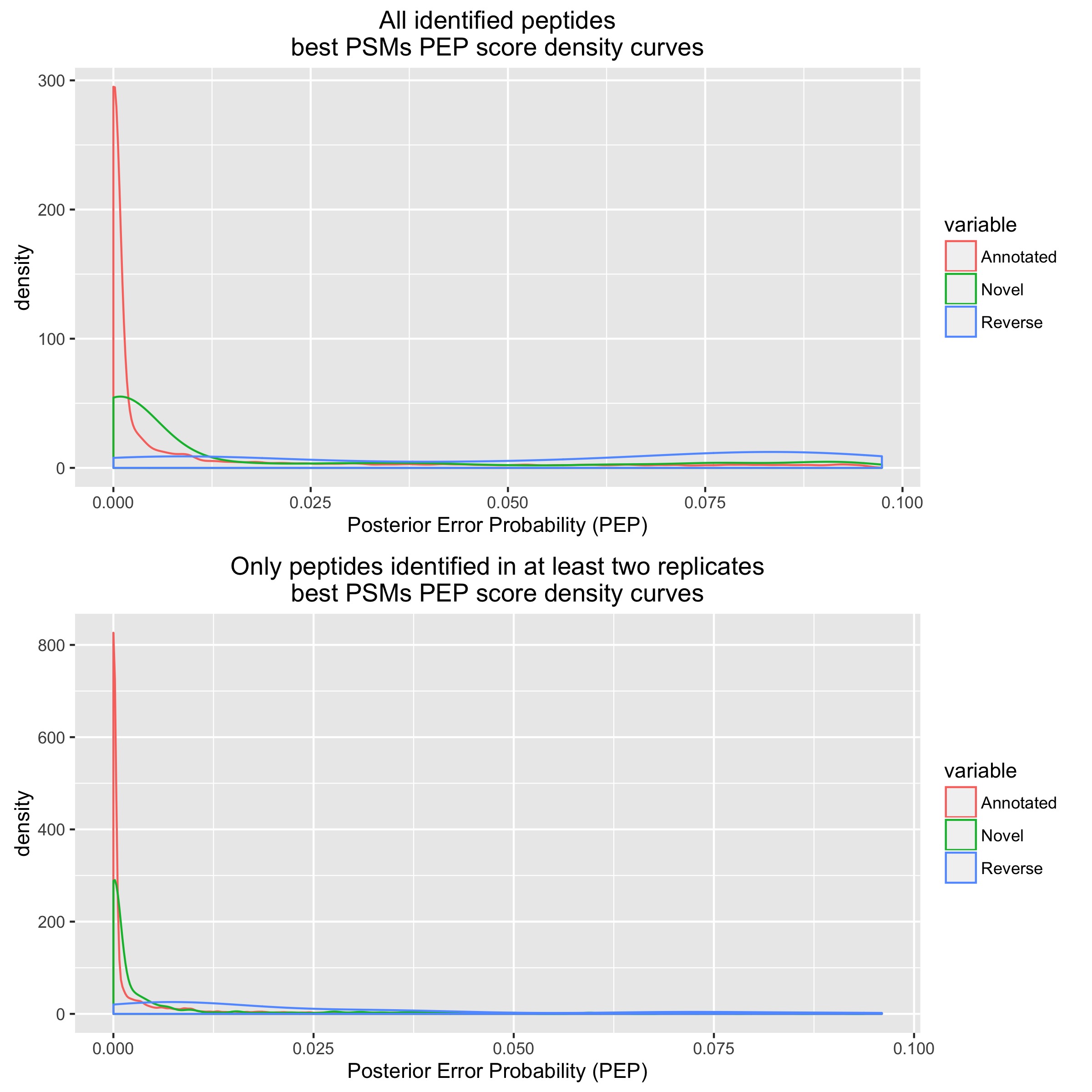

Supplement: Data Sheet 5 — Analysis of best peptide PSM PEP scores. [file DataSheet5.ZIP › Supplementary Data Sheet 5_ Analysis of best peptide PSM PEP scores/PEP_density/Six_Frame_database_MSMS_PEP_score_density.jpeg]

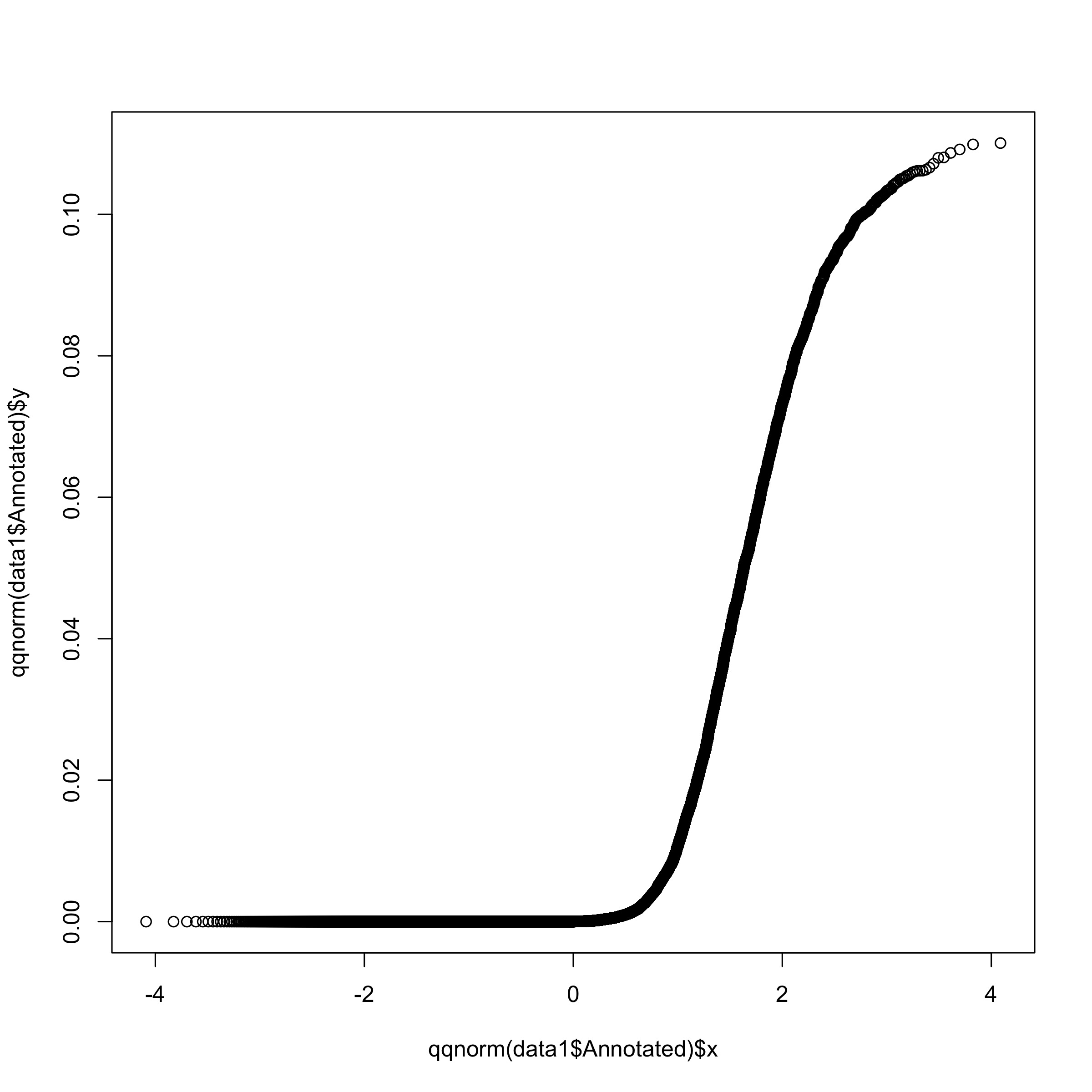

Supplement: Data Sheet 5 — Analysis of best peptide PSM PEP scores. [file DataSheet5.ZIP › Supplementary Data Sheet 5_ Analysis of best peptide PSM PEP scores/PEP_qqnorm/GeneMarkS_database/Annoted_PEP_2_reps.jpeg]

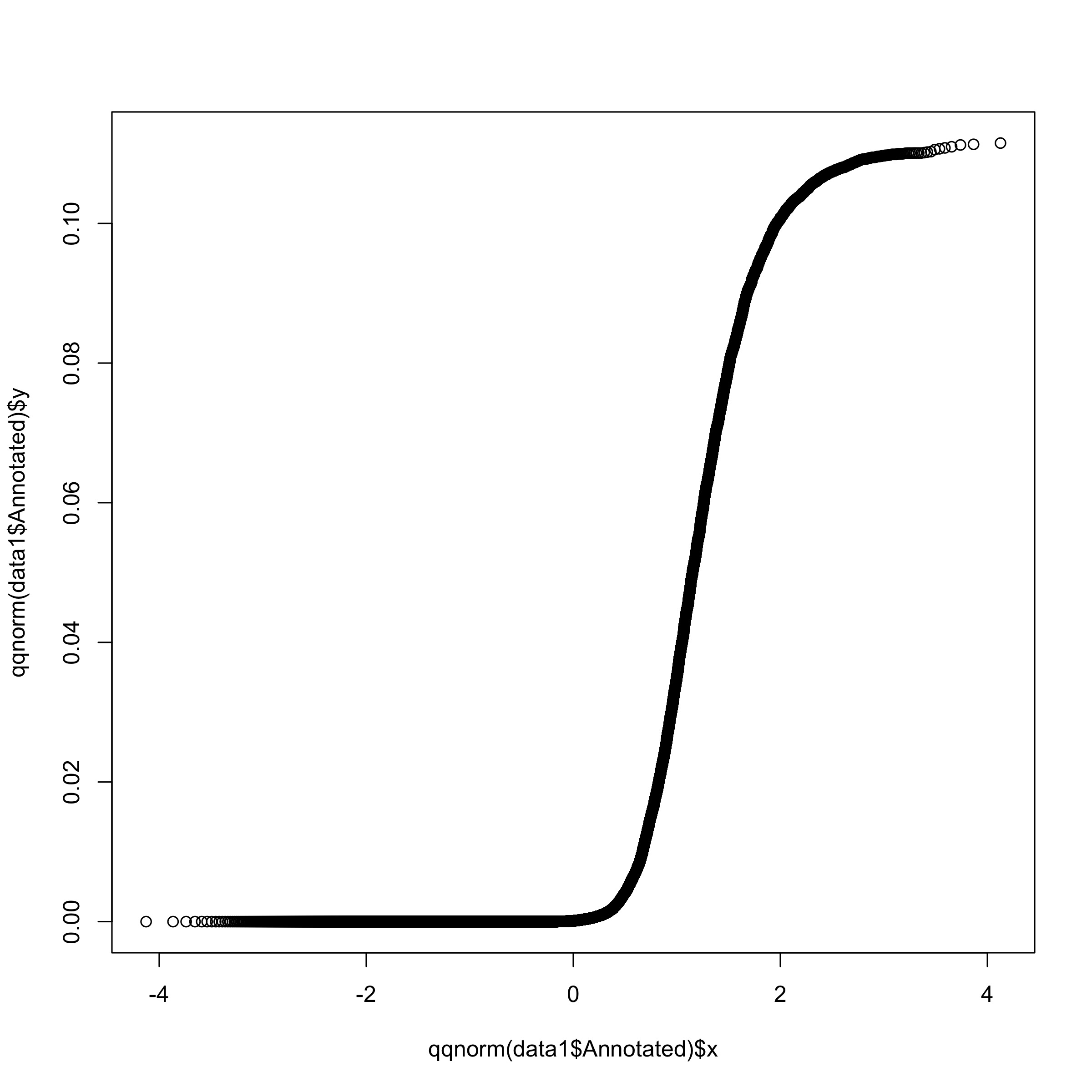

Supplement: Data Sheet 5 — Analysis of best peptide PSM PEP scores. [file DataSheet5.ZIP › Supplementary Data Sheet 5_ Analysis of best peptide PSM PEP scores/PEP_qqnorm/GeneMarkS_database/Annoted_PEP_all.jpeg]

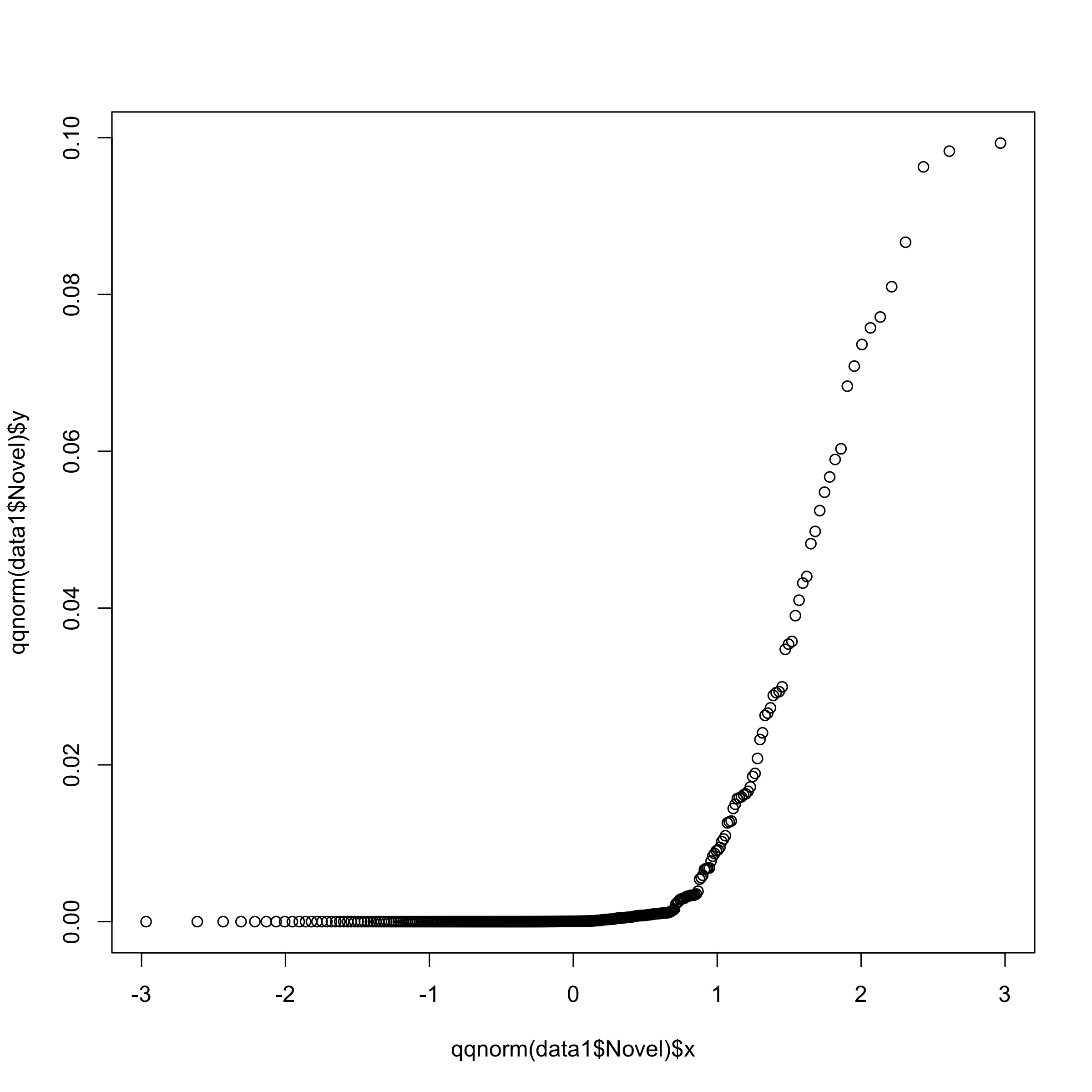

Supplement: Data Sheet 5 — Analysis of best peptide PSM PEP scores. [file DataSheet5.ZIP › Supplementary Data Sheet 5_ Analysis of best peptide PSM PEP scores/PEP_qqnorm/GeneMarkS_database/Novel_PEP_2_reps.jpeg]

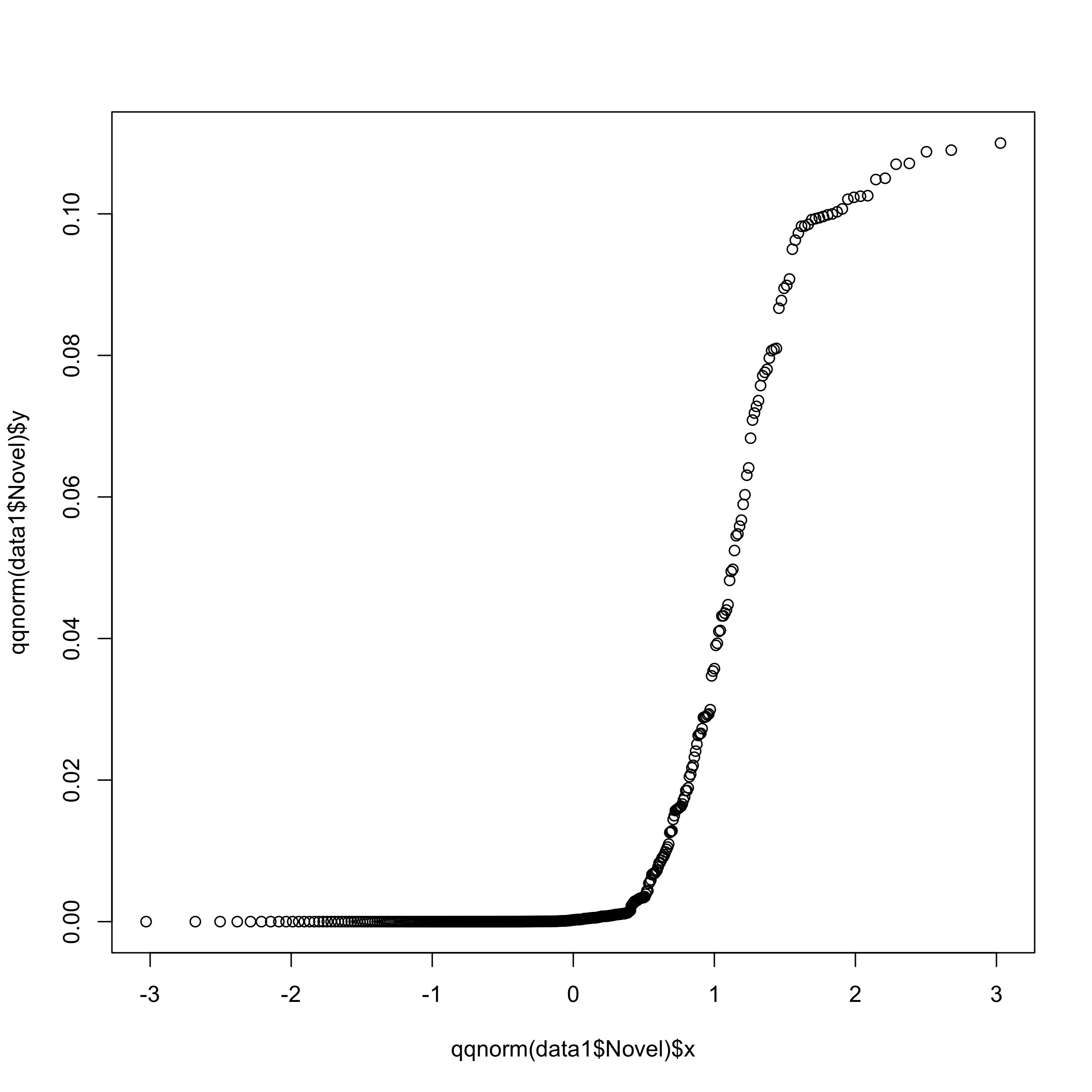

Supplement: Data Sheet 5 — Analysis of best peptide PSM PEP scores. [file DataSheet5.ZIP › Supplementary Data Sheet 5_ Analysis of best peptide PSM PEP scores/PEP_qqnorm/GeneMarkS_database/Novel_PEP_all.jpeg]

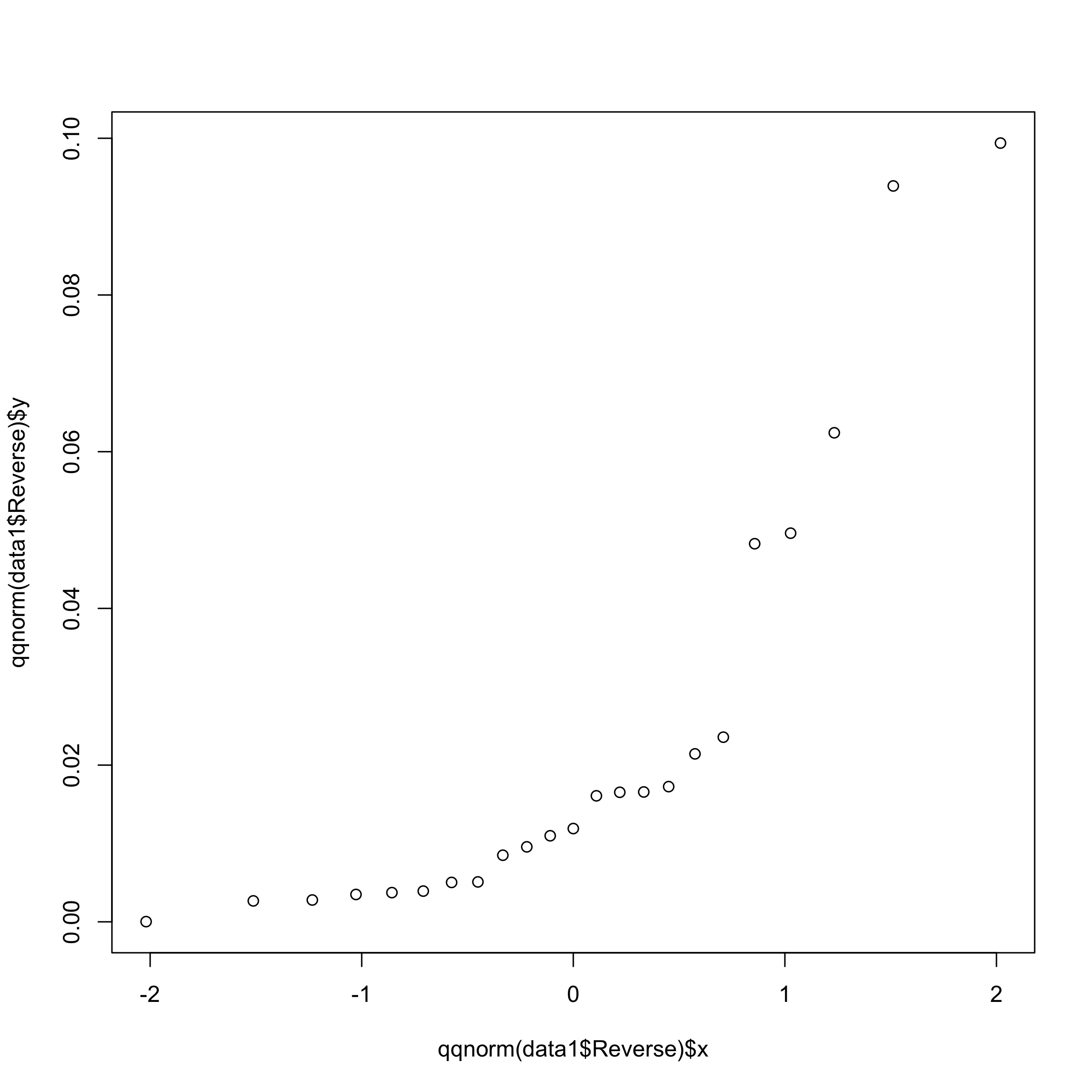

Supplement: Data Sheet 5 — Analysis of best peptide PSM PEP scores. [file DataSheet5.ZIP › Supplementary Data Sheet 5_ Analysis of best peptide PSM PEP scores/PEP_qqnorm/GeneMarkS_database/Reverse_PEP_2_reps.jpeg]

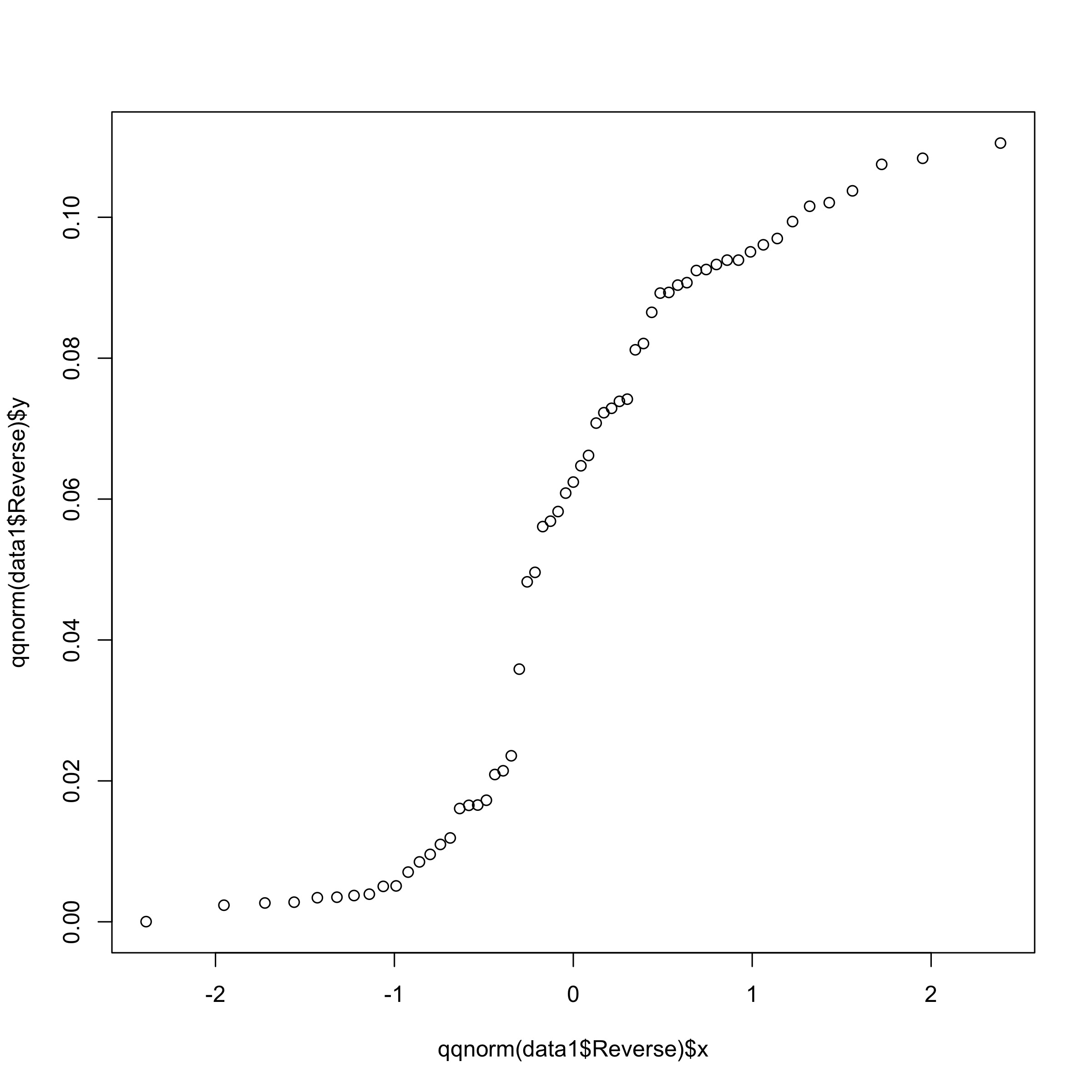

Supplement: Data Sheet 5 — Analysis of best peptide PSM PEP scores. [file DataSheet5.ZIP › Supplementary Data Sheet 5_ Analysis of best peptide PSM PEP scores/PEP_qqnorm/GeneMarkS_database/Reverse_PEP_all.jpeg]

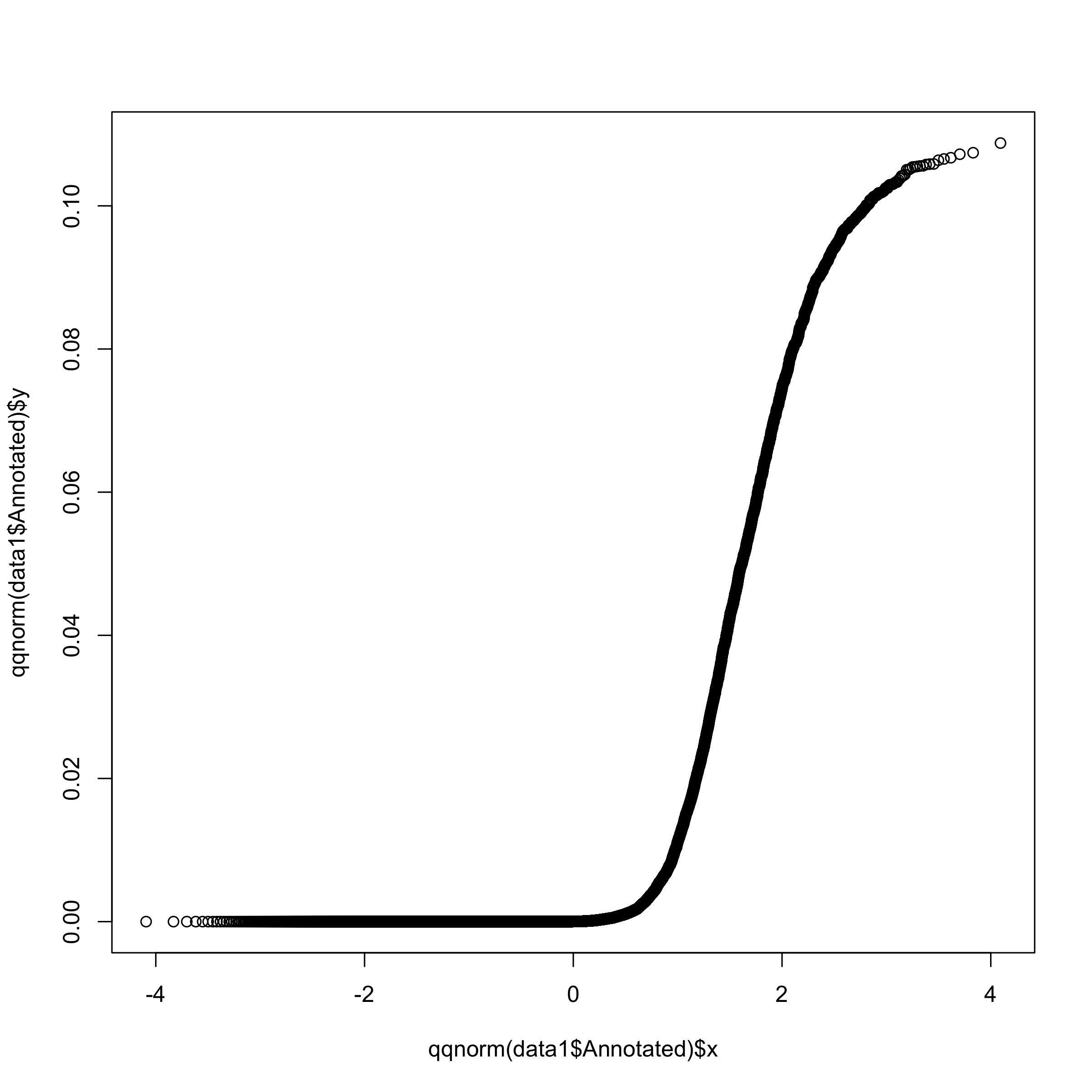

Supplement: Data Sheet 5 — Analysis of best peptide PSM PEP scores. [file DataSheet5.ZIP › Supplementary Data Sheet 5_ Analysis of best peptide PSM PEP scores/PEP_qqnorm/Reference_proteome/Annoted_PEP_2_reps.jpeg]

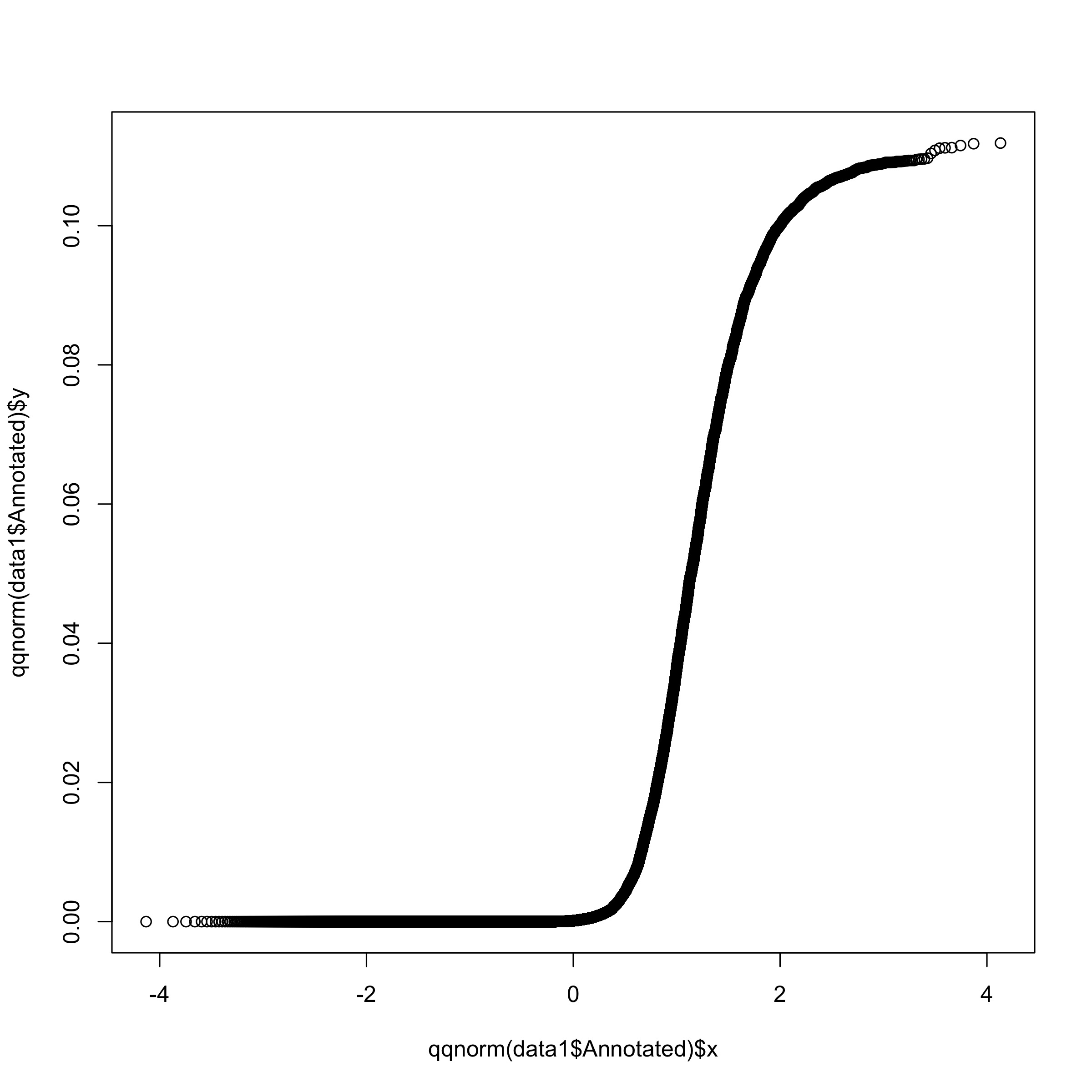

Supplement: Data Sheet 5 — Analysis of best peptide PSM PEP scores. [file DataSheet5.ZIP › Supplementary Data Sheet 5_ Analysis of best peptide PSM PEP scores/PEP_qqnorm/Reference_proteome/Annoted_PEP_all.jpeg]

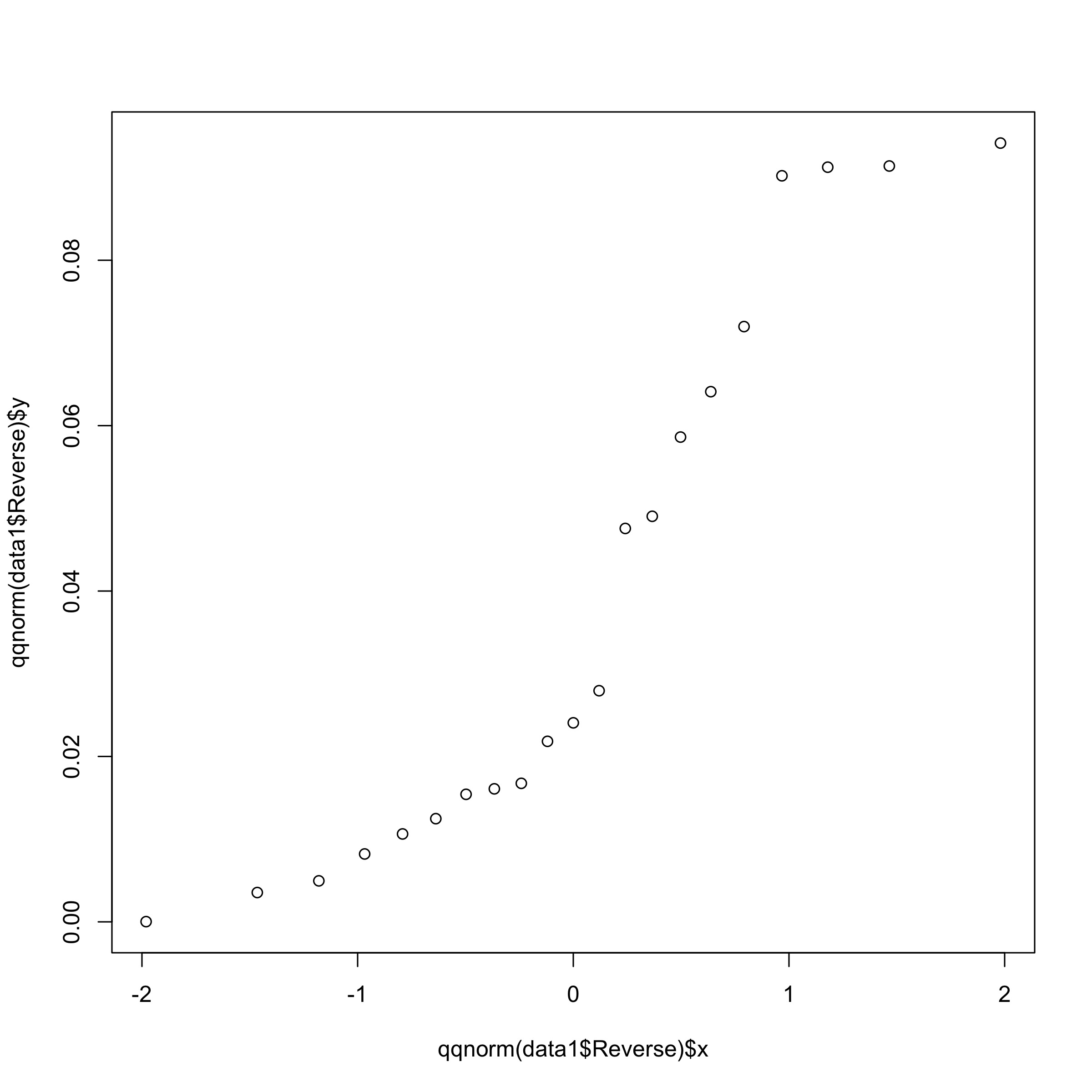

Supplement: Data Sheet 5 — Analysis of best peptide PSM PEP scores. [file DataSheet5.ZIP › Supplementary Data Sheet 5_ Analysis of best peptide PSM PEP scores/PEP_qqnorm/Reference_proteome/Reverse_PEP_2_reps.jpeg]

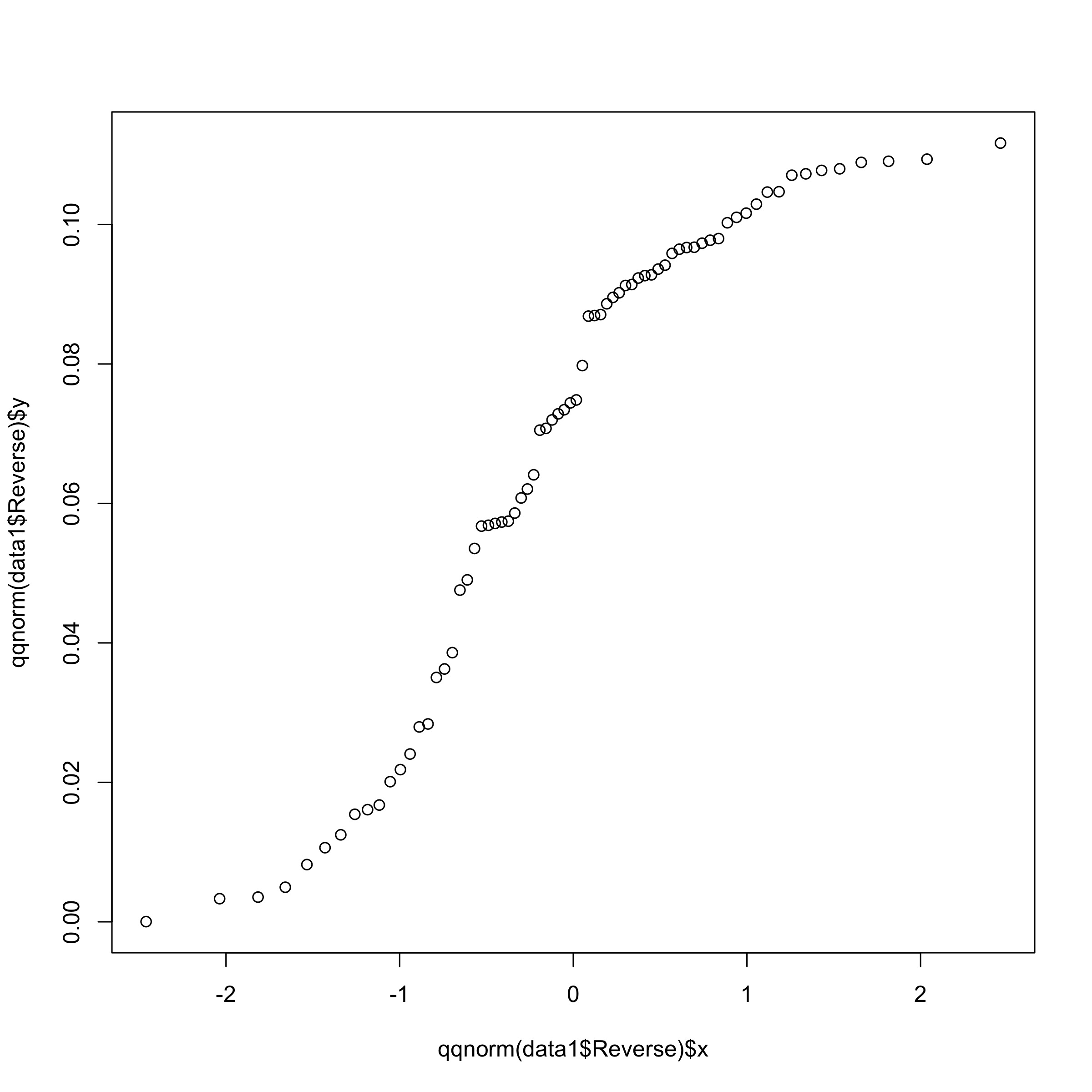

Supplement: Data Sheet 5 — Analysis of best peptide PSM PEP scores. [file DataSheet5.ZIP › Supplementary Data Sheet 5_ Analysis of best peptide PSM PEP scores/PEP_qqnorm/Reference_proteome/Reverse_PEP_all.jpeg]

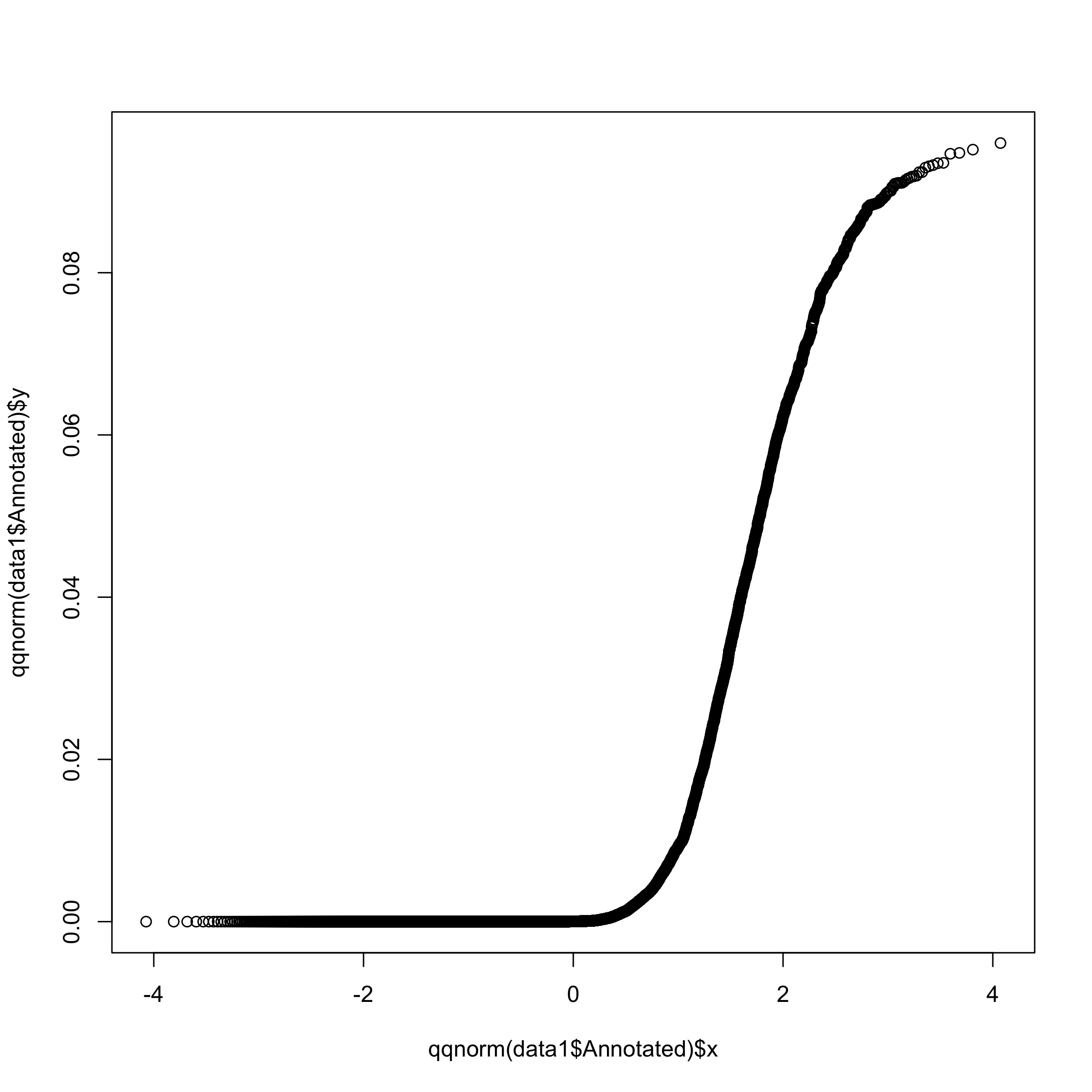

Supplement: Data Sheet 5 — Analysis of best peptide PSM PEP scores. [file DataSheet5.ZIP › Supplementary Data Sheet 5_ Analysis of best peptide PSM PEP scores/PEP_qqnorm/Six_Frame_database/Annoted_PEP_2_reps.jpeg]

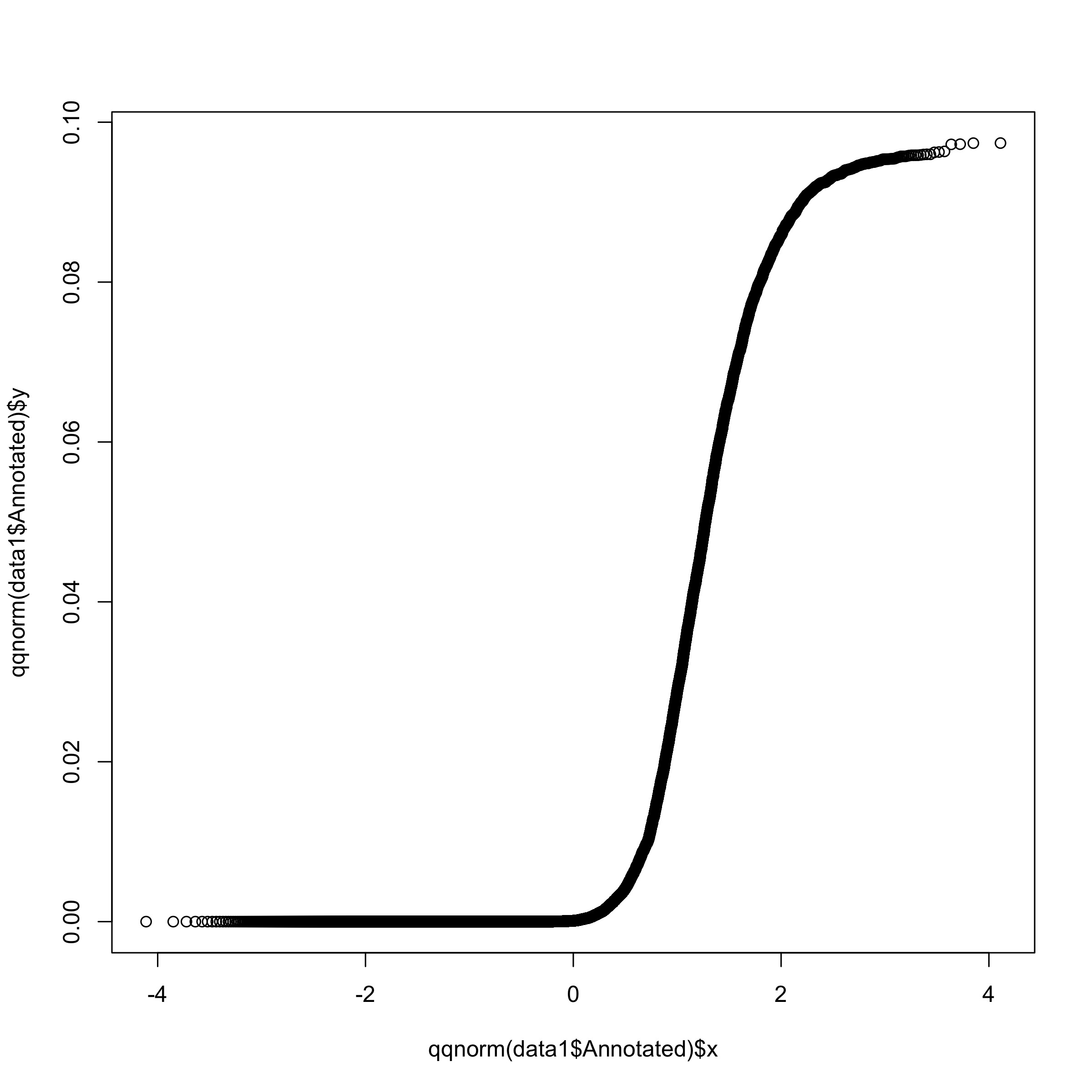

Supplement: Data Sheet 5 — Analysis of best peptide PSM PEP scores. [file DataSheet5.ZIP › Supplementary Data Sheet 5_ Analysis of best peptide PSM PEP scores/PEP_qqnorm/Six_Frame_database/Annoted_PEP_all.jpeg]

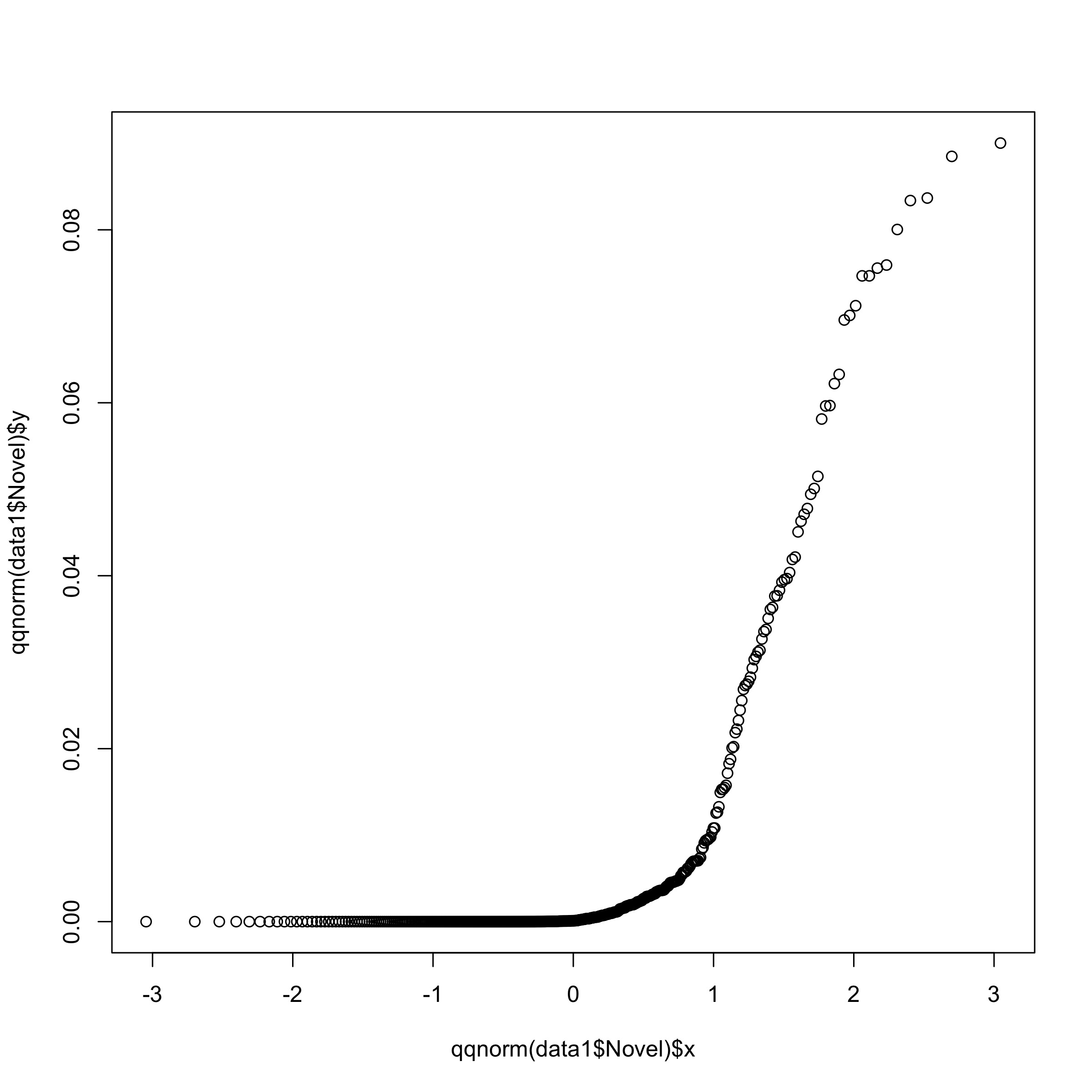

Supplement: Data Sheet 5 — Analysis of best peptide PSM PEP scores. [file DataSheet5.ZIP › Supplementary Data Sheet 5_ Analysis of best peptide PSM PEP scores/PEP_qqnorm/Six_Frame_database/Novel_PEP_2_reps.jpeg]

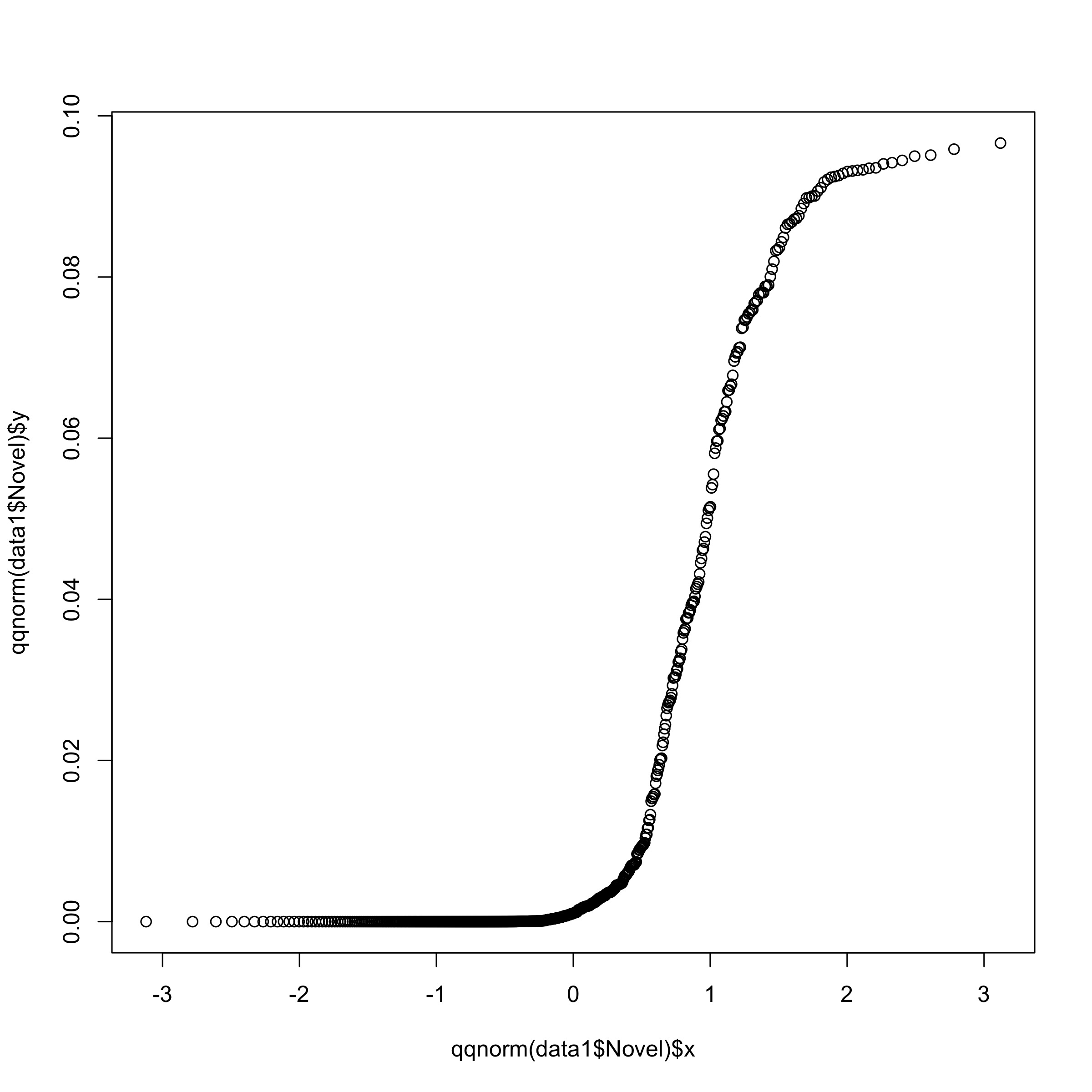

Supplement: Data Sheet 5 — Analysis of best peptide PSM PEP scores. [file DataSheet5.ZIP › Supplementary Data Sheet 5_ Analysis of best peptide PSM PEP scores/PEP_qqnorm/Six_Frame_database/Novel_PEP_all.jpeg]

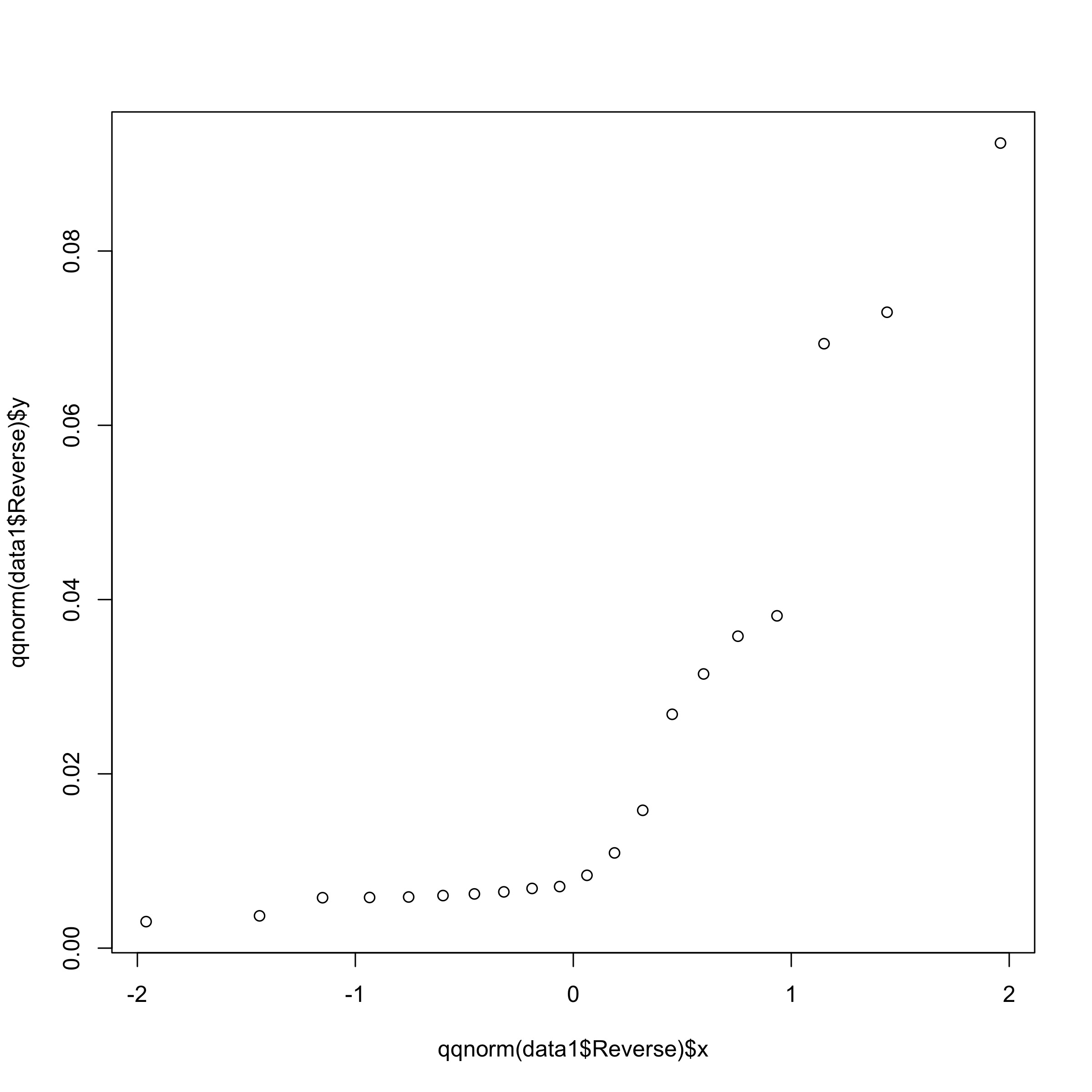

Supplement: Data Sheet 5 — Analysis of best peptide PSM PEP scores. [file DataSheet5.ZIP › Supplementary Data Sheet 5_ Analysis of best peptide PSM PEP scores/PEP_qqnorm/Six_Frame_database/Reverse_PEP_2_reps.jpeg]

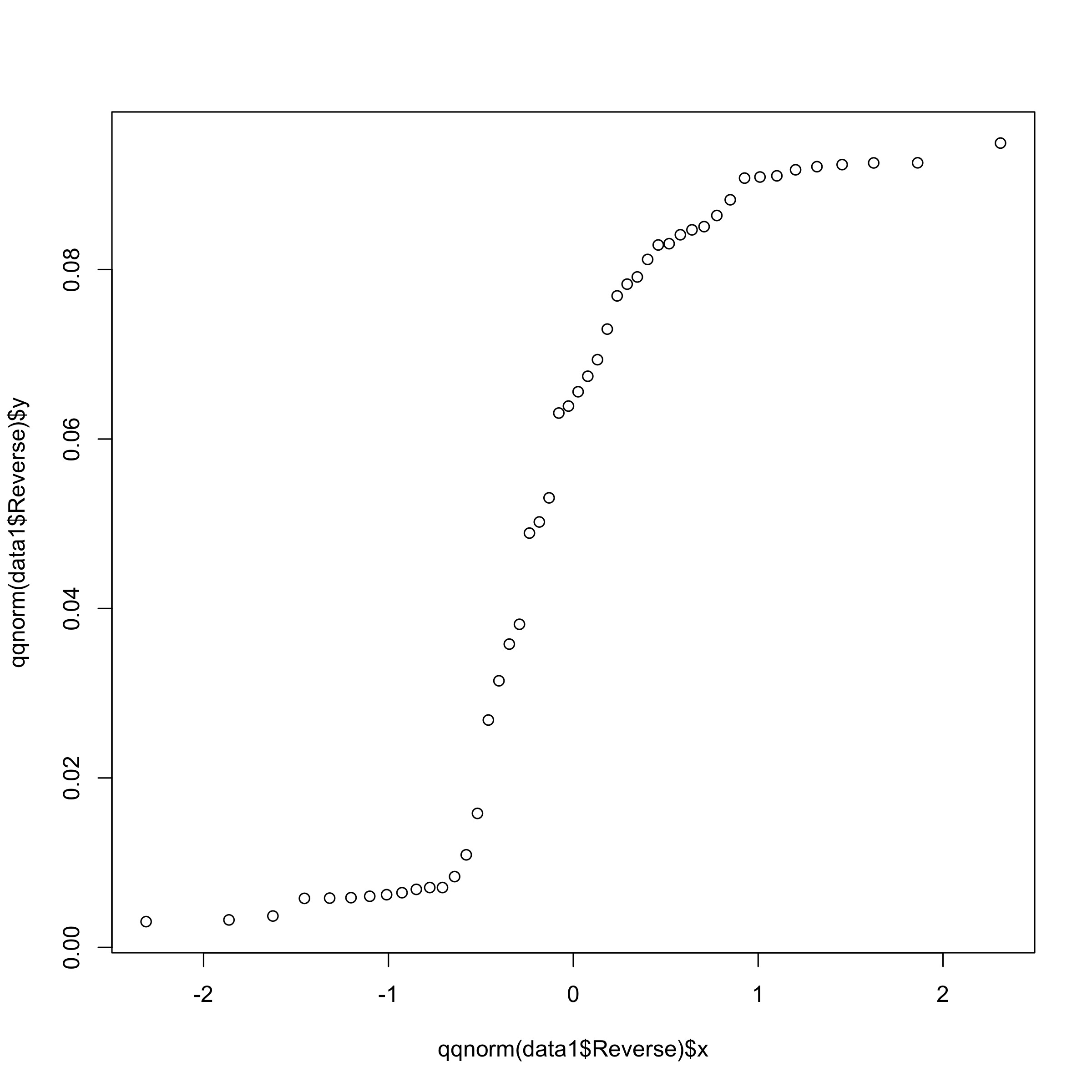

Supplement: Data Sheet 5 — Analysis of best peptide PSM PEP scores. [file DataSheet5.ZIP › Supplementary Data Sheet 5_ Analysis of best peptide PSM PEP scores/PEP_qqnorm/Six_Frame_database/Reverse_PEP_all.jpeg]

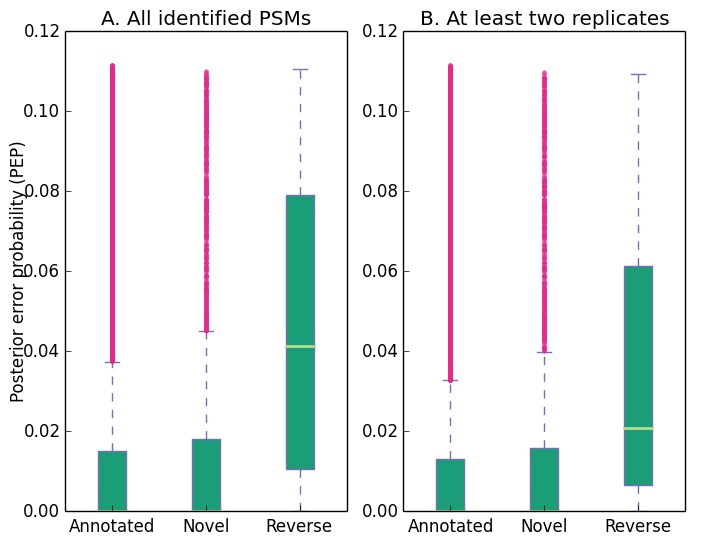

Supplement: Data Sheet 6 — Analysis of all peptide PSM PEP scores. [file DataSheet6.ZIP › Supplementary Data Sheet 6_ Analysis of all peptide PSM PEP scores/PEP_boxplots/GeneMarkS database PSM PEP.jpg]

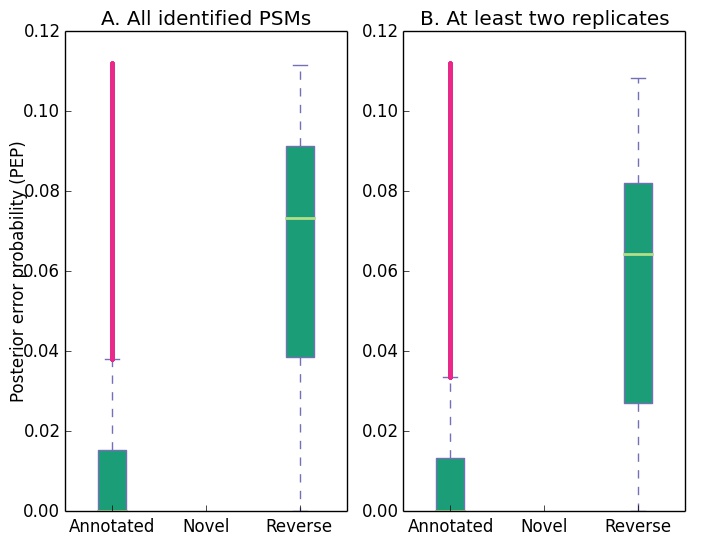

Supplement: Data Sheet 6 — Analysis of all peptide PSM PEP scores. [file DataSheet6.ZIP › Supplementary Data Sheet 6_ Analysis of all peptide PSM PEP scores/PEP_boxplots/Reference proteome PSM PEP.jpg]

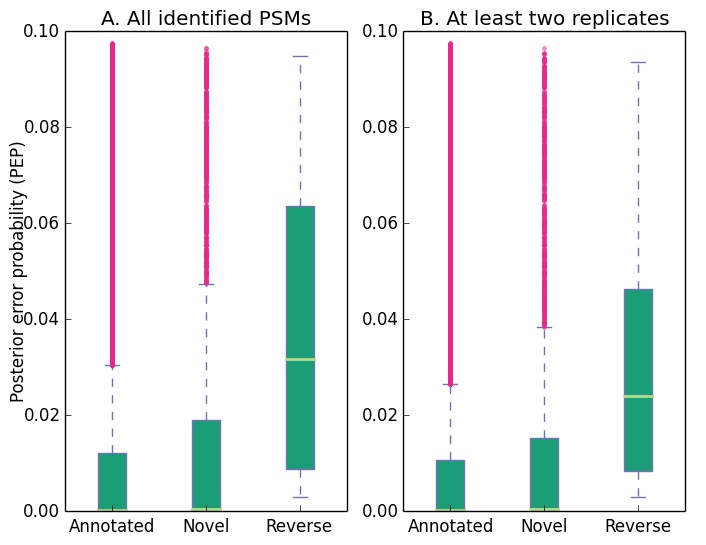

Supplement: Data Sheet 6 — Analysis of all peptide PSM PEP scores. [file DataSheet6.ZIP › Supplementary Data Sheet 6_ Analysis of all peptide PSM PEP scores/PEP_boxplots/Six Frame database PSM PEP.jpg]

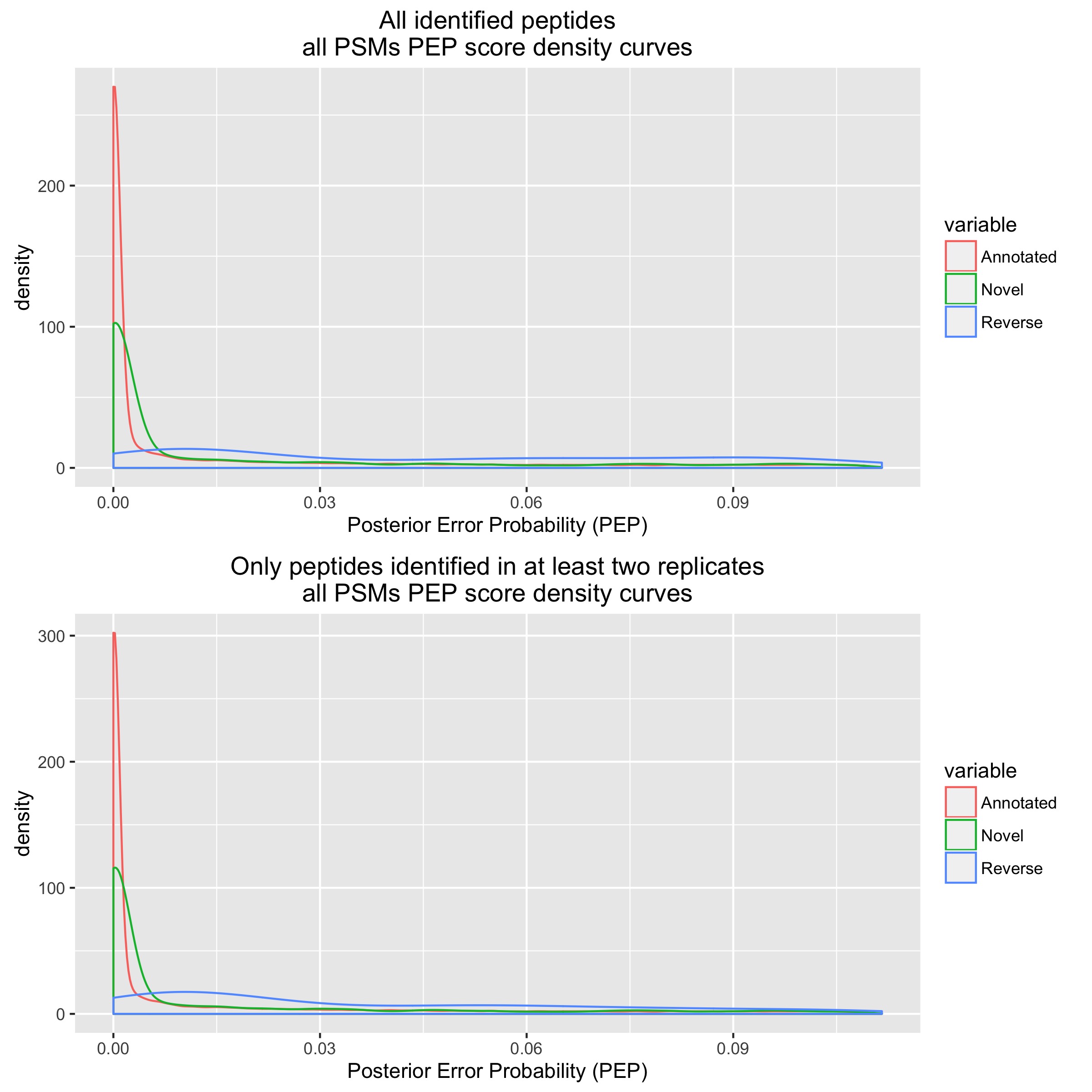

Supplement: Data Sheet 6 — Analysis of all peptide PSM PEP scores. [file DataSheet6.ZIP › Supplementary Data Sheet 6_ Analysis of all peptide PSM PEP scores/PEP_density/GeneMarkS_database_MSMS_PEP_score_density.jpeg]

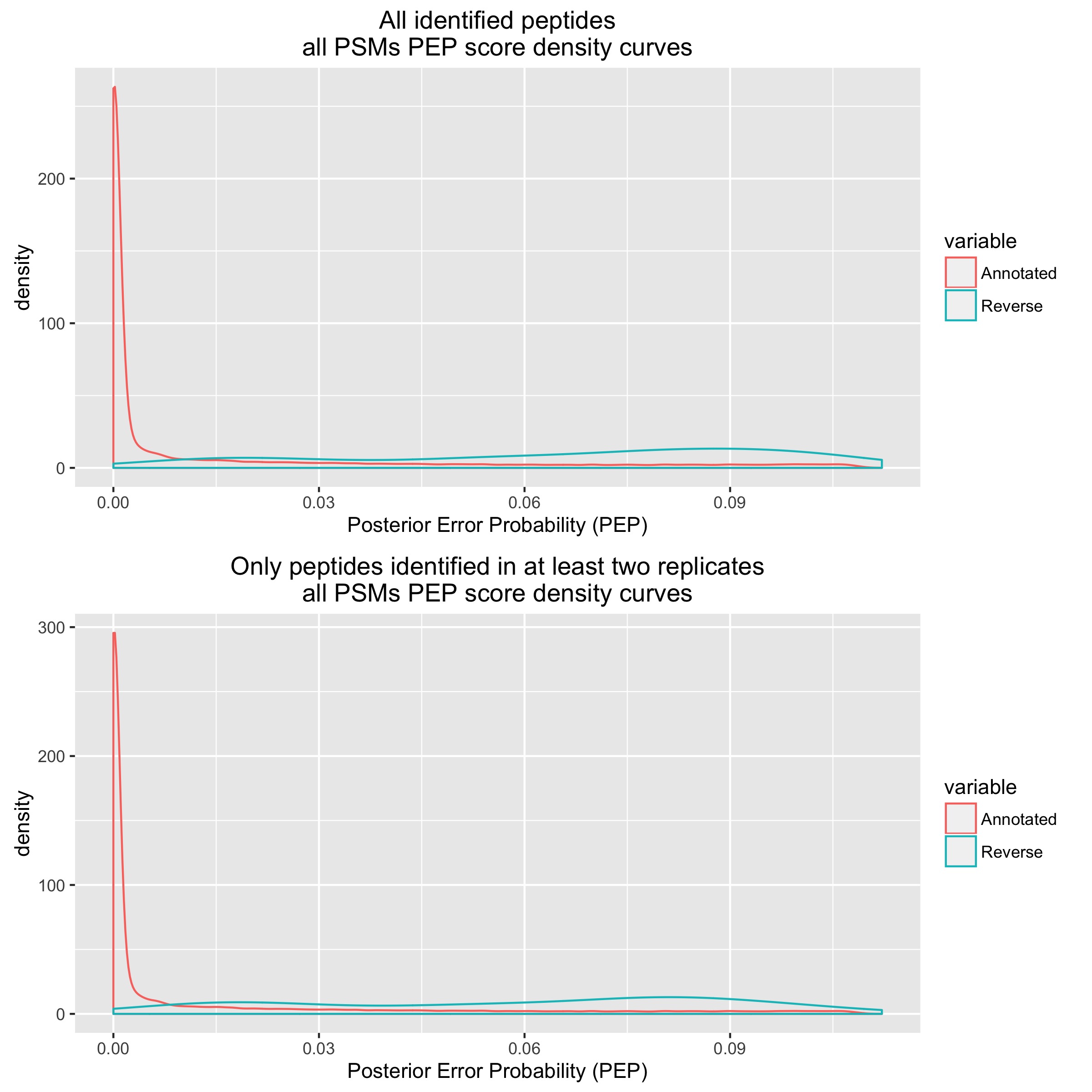

Supplement: Data Sheet 6 — Analysis of all peptide PSM PEP scores. [file DataSheet6.ZIP › Supplementary Data Sheet 6_ Analysis of all peptide PSM PEP scores/PEP_density/Reference_proteome_MSMS_PEP_score_density.jpeg]

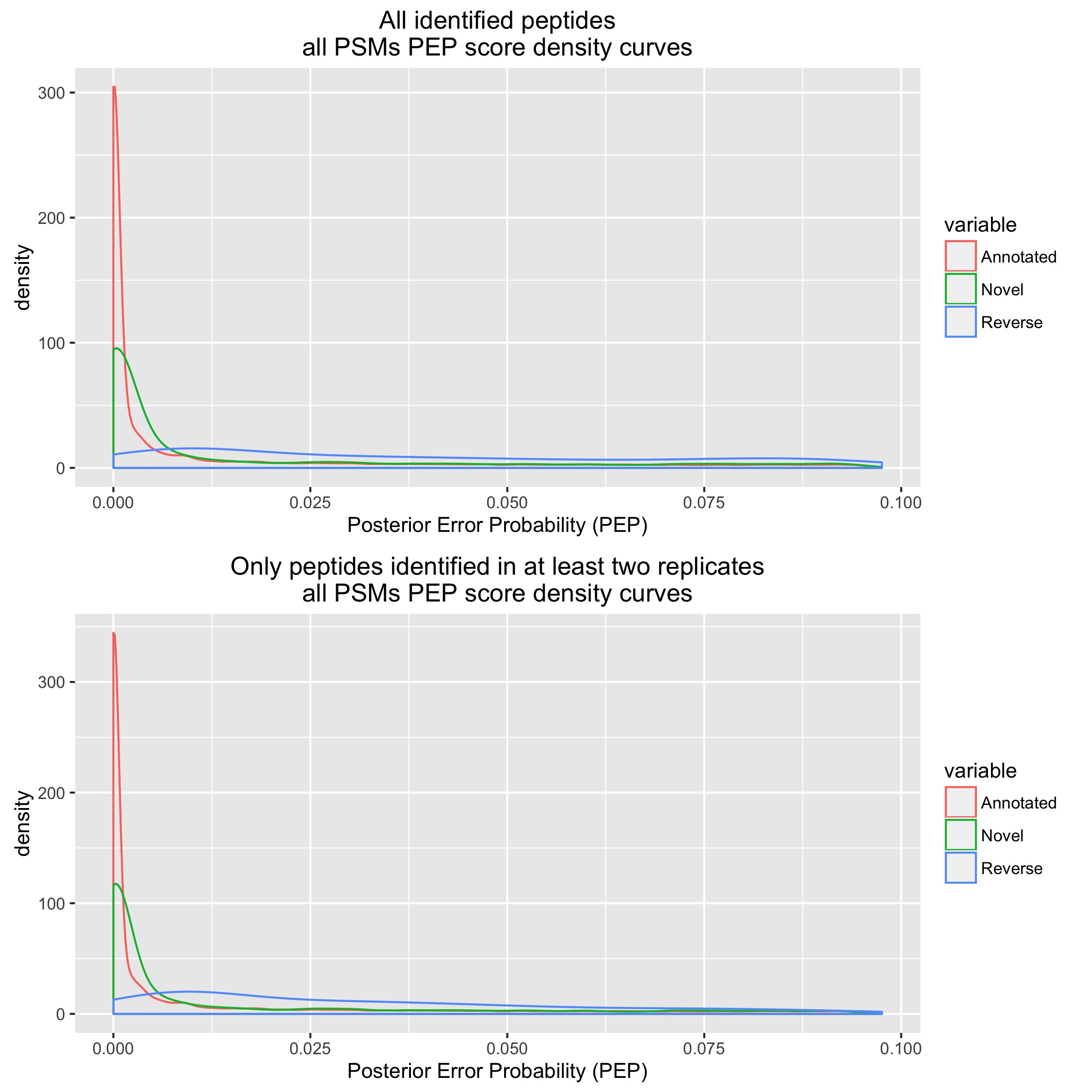

Supplement: Data Sheet 6 — Analysis of all peptide PSM PEP scores. [file DataSheet6.ZIP › Supplementary Data Sheet 6_ Analysis of all peptide PSM PEP scores/PEP_density/Six_Frame_database_MSMS_PEP_score_density.jpeg]

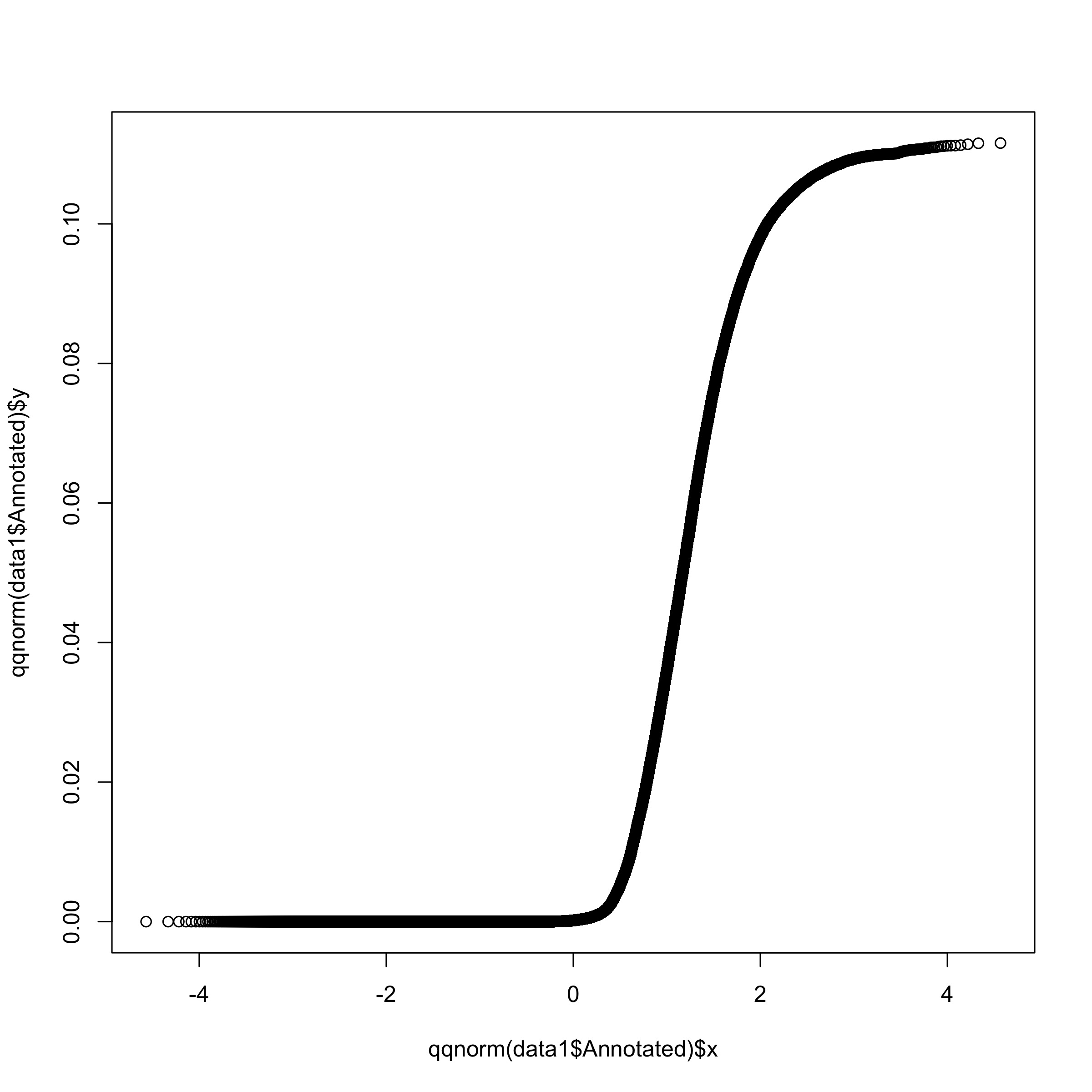

Supplement: Data Sheet 6 — Analysis of all peptide PSM PEP scores. [file DataSheet6.ZIP › Supplementary Data Sheet 6_ Analysis of all peptide PSM PEP scores/PEP_qqnorm/GeneMarkS_database/Annoted_PEP_2_reps.jpeg]

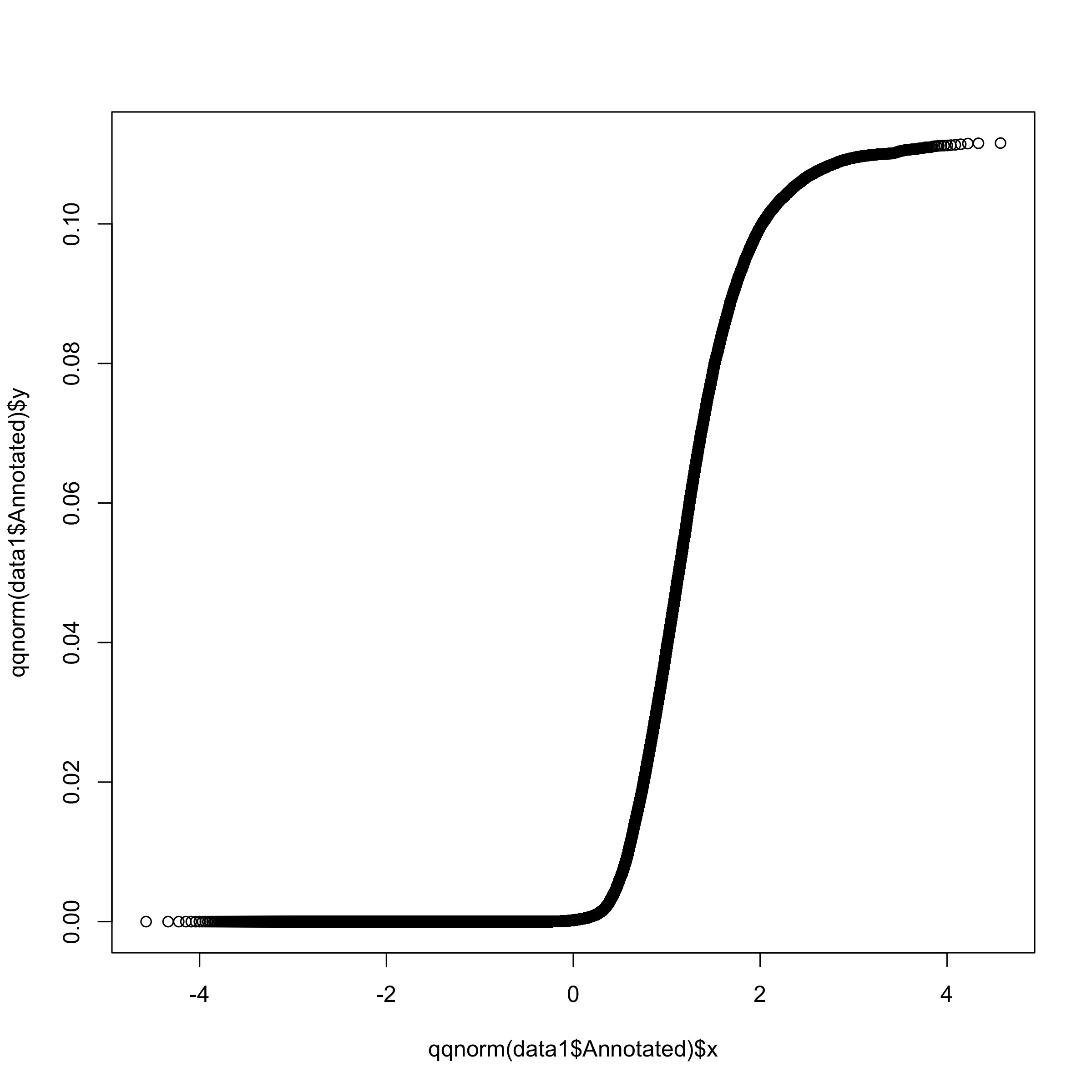

Supplement: Data Sheet 6 — Analysis of all peptide PSM PEP scores. [file DataSheet6.ZIP › Supplementary Data Sheet 6_ Analysis of all peptide PSM PEP scores/PEP_qqnorm/GeneMarkS_database/Annoted_PEP_all.jpeg]

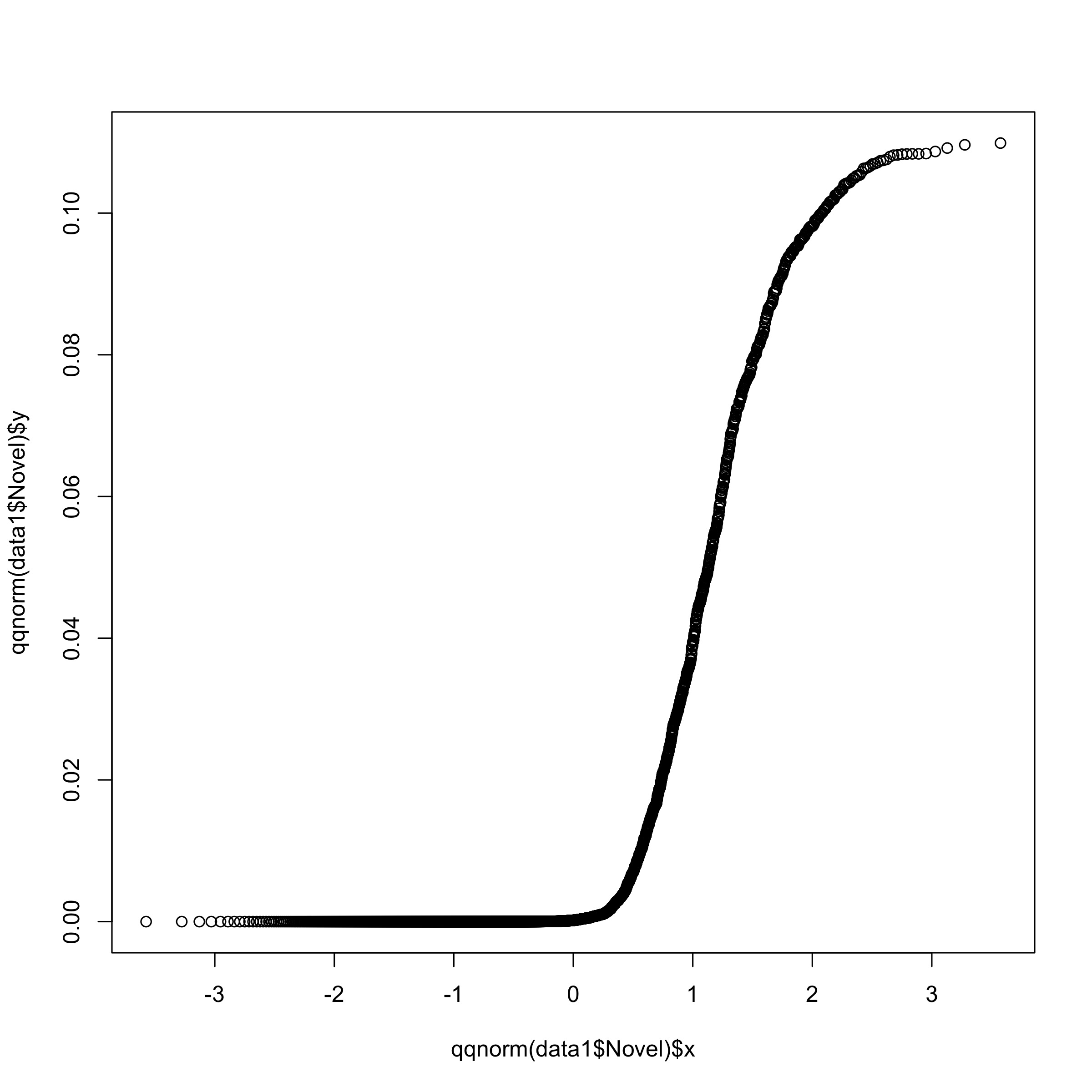

Supplement: Data Sheet 6 — Analysis of all peptide PSM PEP scores. [file DataSheet6.ZIP › Supplementary Data Sheet 6_ Analysis of all peptide PSM PEP scores/PEP_qqnorm/GeneMarkS_database/Novel_PEP_2_reps.jpeg]

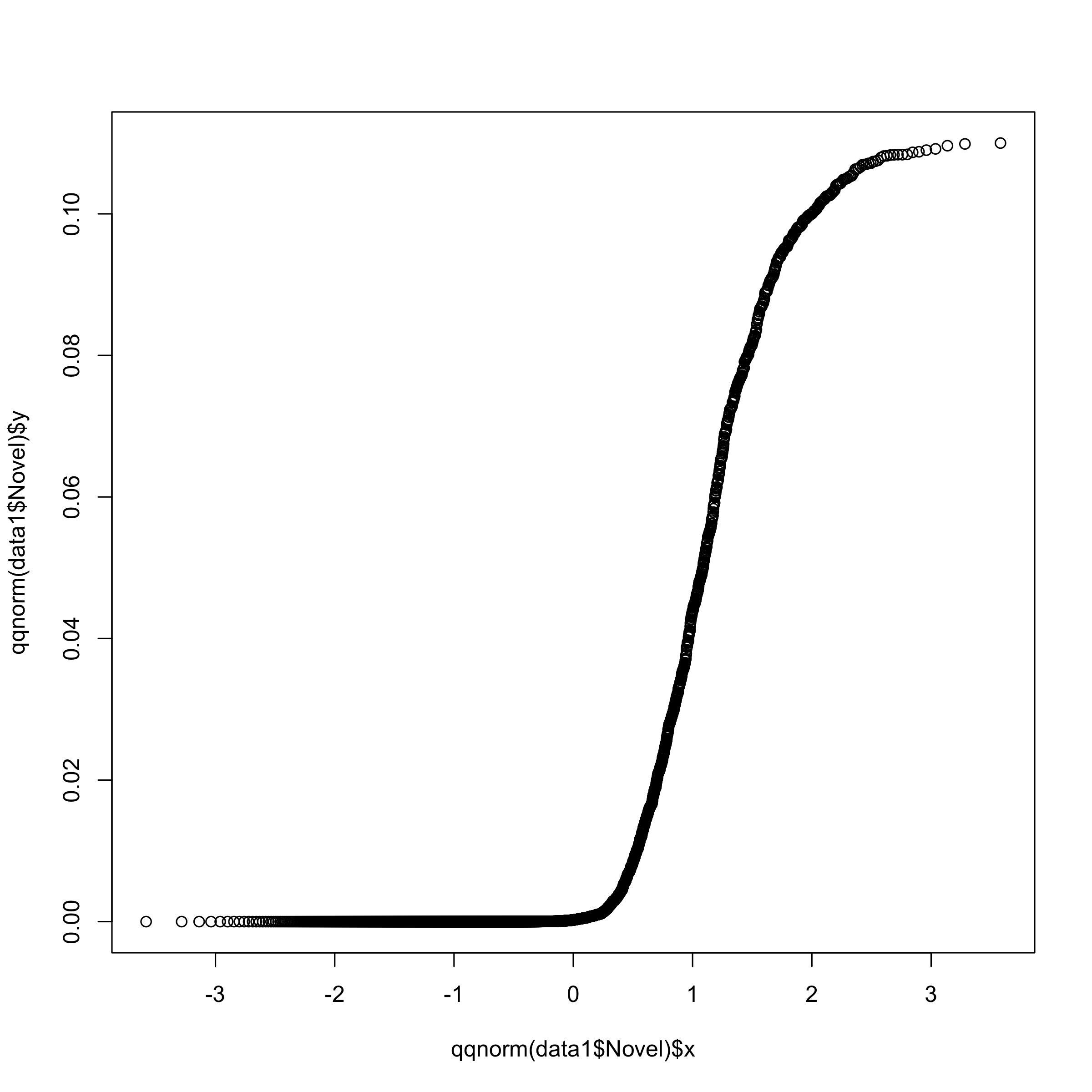

Supplement: Data Sheet 6 — Analysis of all peptide PSM PEP scores. [file DataSheet6.ZIP › Supplementary Data Sheet 6_ Analysis of all peptide PSM PEP scores/PEP_qqnorm/GeneMarkS_database/Novel_PEP_all.jpeg]

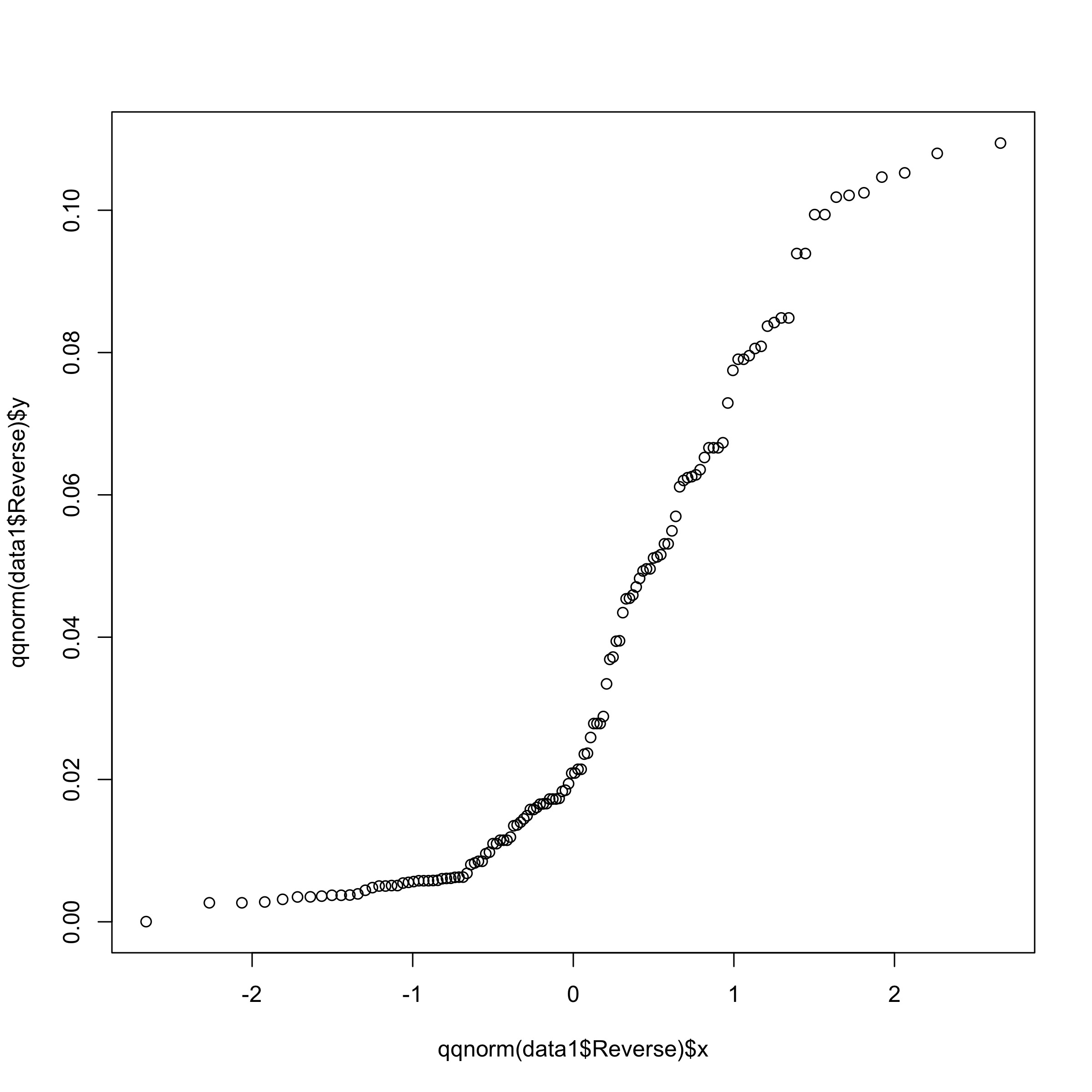

Supplement: Data Sheet 6 — Analysis of all peptide PSM PEP scores. [file DataSheet6.ZIP › Supplementary Data Sheet 6_ Analysis of all peptide PSM PEP scores/PEP_qqnorm/GeneMarkS_database/Reverse_PEP_2_reps.jpeg]

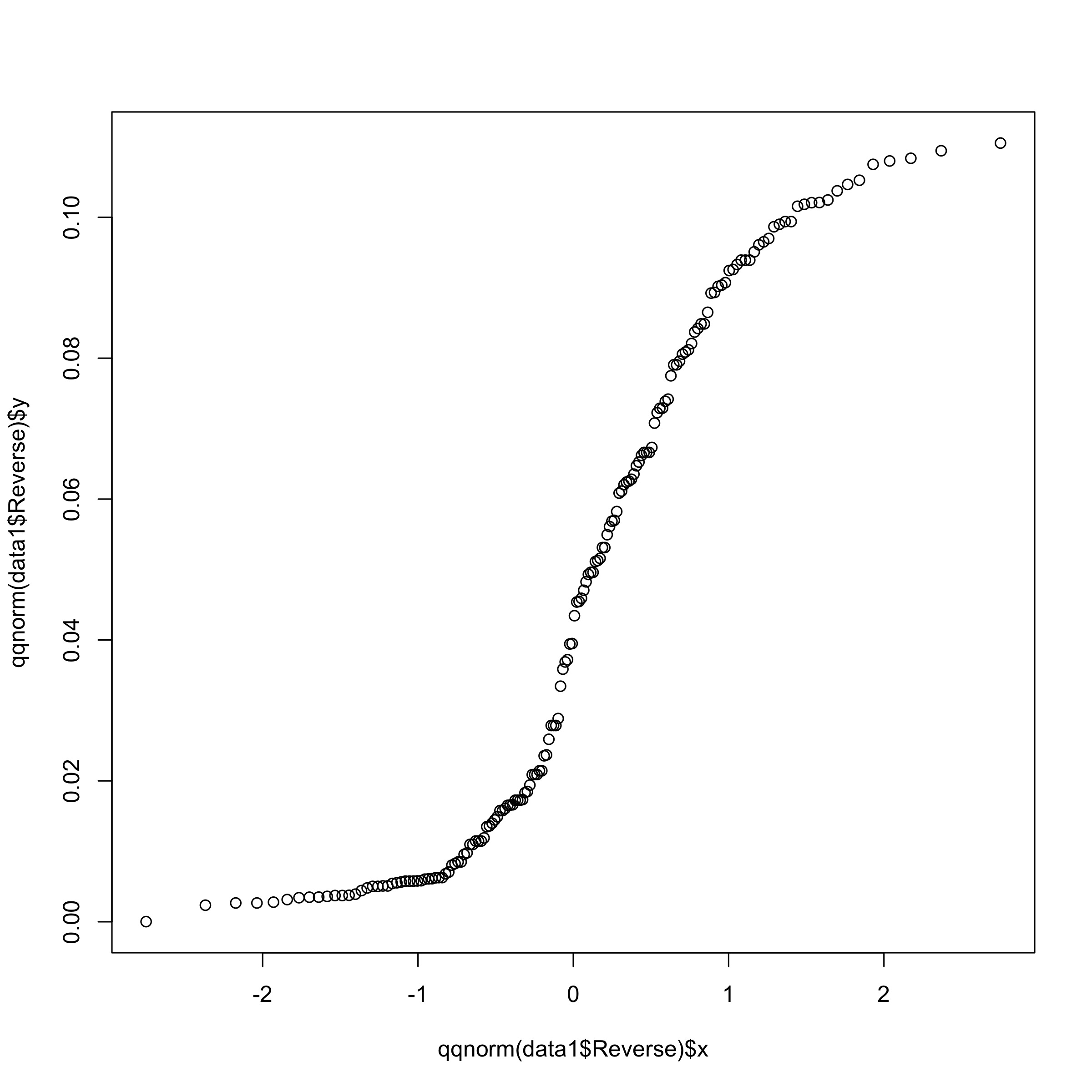

Supplement: Data Sheet 6 — Analysis of all peptide PSM PEP scores. [file DataSheet6.ZIP › Supplementary Data Sheet 6_ Analysis of all peptide PSM PEP scores/PEP_qqnorm/GeneMarkS_database/Reverse_PEP_all.jpeg]

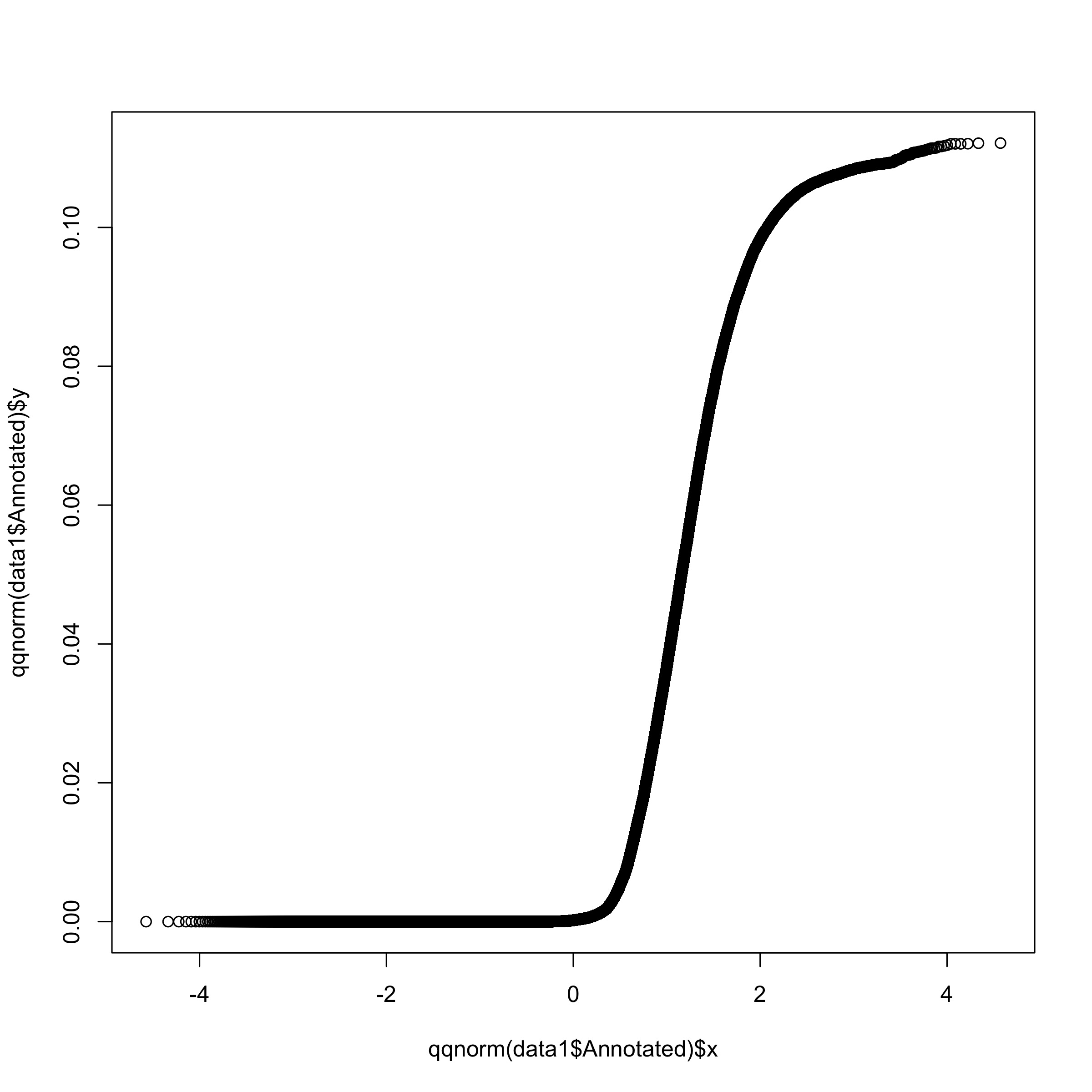

Supplement: Data Sheet 6 — Analysis of all peptide PSM PEP scores. [file DataSheet6.ZIP › Supplementary Data Sheet 6_ Analysis of all peptide PSM PEP scores/PEP_qqnorm/Reference_proteome/Annoted_PEP_2_reps.jpeg]

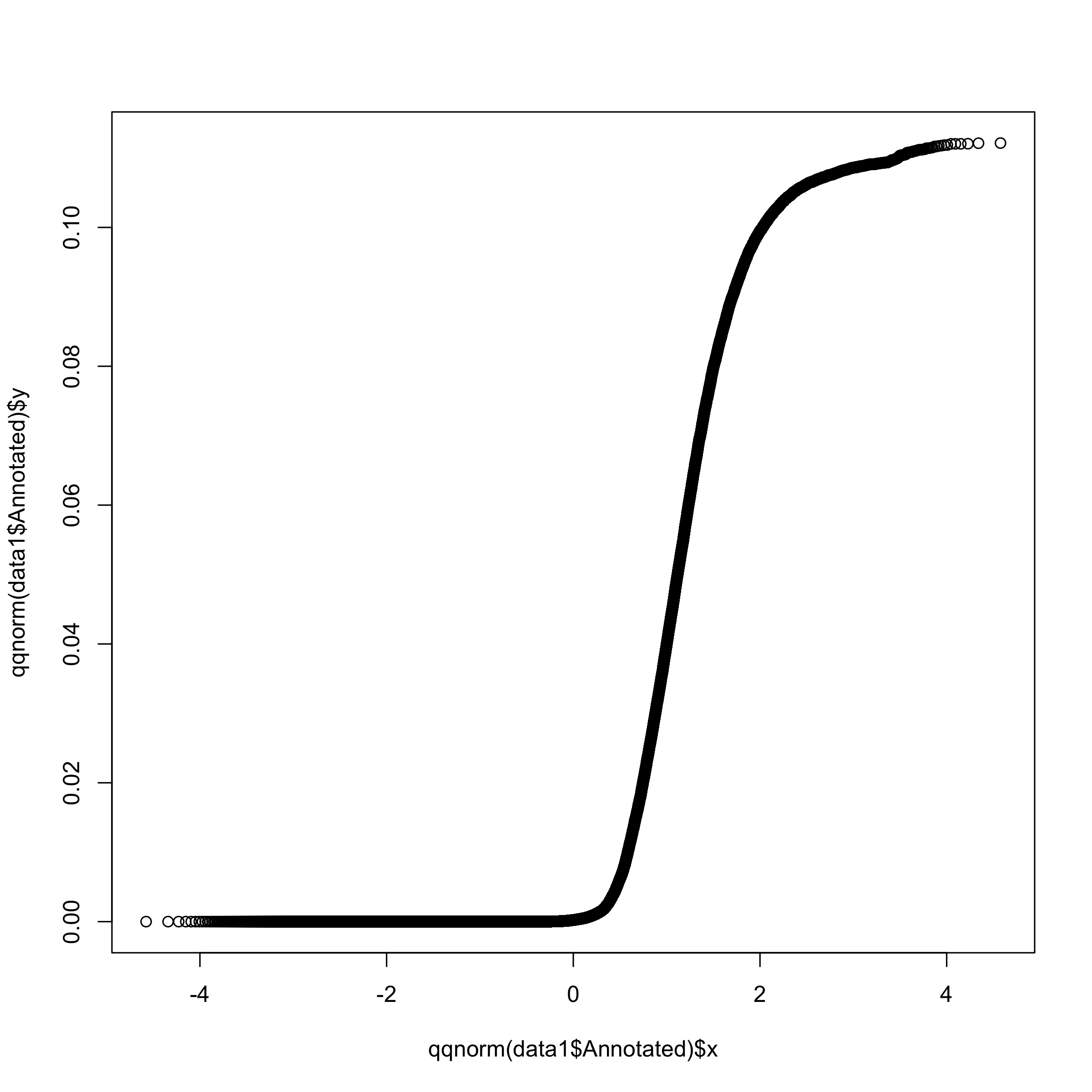

Supplement: Data Sheet 6 — Analysis of all peptide PSM PEP scores. [file DataSheet6.ZIP › Supplementary Data Sheet 6_ Analysis of all peptide PSM PEP scores/PEP_qqnorm/Reference_proteome/Annoted_PEP_all.jpeg]

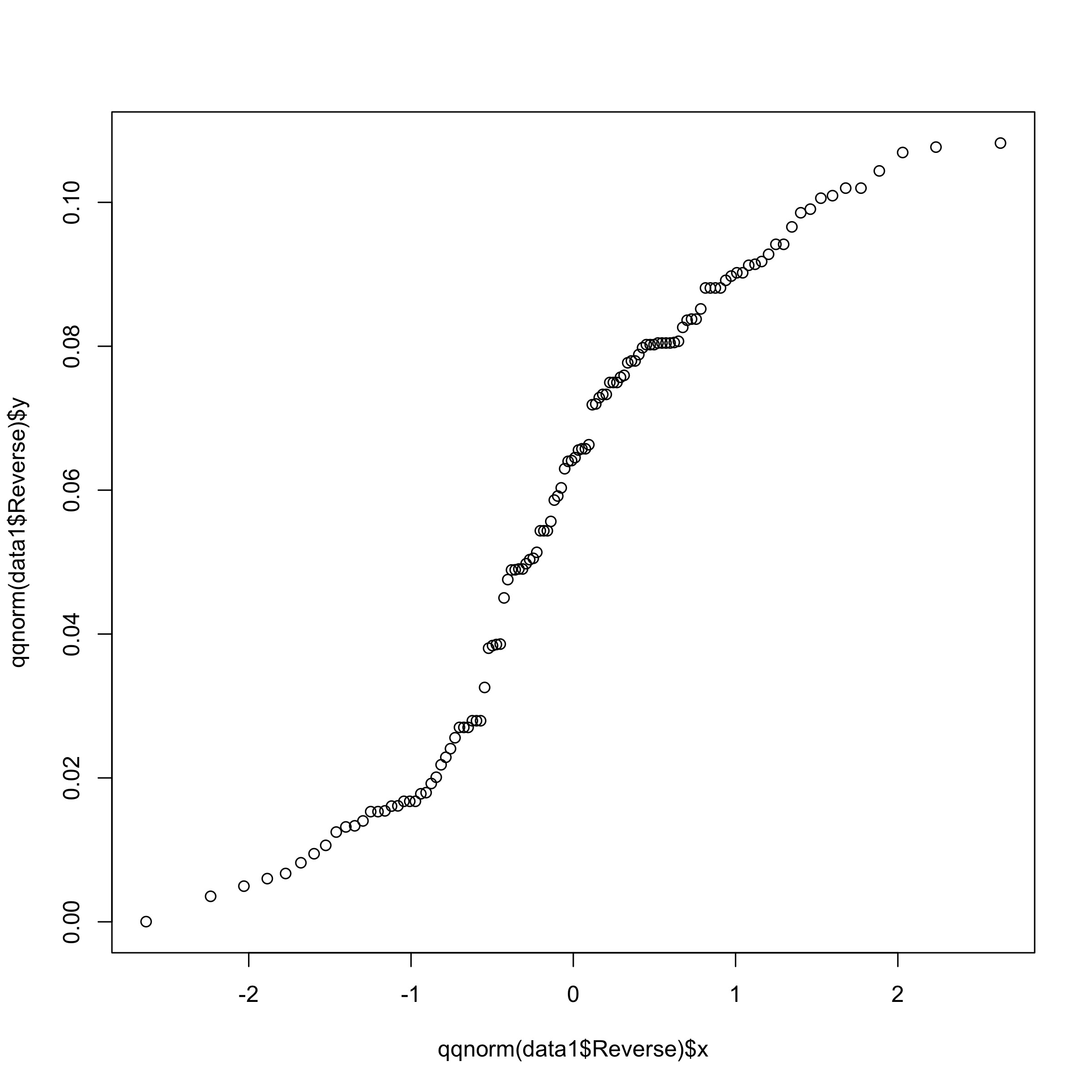

Supplement: Data Sheet 6 — Analysis of all peptide PSM PEP scores. [file DataSheet6.ZIP › Supplementary Data Sheet 6_ Analysis of all peptide PSM PEP scores/PEP_qqnorm/Reference_proteome/Reverse_PEP_2_reps.jpeg]

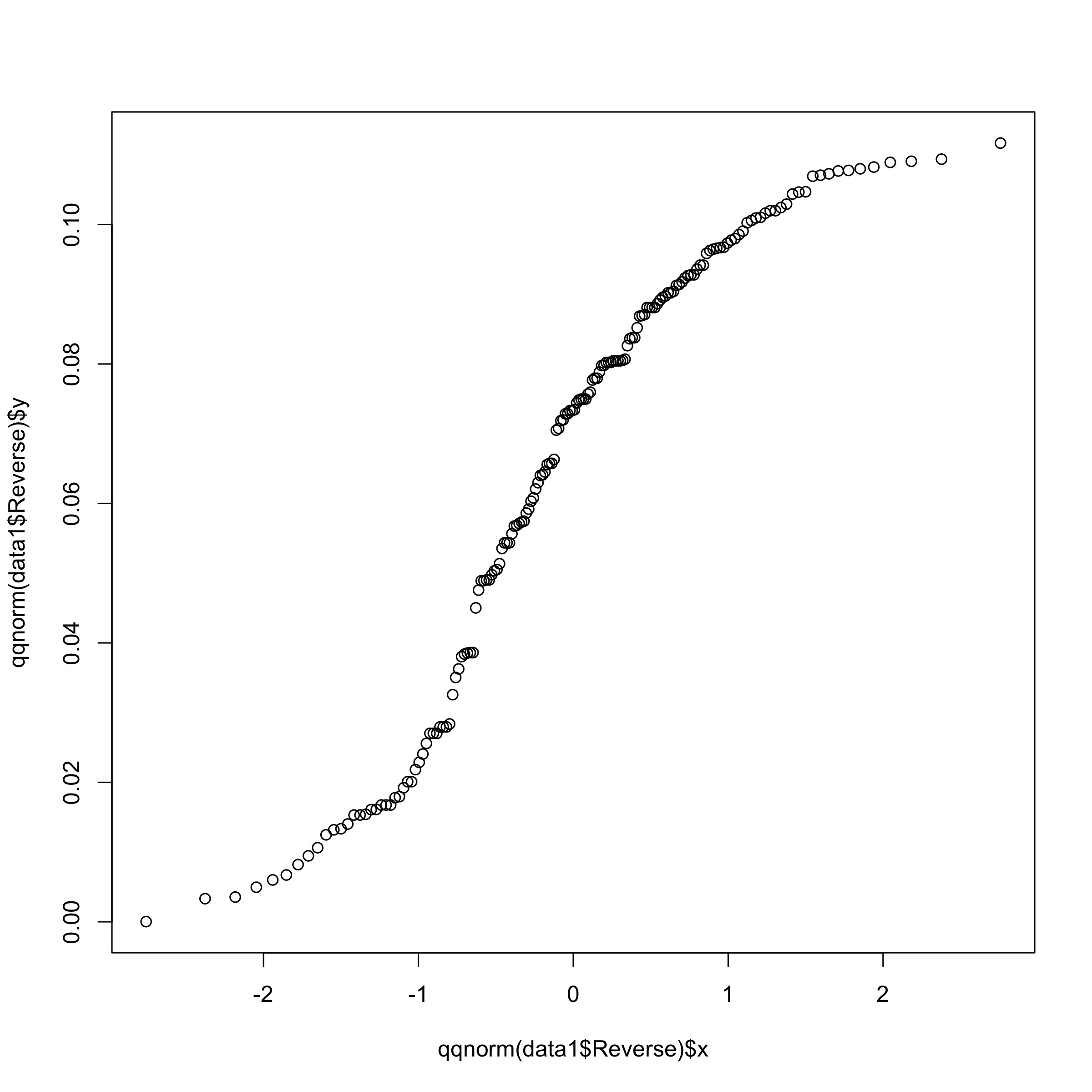

Supplement: Data Sheet 6 — Analysis of all peptide PSM PEP scores. [file DataSheet6.ZIP › Supplementary Data Sheet 6_ Analysis of all peptide PSM PEP scores/PEP_qqnorm/Reference_proteome/Reverse_PEP_all.jpeg]

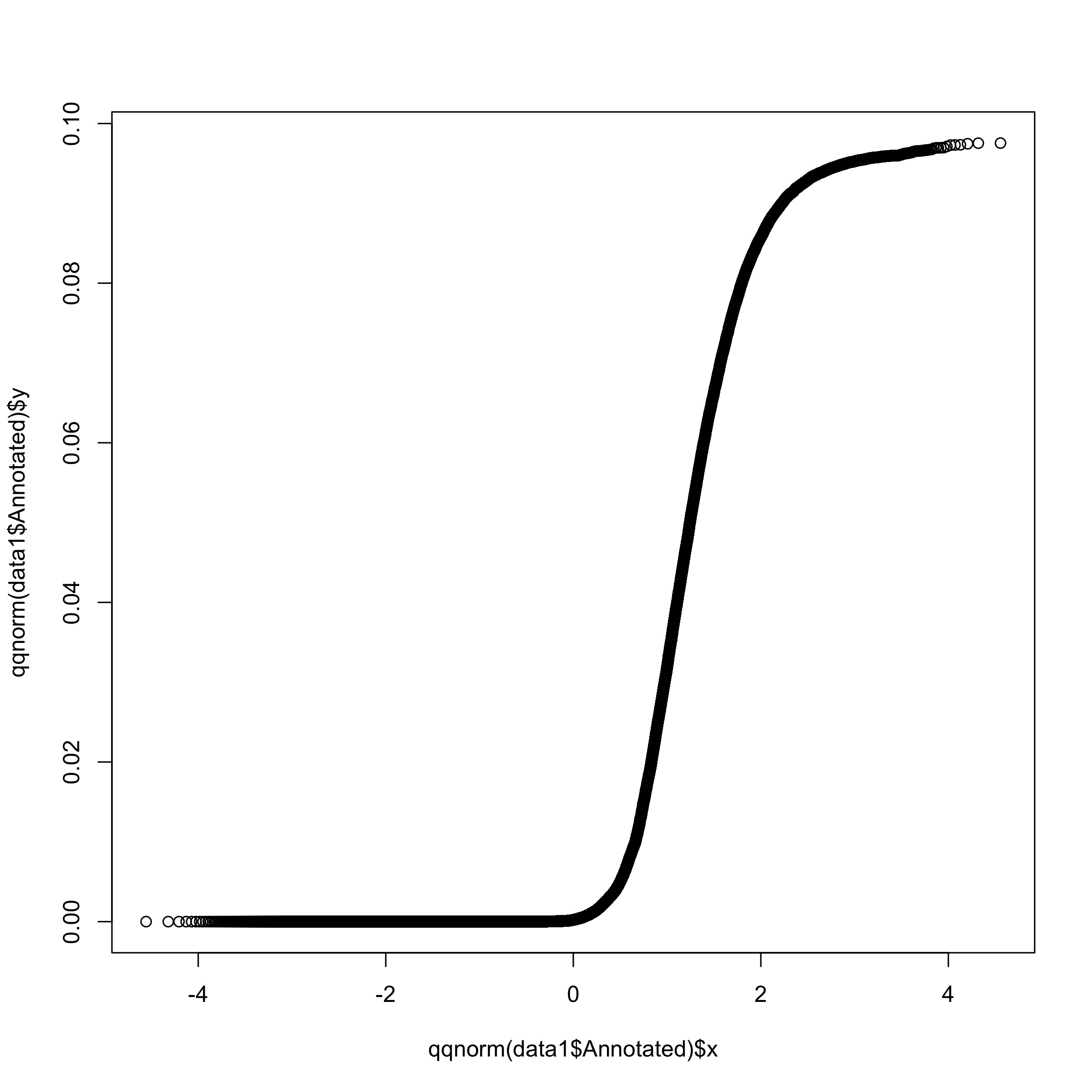

Supplement: Data Sheet 6 — Analysis of all peptide PSM PEP scores. [file DataSheet6.ZIP › Supplementary Data Sheet 6_ Analysis of all peptide PSM PEP scores/PEP_qqnorm/Six_Frame_database/Annoted_PEP_2_reps.jpeg]

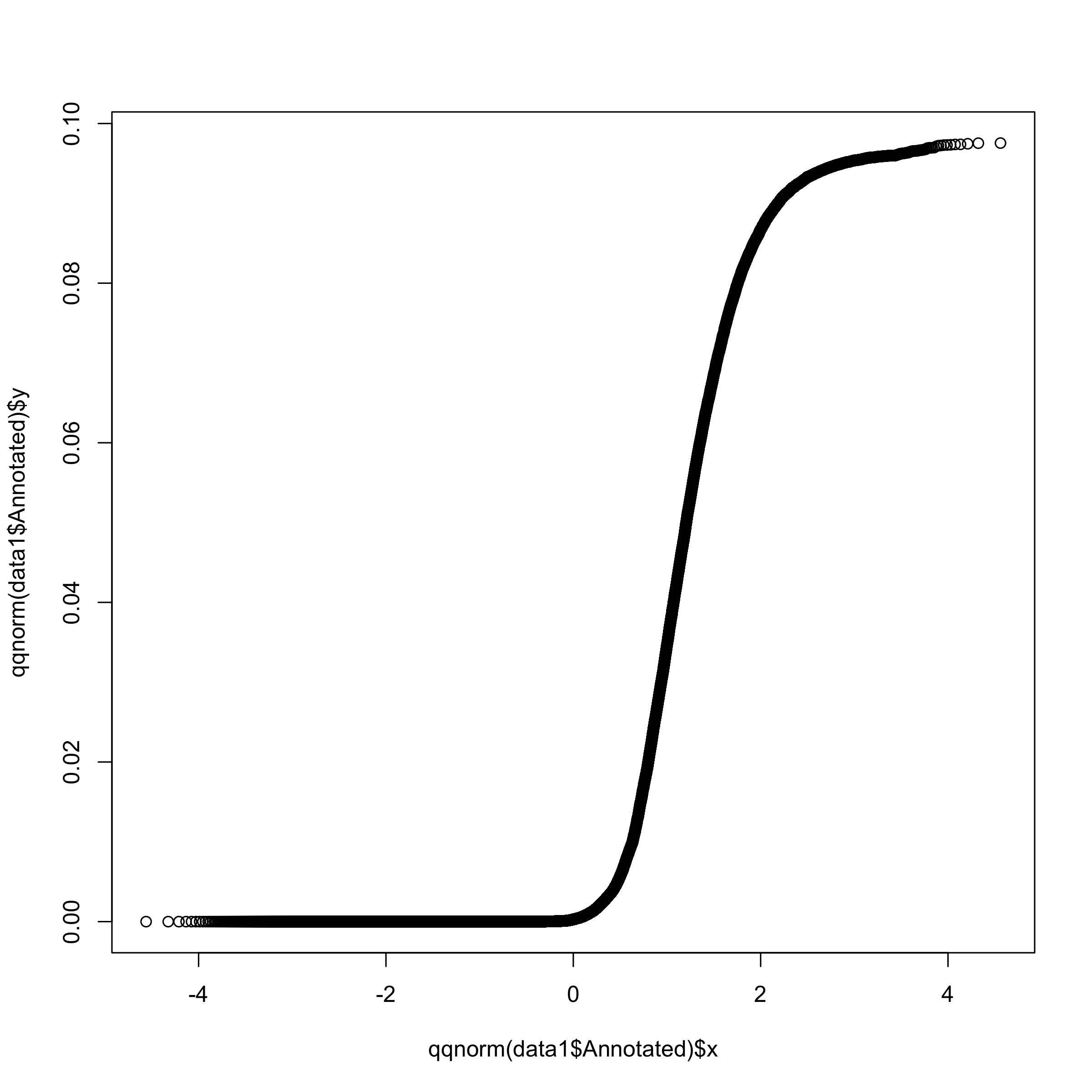

Supplement: Data Sheet 6 — Analysis of all peptide PSM PEP scores. [file DataSheet6.ZIP › Supplementary Data Sheet 6_ Analysis of all peptide PSM PEP scores/PEP_qqnorm/Six_Frame_database/Annoted_PEP_all.jpeg]

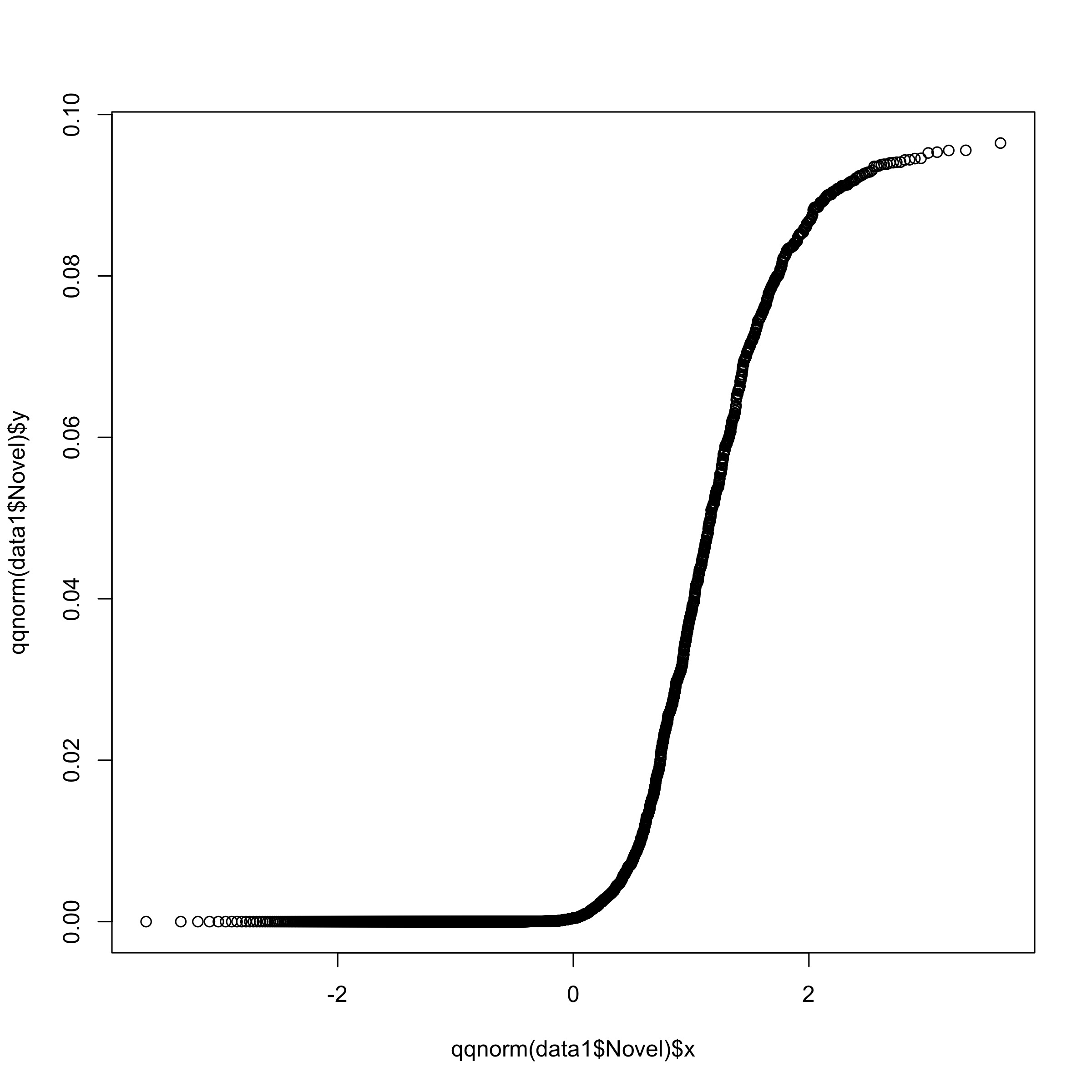

Supplement: Data Sheet 6 — Analysis of all peptide PSM PEP scores. [file DataSheet6.ZIP › Supplementary Data Sheet 6_ Analysis of all peptide PSM PEP scores/PEP_qqnorm/Six_Frame_database/Novel_PEP_2_reps.jpeg]

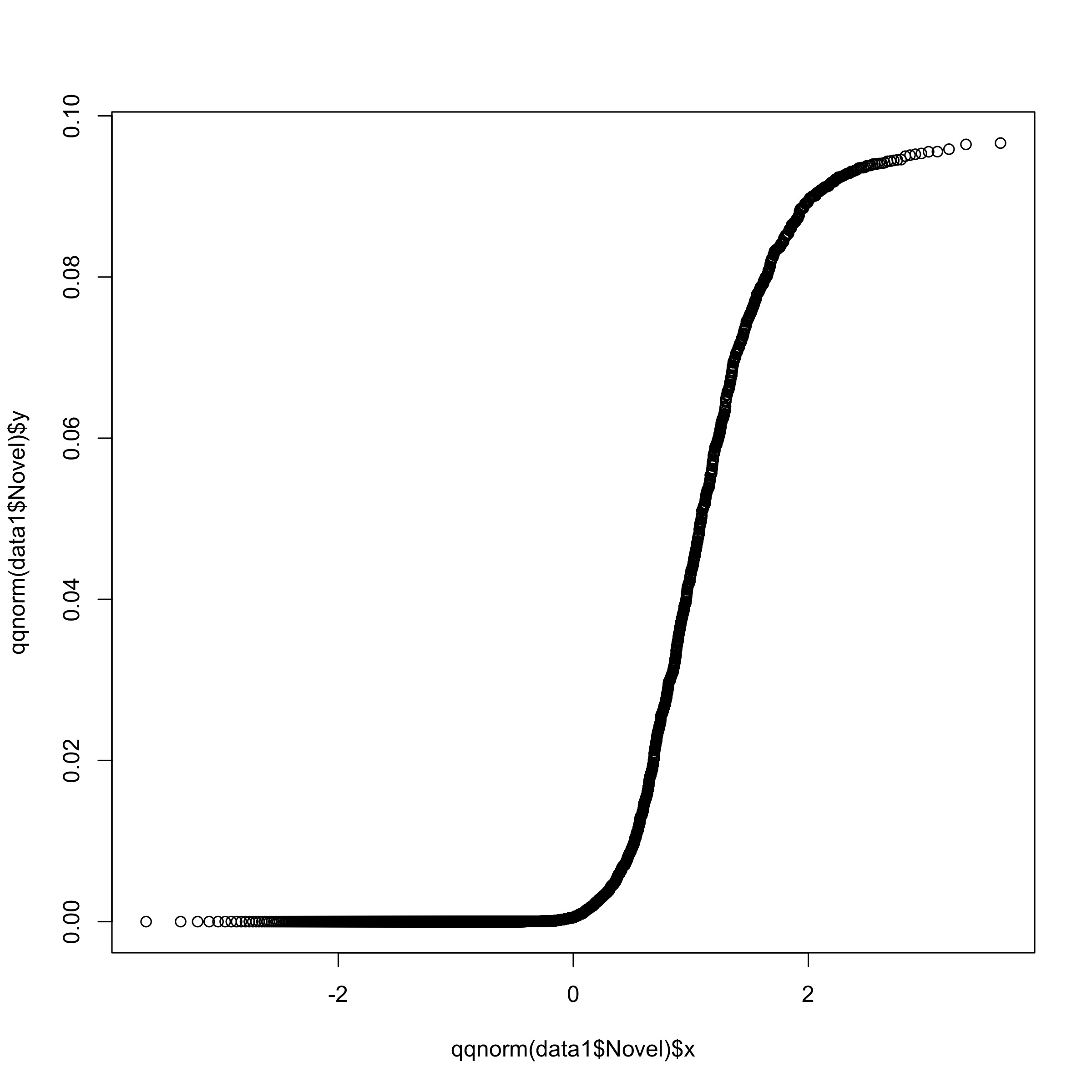

Supplement: Data Sheet 6 — Analysis of all peptide PSM PEP scores. [file DataSheet6.ZIP › Supplementary Data Sheet 6_ Analysis of all peptide PSM PEP scores/PEP_qqnorm/Six_Frame_database/Novel_PEP_all.jpeg]

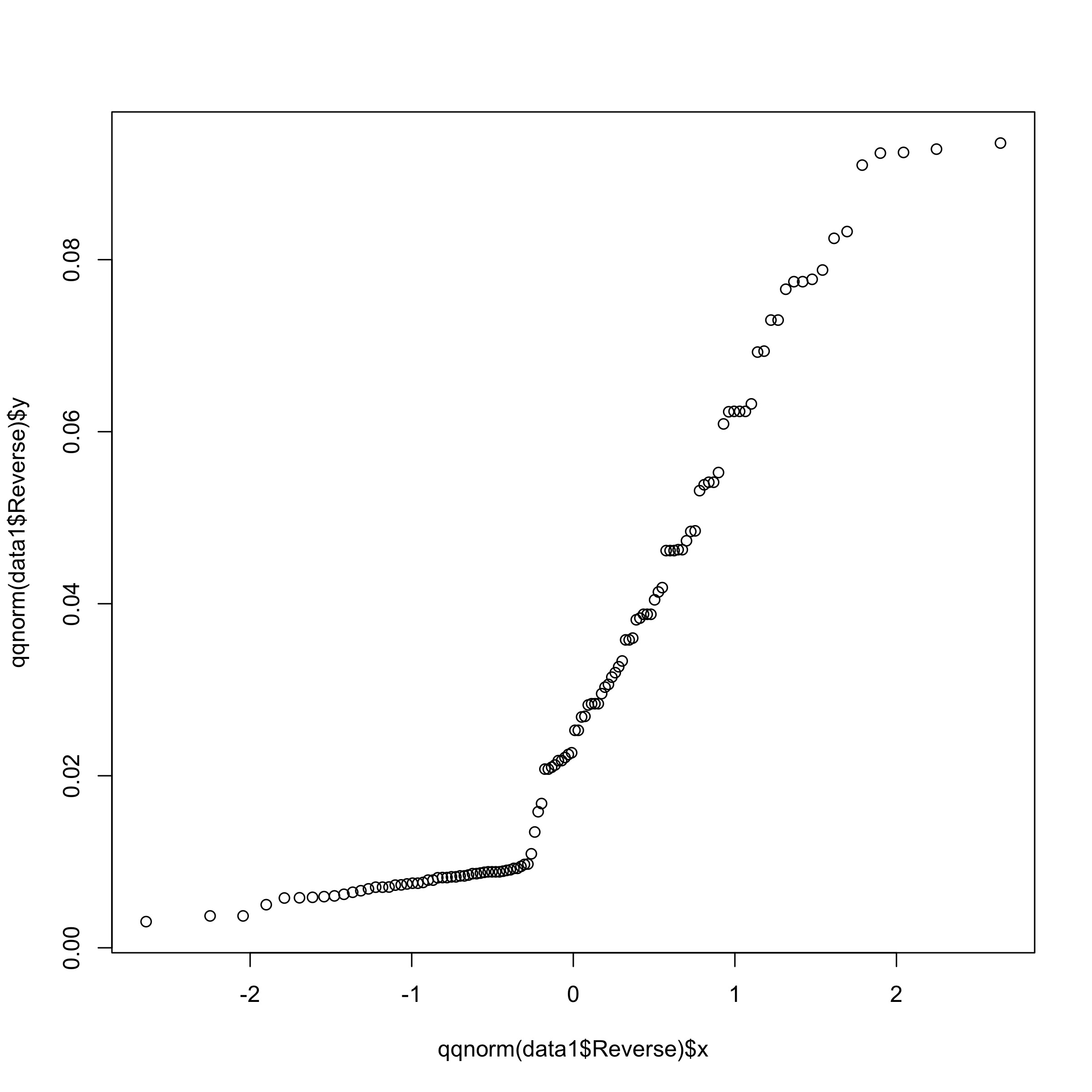

Supplement: Data Sheet 6 — Analysis of all peptide PSM PEP scores. [file DataSheet6.ZIP › Supplementary Data Sheet 6_ Analysis of all peptide PSM PEP scores/PEP_qqnorm/Six_Frame_database/Reverse_PEP_2_reps.jpeg]

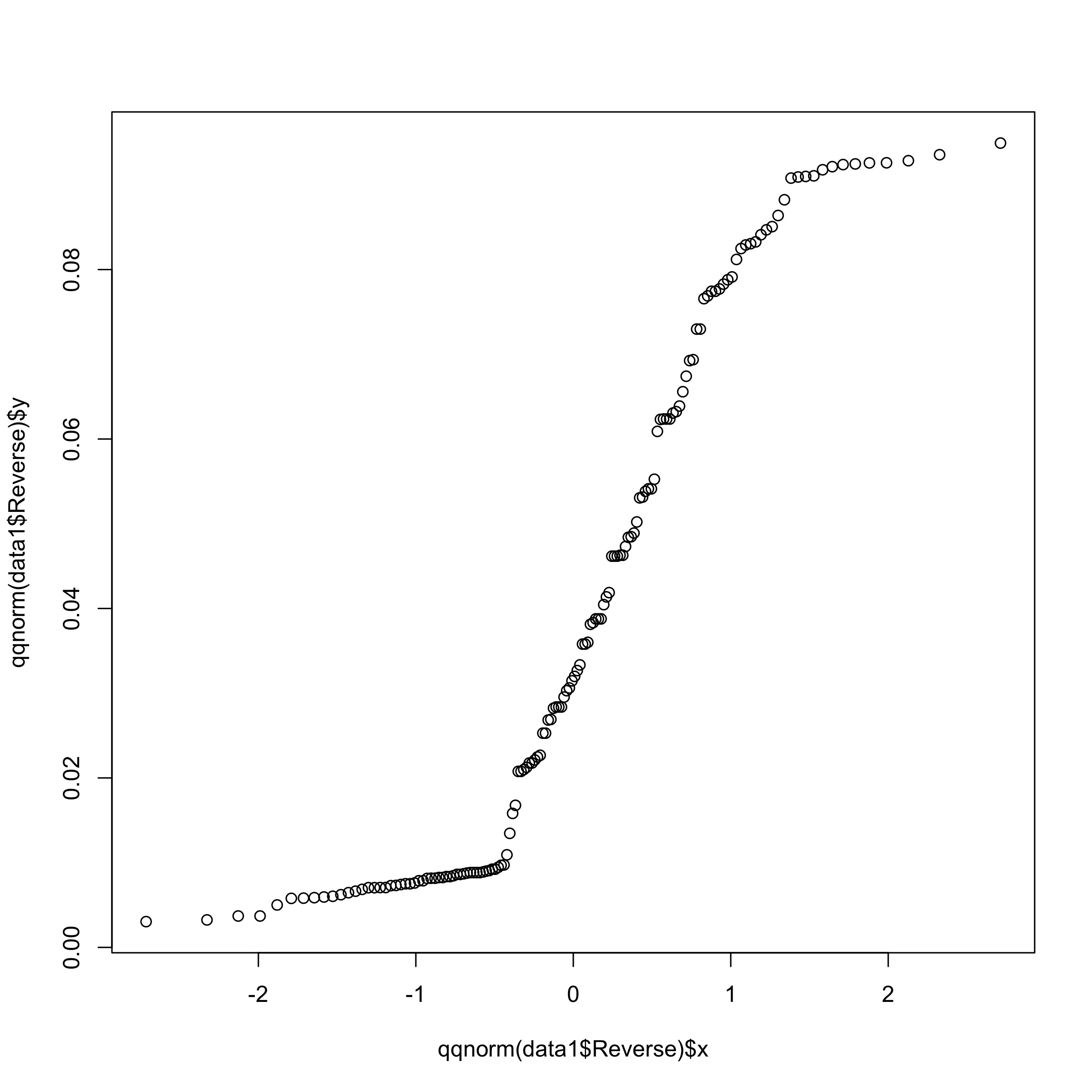

Supplement: Data Sheet 6 — Analysis of all peptide PSM PEP scores. [file DataSheet6.ZIP › Supplementary Data Sheet 6_ Analysis of all peptide PSM PEP scores/PEP_qqnorm/Six_Frame_database/Reverse_PEP_all.jpeg]
